# Efficient synthesis of polyfunctionalized carbazoles and pyrrolo[3,4*-c*]carbazoles via domino Diels–Alder reaction

**DOI:** 10.3762/bjoc.17.159

**Published:** 2021-09-16

**Authors:** Ren-Jie Fang, Chen Yan, Jing Sun, Ying Han, Chao-Guo Yan

**Affiliations:** 1 College of Chemistry and Chemical Engineering, Yangzhou University, Yangzhou 225002, China

**Keywords:** carbazole, chalcone, Diels–Alder reaction, maleimide, pyrrolo[3,4*-c*]carbazole, 3-vinylindole

## Abstract

The *p*-TsOH-catalyzed Diels–Alder reaction of 3-(indol-3-yl)maleimides with chalcone in toluene at 60 °C afforded two diastereoisomers of tetrahydropyrrolo[3,4*-c*]carbazoles, which can be dehydrogenated by DDQ oxidation in acetonitrile at room temperature to give the aromatized pyrrolo[3,4*-c*]carbazoles in high yields. On the other hand, the one-pot reaction of 3-(indol-3-yl)-1,3-diphenylpropan-1-ones with chalcones or benzylideneacetone in acetonitrile in the presence of *p*-TsOH and DDQ resulted in polyfunctionalized carbazoles in satisfactory yields. The reaction mechanism included the DDQ oxidative dehydrogenation of 3-(indol-3-yl)-1,3-diphenylpropan-1-ones to the corresponding 3-vinylindoles, their acid-catalyzed Diels–Alder reaction and sequential aromatization process.

## Introduction

**Figure 1:**
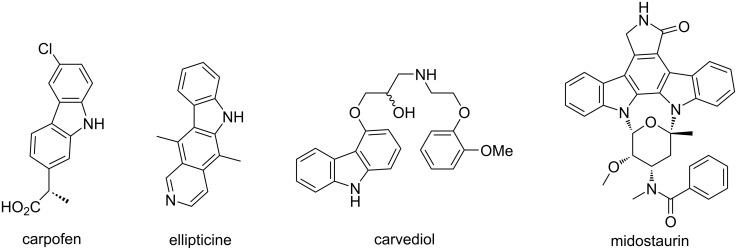
Representative bioactive carbazole derivatives.

Carbazole is one of the most well-known privileged nitrogen-containing heterocycles. The carbazole skeleton is widely occurring in natural alkaloids and pharmacologically active compounds representing a broad spectrum of important bioactivities such as anticancer, antituberculosis, anti-protein kinase C, antipsychotic, and antioxidative activities [1–5]. For some examples, carprofen is a nonsteroidal anti-inflammatory pharmaceutical used to treat joint pain and postoperative pain [6] (Figure 1). Ellipticine was considered to be based mainly on DNA intercalation and topoisomerase II inhibition [7]. Midostaurin and carvediol have been approved by the FDA for tumor therapy and treatment of congestive heart failure [8]. On the other hand, carbazole derivatives also have potential applications in optoelectronic materials, conducting polymers, and synthetic dyes [9–11]. Over the past decades, many efficient synthetic methodologies for functionalized carbazole derivatives have been successfully developed [12–18]. Because indoles are readily available materials, the direct extension of indoles to carbazole skeletons has a great advantage [19–27]. Therefore, the Diels–Alder reaction of activated 2-vinylindolines or 3-vinylindolines with diverse dienophiles has become the most attractive strategy for the synthesis of carbazole derivatives [28–40]. In recent years, by using the one-pot domino synthetic strategy of in situ-generated 2-vinyl- or 3-vinylindolines and sequential Diels–Alder reaction with activated dienophiles, we have successfully developed several efficient synthetic protocols for diversely functionalized tetrahydrocarbazoles and the corresponding carbazole derivatives [41–47]. To further demonstrate the synthetic application of domino Diels–Alder reactions and in continuation of our aim to providing efficient domino reactions for the synthesis of biologically important carbazole derivatives [48–53], herein we wish to report the DDQ-mediated dehydrogenative Diels–Alder reaction of 3-(indol-3-yl)maleimides and benzoyl-substituted 3-ethylindoles with readily available chalcones for the convenient synthesis of polyfunctionalized carbazole derivatives.

## Results and Discussion

**Scheme 1:**
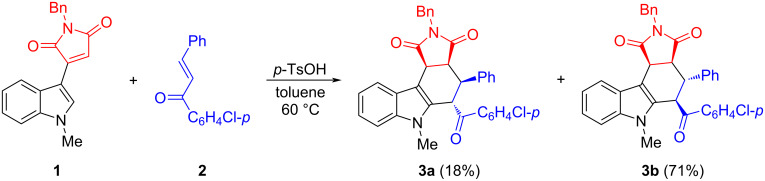
Synthesis of tetrahydropyrrolo[3,4-*c*]carbazoles **3a** and **3b**.

**Figure 2:**
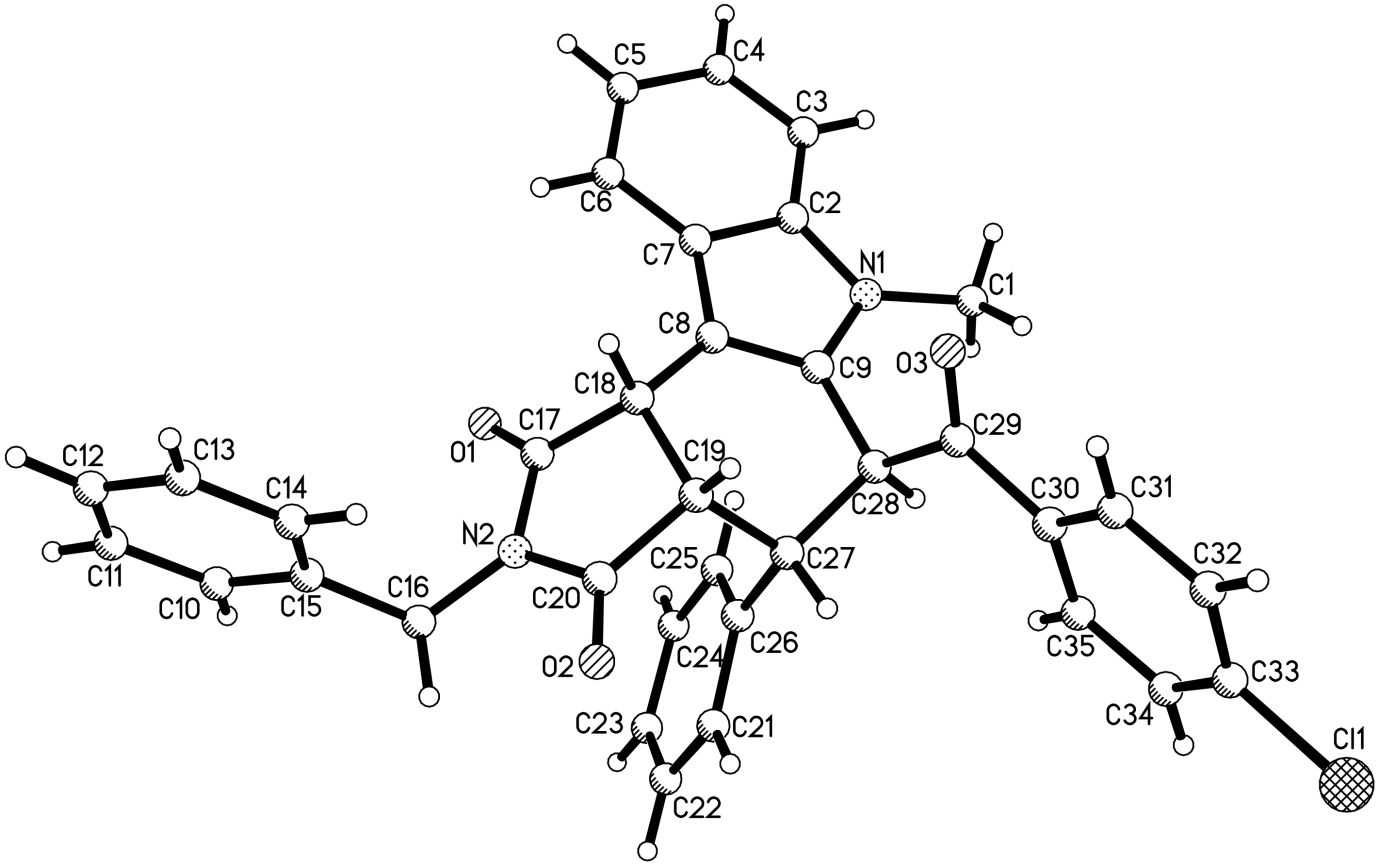
Single crystal structure of the isomer **3a**.

**Figure 3:**
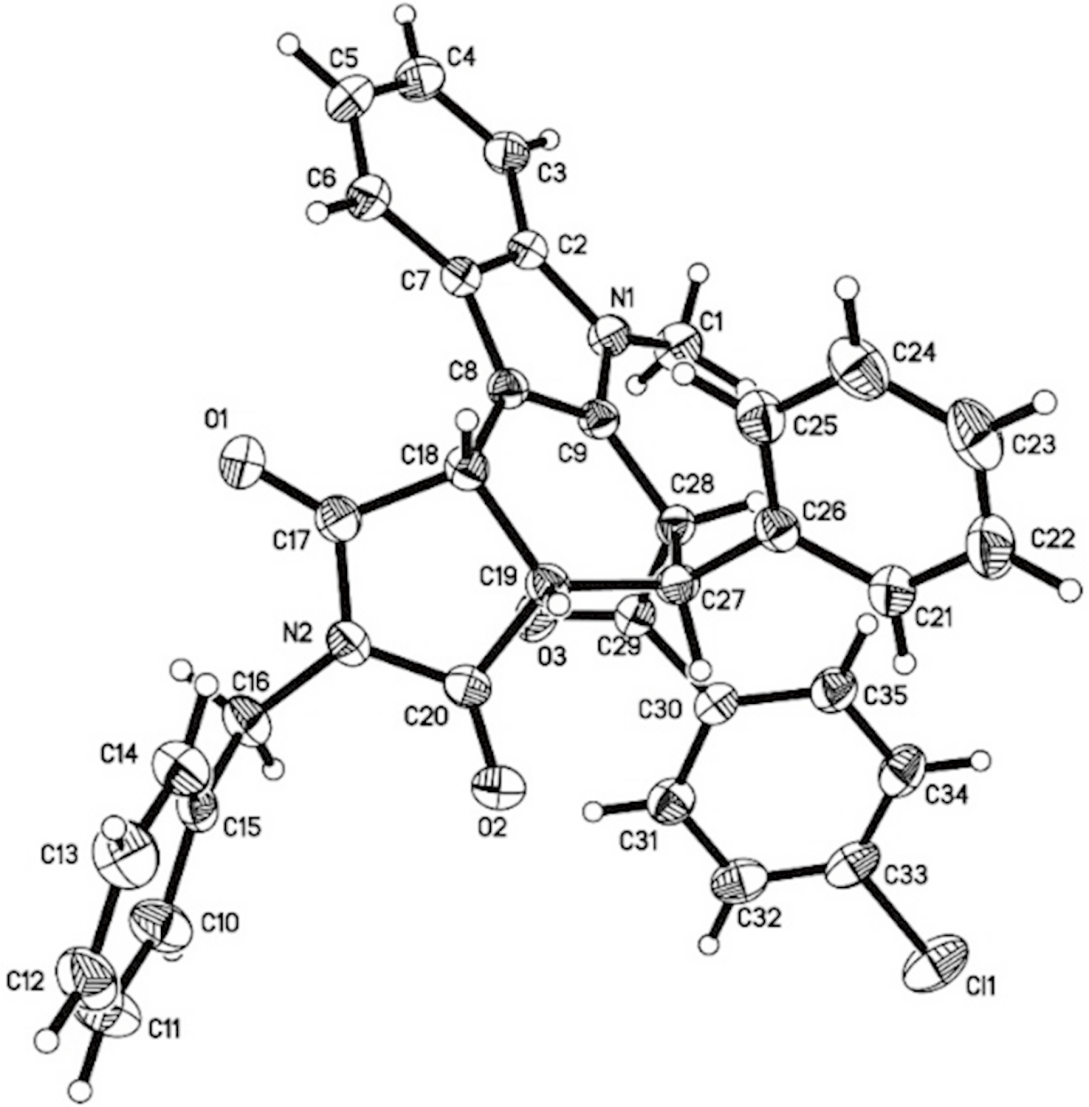
Single crystal structure of the isomer **3b**.

According to our previously established reaction conditions for the preparation of spiro[indoline-3,5'-pyrrolo[3,4-*c*]carbazoles] [48], an equivalent amount of 3-(indol-3-yl)maleimide with chalcone was stirred in toluene at 60 °C for two hours in the presence of *p*-toluenesulfonic acid. After workup, two diastereoisomers **3a** and **3b** of tetrahydropyrrolo[3,4-*c*]carbazoles were successfully isolated in 18% and 71% yields, respectively (Scheme 1). It should be pointed out that nearly no reaction was detected in the absence of *p*-toluenesulfonic acid. The structures of both products were fully characterized by various spectroscopy methods and confirmed by determination of their single crystal structures (Figure 2 and Figure 3).

From Figure 2, it can be seen that the phenyl group and the adjacent *p-*chlorobenzoyl group are *trans*-oriented. Additionally, the phenyl group is *cis*-oriented to the 1-benzylpyrrolidine-2,5-dione ring in compound **3a**. On the other hand, for compound **3b** (Figure 3), it can be seen that the phenyl group is *trans*-oriented to both, the *p*-chlorobenzoyl group and the 1-benzylpyrrolidine-2,5-dione ring in this compound. Thus, the isomers **3a** and **3b** are diastereoisomers. It is known that the starting chalcones usually have *E*-configuration. The phenyl group and *p*-chlorobenzoyl group still exist in the *trans-*position in both diastereoisomers **3a** and **3b** as in the starting chalcone. This result clearly showed that this acid-catalyzed cycloaddition reaction proceeded through a concerted Diels–Alder reaction mechanism.

**Figure 4:**
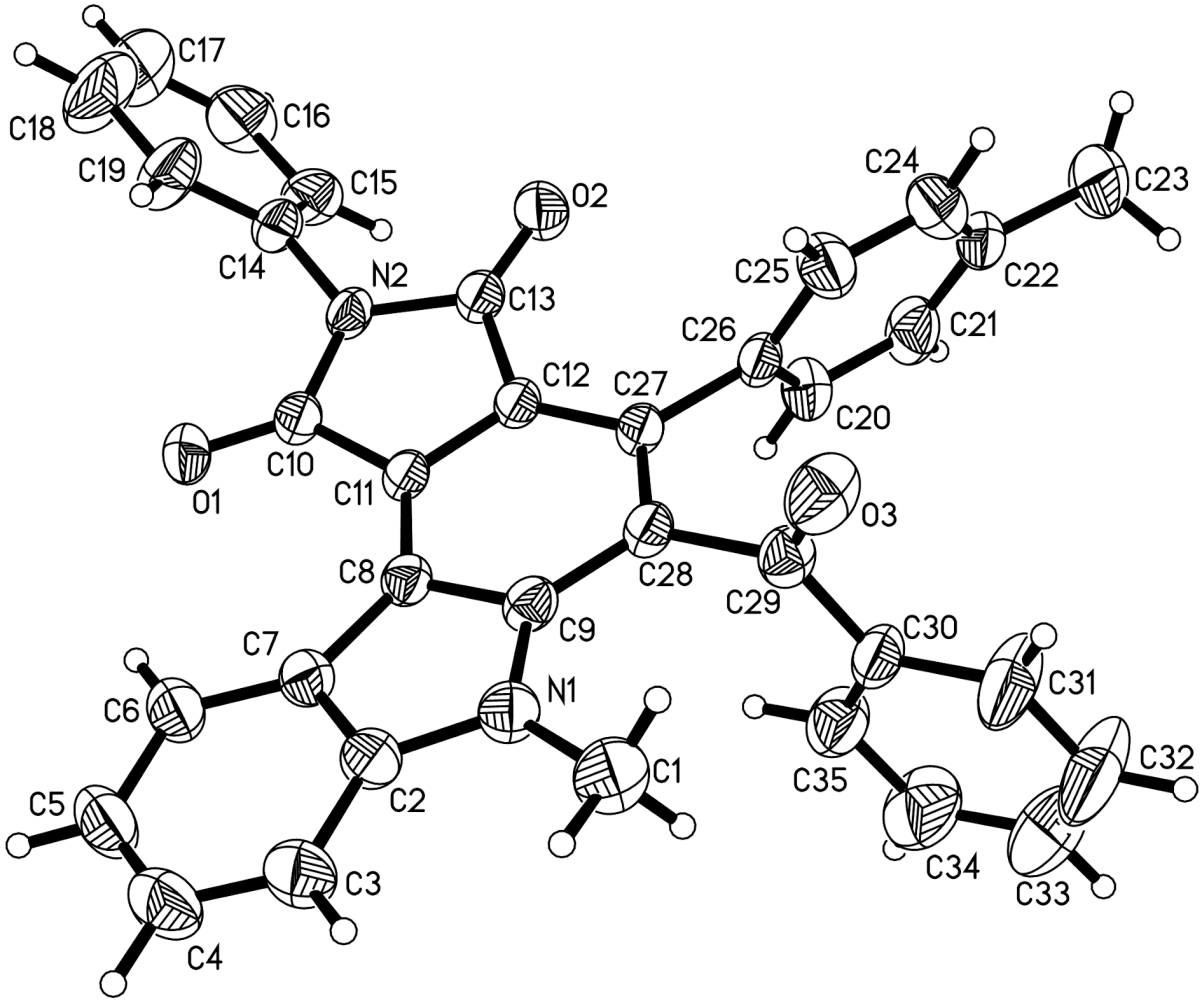
Single crystal structure of the isomer **4g**.

The acid-catalyzed Diels–Alder reaction afforded a mixture of two diastereoisomers, which decreased the synthetic value of the reaction. Thus, after the first step reaction, a DDQ dehydrogenation reaction was carried out in acetonitrile at room temperature. A series of aromatized pyrrolo[3,4-*c*]carbazoles **4a**–**l** were successfully synthesized by the one-pot two-step reaction and the results are summarized in Table 1. All reactions proceeded smoothly to give the corresponding pyrrolo[3,4-*c*]carbazoles **4a–l** in satisfactory yields. Indole itself and *N*-methylindole could also be successfully employed in the reaction. The N–Me, N–Ph, and N–Bn substitution in the maleimide moiety showed only a marginal effect on the reaction outcome. Various chalcones with electron-donating methyl and methoxy groups and electron-withdrawing *m*-chloro and *p*-chloro substituents gave the products in good yields. However, the nitro-substituted chalcone gave the product **4h** in a slightly lower yield. The structures of the pyrrolo[3,4-*c*]carbazoles **4a**–**l** were established by various spectroscopy methods. Further, the single crystal structure of compound **4g** was determined by X-ray diffraction (Figure 4). It can be seen that the ring of pyrrolo[3,4-*c*]carbazole exists in a slightly twisted plane. The dihedral angles of the phenyl and the benzoyl group to the central benzene ring are 72.018° and 88.402°.

**Table 1:** Synthesis of pyrrolo[3,4-*c*]carbazoles **4a–l**.^a^

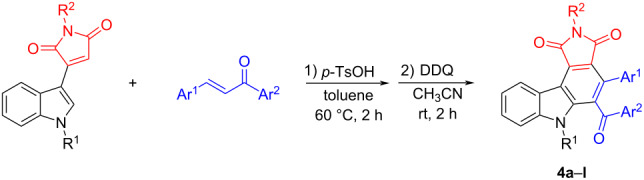						

entry	compound	R^1^	R^2^	Ar^1^	Ar^2^	yield (%)^b^

1	**4a**	H	Ph	*p*-CH_3_C_6_H_4_	C_6_H_5_	87
2	**4b**	CH_3_	CH_3_	*p*-ClC_6_H_4_	C_6_H_5_	89
3	**4c**	CH_3_	CH_3_	C_6_H_5_	*p*-CH_3_C_6_H_5_	92
4	**4d**	CH_3_	CH_3_	*p*-CH_3_C_6_H_4_	*p*-CH_3_C_6_H_4_	91
5	**4e**	CH_3_	Ph	C_6_H_5_	CH_3_	85
6	**4f**	CH_3_	Ph	C_6_H_5_	C_6_H_5_	84
7	**4g**	CH_3_	Ph	*p*-CH_3_C_6_H_4_	C_6_H_5_	93
8	**4h**	CH_3_	Ph	*m*-NO_2_C_6_H_4_	*p*-CH_3_C_6_H_4_	76
9	**4i**	CH_3_	Ph	*p*-CH_3_C_6_H_4_	*p*-CH_3_OC_6_H_4_	86
10	**4j**	CH_3_	Ph	*p*-CH_3_C_6_H_4_	*m*-ClC_6_H_4_	84
11	**4k**	CH_3_	Bn	*p*-CH_3_C_6_H_4_	C_6_H_5_	93
12	**4l**	CH_3_	Bn	C_6_H_5_	*p*-ClC_6_H_4_	87

^a^Reaction conditions: 1) 3-(indol-3-yl)maleimide (1.0 mmol), chalcone (1.0 mmol), toluene (10.0 mL), *p-*TsOH (0.2 mmol), 80 °C, 2 h; 2) DDQ (1.2 mmol), CH_3_CN (10.0 mL), rt, 2 h. ^b^Isolated yields.

To further expand the scope of this domino Diels–Alder reaction, another kind of 3-vinylindoles was employed in the one-pot reaction. First, the 3-(indol-3-yl)-1,3-diphenylpropan-1-ones prepared through Friedel–Crafts alkylation of indole with chalcones, were oxidized by DDQ in acetonitrile to generate in situ the expected active diene, indole-substituted chalcones. Then, the *p*-TsOH-catalyzed Diels–Alder reaction of indole-chalcones with second chalcones and sequential aromatization through DDQ dehydrogenation resulted in the polyfunctionalized carbazoles **6a**–**l** in good yields (Table 2). Additionally, the similar reaction with benzylideneacetone gave the desired carbazoles **6m** and **6n** albeit in significantly lower yields. Thus, this one-pot domino reaction successfully constructed carbazoles with four substituents on the benzene ring from the corresponding indole derivatives with two molecules of chalcones. It should be pointed out that the Diels–Alder reaction resulted in a complex mixture comprising four diastereoisomers of the tetrahydrocarbazoles, which was very difficult to separate. After oxidation with DDQ, the aromatized carbazole derivatives **6a**–**n** were easily obtained as single products in good yields. The chemical structures of the carbazoles were fully characterized by ^1^H NMR, ^13^C NMR, IR, and HRMS spectra.

**Table 2:** Synthesis of the polysubstituted carbazoles **6a**–**n**.^a^

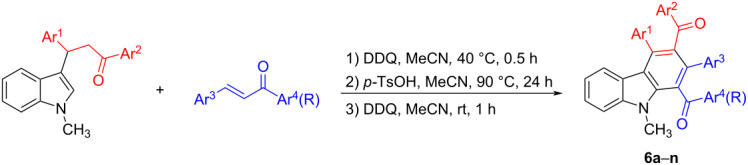						

entry	compound	Ar^1^	Ar^2^	Ar^3^	Ar^4^ (R)	yield (%)^b^

1	**6a**	C_6_H_5_	C_6_H_5_	C_6_H_5_	C_6_H_5_	61
2	**6b**	*p*-CH_3_C_6_H_4_	*p*-CH_3_C_6_H_4_	*p*-CH_3_C_6_H_4_	*p*-CH_3_C_6_H_4_	63
3	**6c**	*p*-CH_3_C_6_H_4_	*p*-CH_3_C_6_H_4_	*p*-ClC_6_H_4_	*p*-CH_3_C_6_H_4_	68
4	**6d**	*p*-CH_3_C_6_H_4_	*p*-CH_3_C_6_H_4_	*o*-CH_3_C_6_H_4_	*p*-CH_3_OC_6_H_4_	64
5	**6e**	*p*-CH_3_OC_6_H_4_	*p*-CH_3_C_6_H_4_	*o*-CH_3_C_6_H_4_	*p*-CH_3_OC_6_H_4_	62
6	**6f**	*p*-CH_3_OC_6_H_4_	*p*-CH_3_C_6_H_4_	*p*-CH_3_OC_6_H_4_	*p*-ClC_6_H_4_	66
7	**6g**	*p*-ClC_6_H_4_	*p*-CH_3_C_6_H_4_	C_6_H_5_	C_6_H_5_	59
8	**6h**	*p*-ClC_6_H_4_	*p*-CH_3_C_6_H_4_	*p*-ClC_6_H_4_	*p*-CH_3_C_6_H_4_	60
9	**6i**	*p*-ClC_6_H_4_	*p*-CH_3_C_6_H_4_	*p*-NO_2_C_6_H_4_	*p*-CH_3_OC_6_H_4_	69
10	**6j**	*o*-ClC_6_H_4_	*p*-CH_3_C_6_H_4_	*p*-CH_3_OC_6_H_4_	*p*-ClC_6_H_4_	63
11	**6k**	*p-*CH_3_OC_6_H_4_	*p*-ClC_6_H_4_	*m*-ClC_6_H_4_	*p*-CH_3_C_6_H_4_	61
12	**6l**	*p*-CH_3_OC_6_H_4_	*p*-ClC_6_H_4_	*p*-CH_3_OC_6_H_4_	*p*-ClC_6_H_4_	62
13	**6m**	C_6_H_5_	C_6_H_5_	C_6_H_5_	CH_3_	31
14	**6n**	*p*-ClC_6_H_4_	*p*-CH_3_C_6_H_4_	C_6_H_5_	CH_3_	32

^a^Reaction conditions: 1) 3-(indol-3-yl)-1,3-diphenylpropan-1-one (0.6 mmol), chalcone (0.5 mmol), DDQ (0.72 mmol), MeCN (15.0 mL), rt, 0.5 h; 2) *p*-TsOH (0.06 mmol), reflux, 4 h; 3) DDQ (0.6 mmol), rt, 1 h. ^b^Isolated yields.

**Scheme 2:**
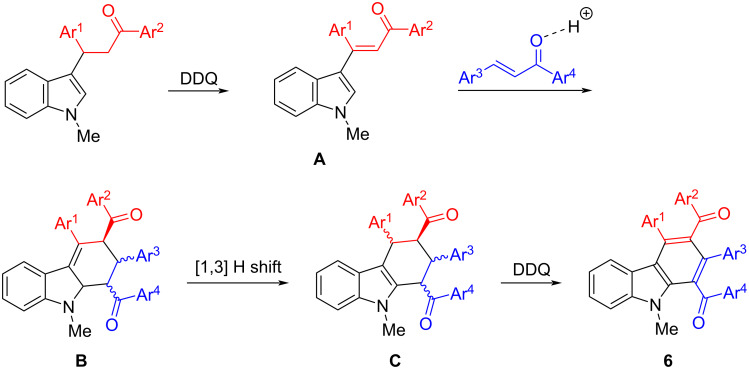
Proposed domino reaction mechanism for the formation of carbazoles **6**.

To explain the formation of the products, a plausible reaction mechanism was proposed in Scheme 2 on the basis of the previously reported reaction [48,53]. Firstly, the DDQ oxidative dehydrogenation of 3-(indol-3-yl)-1,3-diphenylpropan-1-one gave the expected indole-substituted chalcone **A**, which comprises the desired 3-vinylindole scaffold as the reactive diene. In the meantime, the carbonyl group of the chalcone is protonated to give the activated dienophile in the presence of *p*-toluenesulfonic acid. Secondly, the Diels–Alder reaction of indole-chalcone **A** with the dienophile results in the tetrahydrocarbazole **B** having an exocyclic C=C bond. Thirdly, a new tetrahydrocarbazole intermediate **C** is formed by a 1,3-H shifting process. The resulting tetrahydrocarbazole intermediate (**C**) might be a mixture of several possible diastereoisomers because it has four substituents on the cyclohexenyl ring. After a further DDQ oxidation, the aromatized carbazole **6** is successfully produced as the final product.

## Conclusion

In summary, we have investigated the domino Diels–Alder reaction of 3-(indol-3-yl)maleimides and in situ-generated indole-chalcones with dienophilic chalcones. This one-pot two-step reaction successfully provided the polyfunctionalized carbazole derivatives in an extremely simple and highly efficient fashion. This protocol has the advantages of using readily available starting reagents, simple manufacture, high efficiency and atomic economy. The unusual feature of this reaction is the normal electron-demand Diels–Alder reaction between electron-deficient dienes such as (3-(indol-3-yl)maleimides and indole-chalcone to electron-deficient dienophilic chalcones. The potential applications of this reaction in organic and medicinal chemistry might be significant.

## Experimental

**1. General procedure for the preparation of the carbazoles 4a–l:** To a round-bottomed flask was added 3-(indol-3-yl)maleimide (1.0 mmol), chalcone (1.0 mmol), *p-*toluenesulfonic acid (0.2 mmol), and toluene (10.0 mL). The solution was heated to 60 °C for two hours. After removing the solvent by rotatory evaporation, DDQ (1.2 mmol) and acetonitrile (10.0 mL) were added and the mixture was stirred at room temperature for two hours. After removing the solvent, the residue was subjected to column chromatography with petroleum ether and ethyl acetate 8:1 (v/v) as eluent to give the pure products for analysis.

**5-Benzoyl-2-phenyl-4-(*p*-tolyl)pyrrolo[3,4-*c*]carbazole-1,3(2*H*,6*H*)-dione (4a)**: green solid, 440 mg, 87%; mp 234–236 °C; ^1^H NMR (400 MHz, CDCl_3_) δ 9.24 (s, 1H, ArH), 9.15 (d, *J* = 8.0 Hz, 1H, ArH), 7.60–7.56 (m, 1H, ArH), 7.53–7.44 (m, 7H, ArH), 7.42–7.31 (m, 3H, ArH), 7.21–7.14 (m, 4H, ArH), 6.92 (d, *J* = 7.6 Hz, 2H, ArH), 2.18 (s, 3H, CH_3_); ^13^C {^1^H} NMR (100 MHz, CDCl_3_) δ 198.0, 167.3, 142.9, 141.7, 138.2, 137.6, 137.2, 132.9, 131.9, 131.7, 130.7, 129.1, 129.0, 128.8, 128.2, 127.9, 127.8, 127.7, 126.7, 126.1, 125.6, 121.7, 120.7, 120.3, 119.2, 111.3, 21.1; IR (KBr) ν: 2988, 1786, 1734, 1611, 1485, 1456, 1357, 1314, 1185, 1021, 988, 786, 734 cm^−1^; HRMS–ESI-TOF (*m*/*z*): [M + Na]^+^ calcd for C_34_H_22_NaN_2_O_3_, 529.1523; found, 529.1512.

**2. General procedure for the preparation of carbazoles 6a–n:** To a round-bottomed flask were added 3-(indol-3-yl)-1,3-diphenylpropan-1-one (0.6 mmol), DDQ (0.72 mmol), and acetonitrile (15.0 mL). The mixture was stirred at room temperature for 30 min. Then, the chalcone (0.5 mmol) and *p*-toluenesulfonic acid (0.06 mmol) were added and the solution was refluxed for four hours. After cooling to room temperature, DDQ (0.6 mmol) was added and the mixture was stirred at room temperature for one hour. After removing the solvent by rotatory evaporation at reduced pressure, the residue was subjected to column chromatography with a mixture of petroleum ether, ethyl acetate and methylene dichloride 20:1:5 (v/v/v) to give the pure products for analysis.

**(9-Methyl-2,4-diphenyl-9*H*-carbazole-1,3-diyl)bis(phenylmethanone) (6a):** White solid, 165 mg, 61%; mp 249–251 °C; ^1^H NMR (400 MHz, CDCl_3_) δ 7.64 (d, *J* = 7.6 Hz, 3H, ArH), 7.43 (t, *J* = 7.8 Hz, 2H, ArH), 7.39–7.34 (m, 6H, ArH), 7.28–7.24 (m, 3H, ArH), 7.09 (t, *J* = 7.6 Hz, 2H, ArH), 7.01 (s, 2H, ArH), 6.96 (t, *J* = 7.8 Hz, 2H, ArH), 6.84 (d, *J* = 6.8 Hz, 4H, ArH), 3.64 (s, 3H, CH_3_); ^13^C NMR (400 MHz, CDCl_3_) δ 198.5, 198.4, 142.4, 138.9, 138.7, 137.6, 137.5, 136.8, 135.9, 135.2, 133.3, 132.2, 131.5, 131.4, 129.5, 129.2, 128.3, 128.2, 127.8, 127.6, 127.0, 126.9, 126.4, 122.4, 122.0, 121.8, 121.6, 119.6, 108.7, 32.1; IR (KBr) ν: 3057, 3023, 2907, 2360, 2339, 1720, 1605, 1482, 1320, 1267, 1172, 1009, 936, 805, 743, 612, 447 cm^−1^; HRMS–ESI (*m*/*z*): [M + Na]^+^ calcd for C_39_H_27_NO_2_, 564.1934; found, 564.1926.

The crystallographic data of the compounds **3a** (CCDC 2099074), **3b** (CCDC 2099075), and **4g** (CCDC 2099076) have been deposited at the Cambridge Crystallographic Database Centre (http://www.ccdc.cam.ac.uk).

## Supporting Information

File 1 Characterization data and ^1^H NMR, ^13^C NMR, and HRMS spectra of the synthesized compounds.
