# Supplementary material for: Efficient synthesis of polyfunctionalized carbazoles and pyrrolo[3,4-c]carbazoles via domino Diels–Alder reaction
Source: Beilstein J Org Chem. 2021 Sep 16;17:2425–32. doi: 10.3762/bjoc.17.159 (PMC8450976; doi:10.3762/bjoc.17.159)
Supplement: File 1 — Characterization data and 1H NMR, 13C NMR, and HRMS spectra of the synthesized compounds. [file Beilstein_J_Org_Chem-17-2425-s001.pdf]

BEILSTEIN JOURNAL OF ORGANIC CHEMISTRY

Supporting Information

for

Efficient synthesis of polyfunctionalized carbazoles and pyrrolo[3,4-*c*]carbazoles via domino Diels–Alder reaction

Ren-Jie Fang, Chen Yan, Jing Sun, Ying Han and Chao-Guo Yan

Beilstein J. Org. Chem. **2021,** *17,* 2425–2432. doi:10.3762/bjoc.17.159

Characterization data and ^{1}H NMR, ^{13}C NMR, and HRMS spectra of the synthesized compounds

License and Terms: This is a supporting information file under the terms of the Creative Commons Attribution License (https://creativecommons.org/licenses/by/4.0). Please note that the reuse, redistribution and reproduction in particular requires that the author(s) and source are credited and that individual graphics may be subject to special legal provisions.
The license is subject to the *Beilstein Journal of Organic Chemistry* terms and conditions: (https://www.beilstein-journals.org/bjoc/terms)

***rel-(3aS,4S,5S,10cS)*-2-Benzyl-5-(4-chlorobenzoyl)-6-methyl-4-phenyl-4,5,6,10c-tetrahydropyrrolo[3,4-*c*]carbazole-1,3(2*H*,3a*H*)-dione (3a)**: white solid, 100 mg, 18%, m.p. 221-223 °C; ^{1}H NMR (400 MHz, $CDCl_3$) δ: 8.15 (d, *J* = 7.6 Hz, 1H, ArH), 7.93 (d, *J* = 8.4 Hz, 2H, ArH), 7.54 (d, *J* = 7.6 Hz, 2H, ArH), 7.35-7.30 (m, 4H, ArH), 7.28-7.24 (m, 7H, ArH), 7.10-7.08 (m, 2H, ArH), 4.93 (s, 1H, CH), 4.59 (s, 2H, CH), 4.41 (d, *J* = 8.0 Hz, 1H, CH), 4.37 (s, 1H, CH), 3.45 (s, 3H, CH_3), 3.37 (d, *J* = 8.0 Hz, 1H, CH); ^{13}C {^{1}H} NMR (100 MHz, $CDCl_3$) δ: 196.1, 176.7, 175.8, 142.9, 140.2, 137.9, 135.7, 133.6, 130.9, 130.0, 129.4, 129.3, 128.8, 128.4, 127.8, 127.7, 126.9, 125.9, 122.4, 120.6, 120.0, 109.1, 106.3, 45.7, 45.6, 42.6, 39.9, 38.3, 29.4; IR (KBr) υ: 2988, 2876, 1832, 1711, 1632, 1485, 1446, 1354, 1312, 1186, 1034, 967, 788, 732 cm^{-1}; MS (*m*/*z*): HRMS (ESI-TOF) Calcd. for $C_{35}H_{27}ClNaN_2O_3$ ([M+Na]$^+$): 581.1602, Found: 581.1607.

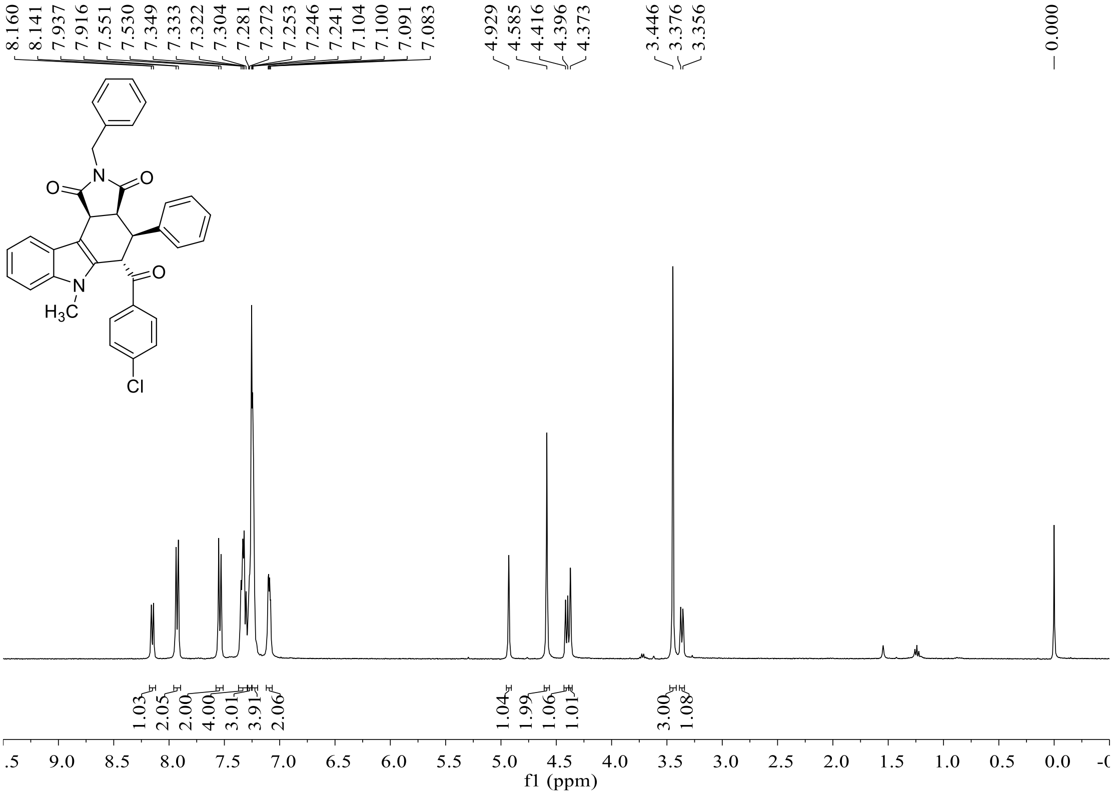

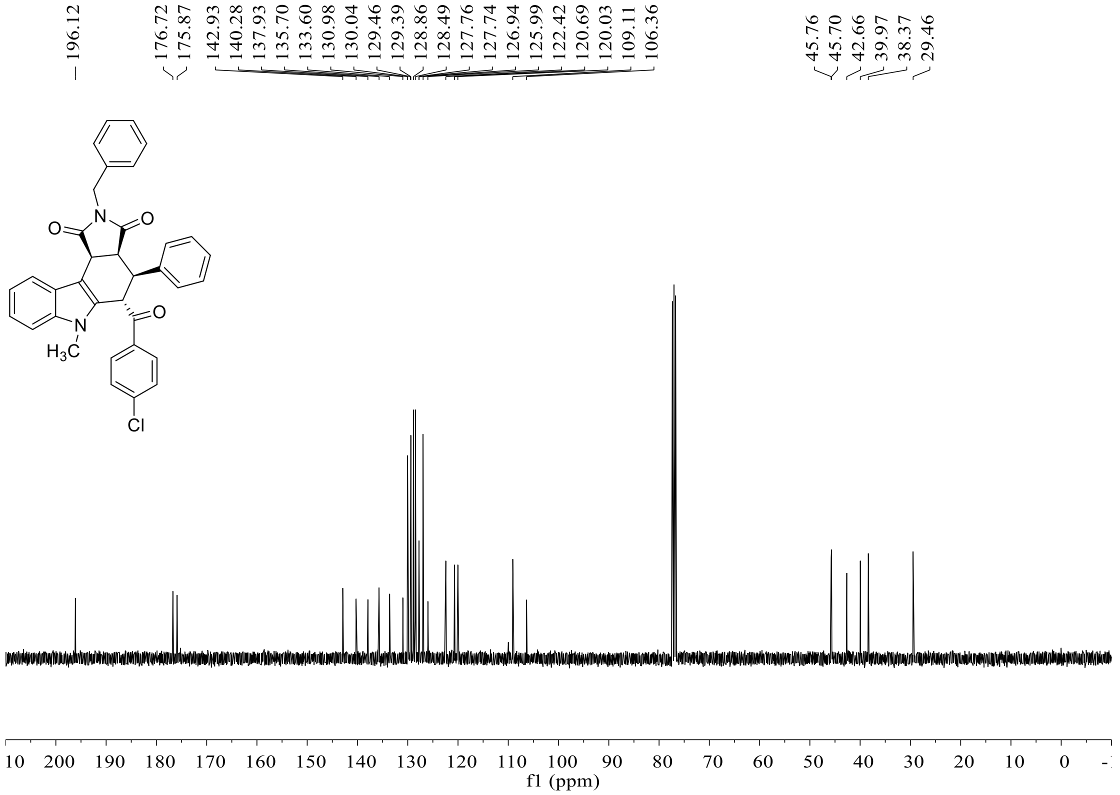

YC7 #60 RT: 0.76 AV: 1 NL: 1.81E+005
T: FTMS + p ESI Full ms [100.0000-1500.0000]

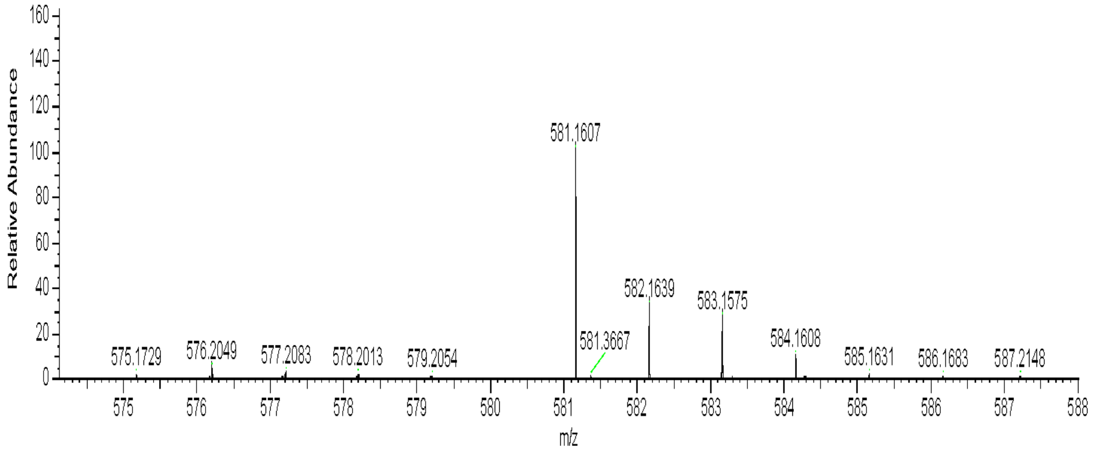

***rel-(3aS,4R,5R,10cS)*-2-Benzyl-5-(4-chlorobenzoyl)-6-methyl-4-phenyl-4,5,6,10c-tetrahydropyrrolo[3,4-*c*]carbazole-1,3(2*H*,3a*H*)-dione (3b)**: white solid, 396 mg, 71%, m.p. 227-229 °C; ^{1}H NMR (400 MHz, $CDCl_3$) δ: 8.15 (d, *J* = 7.6 Hz, 1H, ArH), 7.94 (d, *J* = 8.8 Hz, 2H, ArH), 7.52 (d, *J* = 8.4 Hz, 2H, ArH), 7.37-7.30 (m, 2H, ArH), 7.28-7.23 (m, 1H, ArH), 7.16-7.14 (m, 3H, ArH), 7.08-6.98 (m, 7H, ArH), 5.01 (s, 1H, CH), 4.39 (d, *J* = 8.0 Hz, 1H, CH), 4.13 (d, *J* = 14.0 Hz, 1H, CH), 4.07 (d, *J* = 14.0 Hz, 1H, CH), 3.98 (d, *J* = 6.4 Hz, 1H, CH), 3.58 (t, *J* = 7.6 Hz, 1H, CH), 3.46 (s, 3H, CH_3); ^{13}C {^{1}H} NMR (100 MHz, $CDCl_3$) δ: 195.2, 176.5, 175.6, 140.8, 138.0, 137.9, 135.2, 133.0, 132.0, 130.0, 129.6, 128.8, 128.7, 128.4, 128.2, 127.5, 126.3, 122.4, 121.1, 120.1, 109.0, 106.4, 45.7, 43.5, 42.5, 41.9, 38.9, 29.7; IR (KBr) υ: 2976, 2854, 1841, 1722, 1627, 1479, 1450, 1361, 1322, 1179, 1045, 966, 779, 745 cm^{-1}; MS (*m/z*): HRMS (ESI-TOF) Calcd. for $C_{35}H_{27}ClNaN_2O_3$ ($[M+Na]^+$): 581.1602, Found: 581.1608.

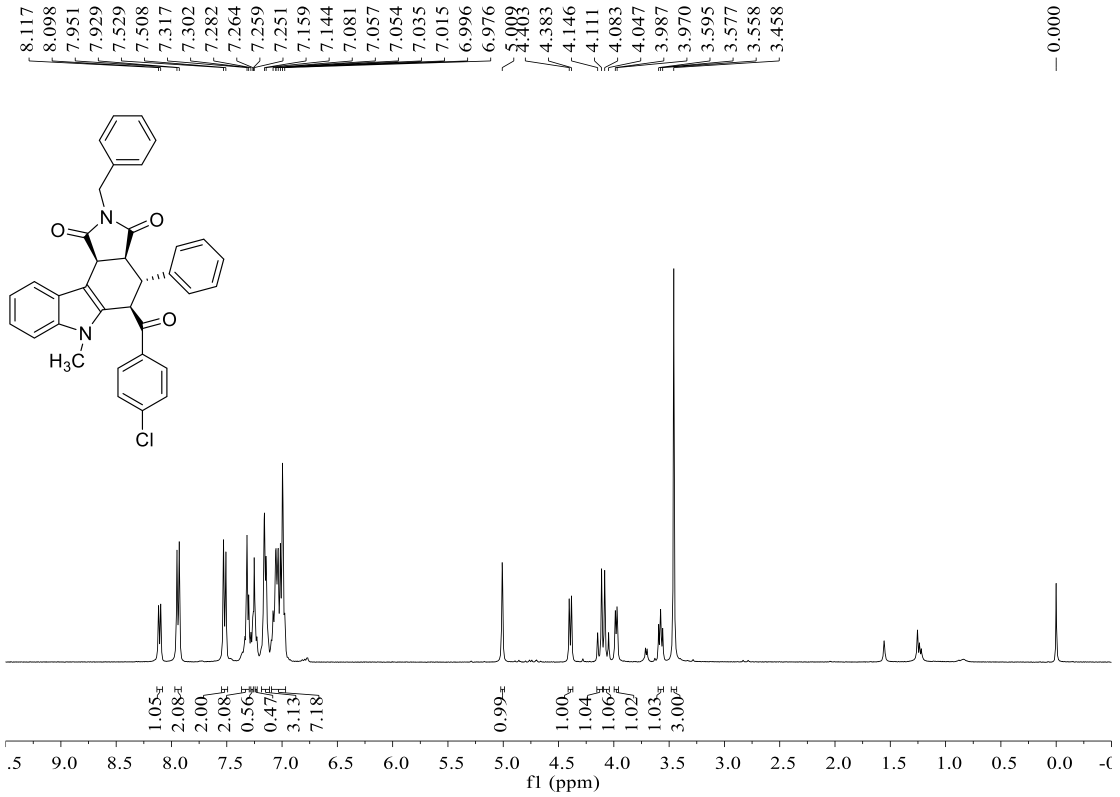

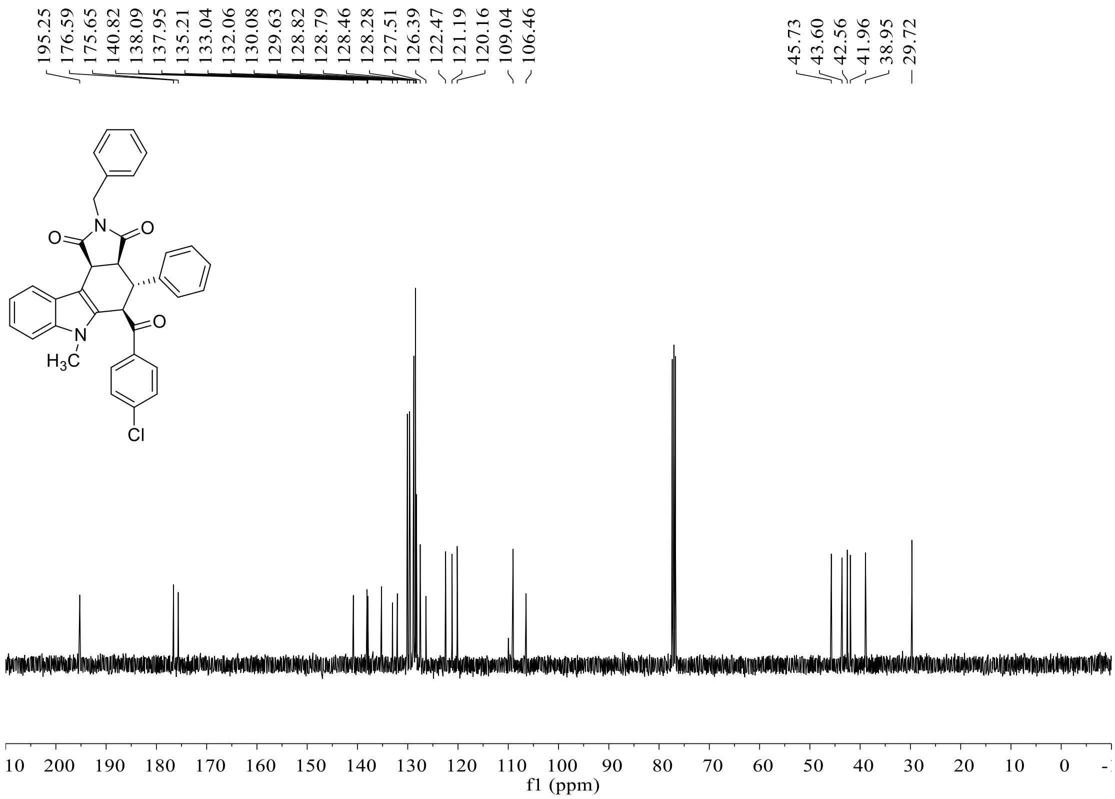
195.25
176.59
175.65
140.82
138.09
137.95
135.21
133.04
132.06
130.08
129.63
128.82
128.79
128.46
128.28
127.51
126.39
122.47
121.19
120.16
109.04
106.46
45.73
43.60
42.56
41.96
38.95
29.72
O
N
O
O
N
H3C
Cl
f1 (ppm)

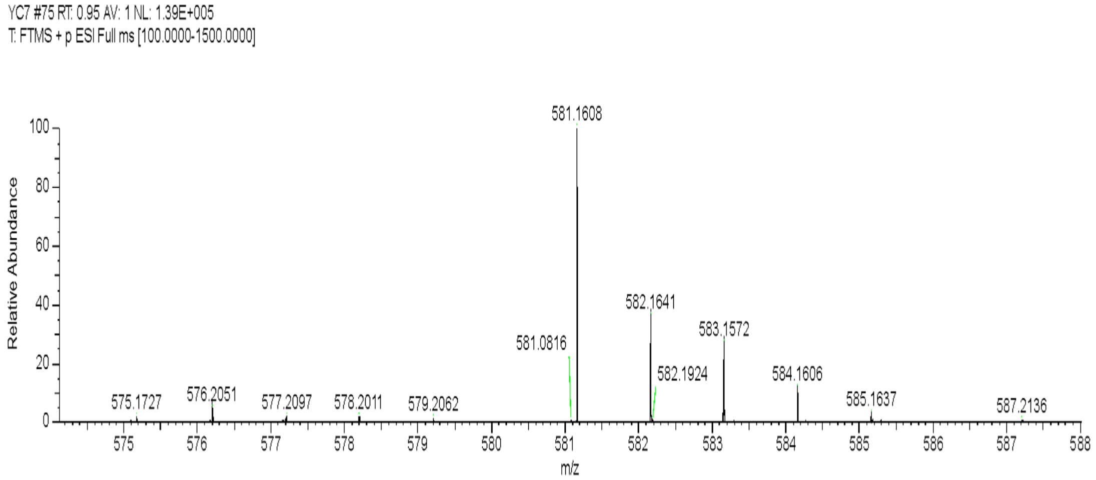
YC7 #75 RT: 0.95 AV: 1 NL: 1.39E+005
T: FTMS + p ESI Full ms [100.0000-1500.0000]
581.1608
582.1641
583.1572
581.0816
582.1924
584.1606
585.1637
575.1727
576.2051
577.2097
578.2011
579.2062
587.2136
Relative Abundance
m/z

5-Benzoyl-2-phenyl-4-(*p*-tolyl)pyrrolo[3,4-*c*]carbazole-1,3(2*H*,6*H*)-dione (4a): green solid, 440 mg, 87%, m.p. 234-236 °C; ^{1}H NMR (400 MHz, $CDCl_3$) δ: 9.24 (s, 1H, ArH), 9.15 (d, J = 8.0 Hz, 1H, ArH), 7.60-7.56 (m, 1H, ArH), 7.53-7.44 (m, 7H, ArH), 7.42-7.31 (m, 3H, ArH), 7.21-7.14 (m, 4H, ArH), 6.92 (d, J = 7.6 Hz, 2H, ArH), 2.18 (s, 3H, CH_3); ^{13}C {^{1}H} NMR (100 MHz, $CDCl_3$) δ: 198.0, 167.3, 142.9, 141.7, 138.2, 137.6, 137.2, 132.9, 131.9, 131.7, 130.7, 129.1, 129.0, 128.8, 128.2, 127.9, 127.8, 127.7, 126.7, 126.1, 125.6, 121.7, 120.7, 120.3, 119.2, 111.3, 21.1; IR (KBr) υ: 2988, 1786, 1734, 1611, 1485, 1456, 1357, 1314, 1185, 1021, 988, 786, 734 cm^{-1}; MS (m/z): HRMS (ESI-TOF) Calcd. for $C_{34}H_{22}NaN_2O_3$ ($[M+Na]^+$): 529.1523, Found: 529.1512.

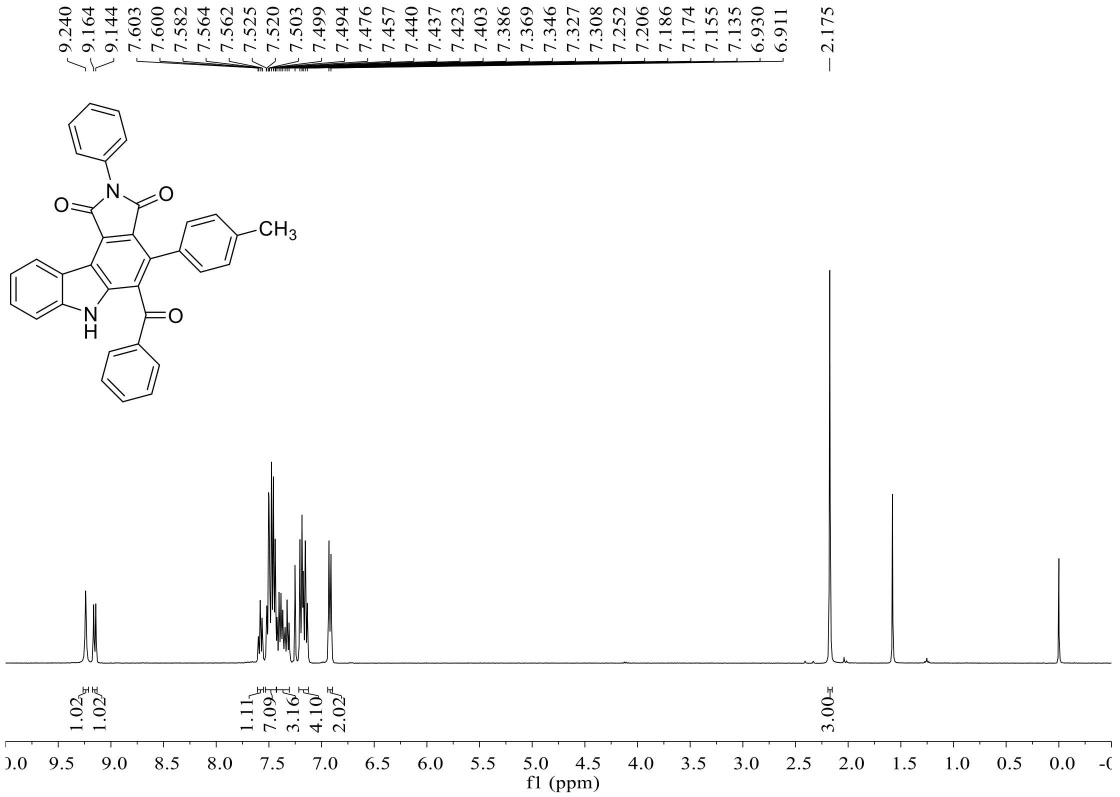

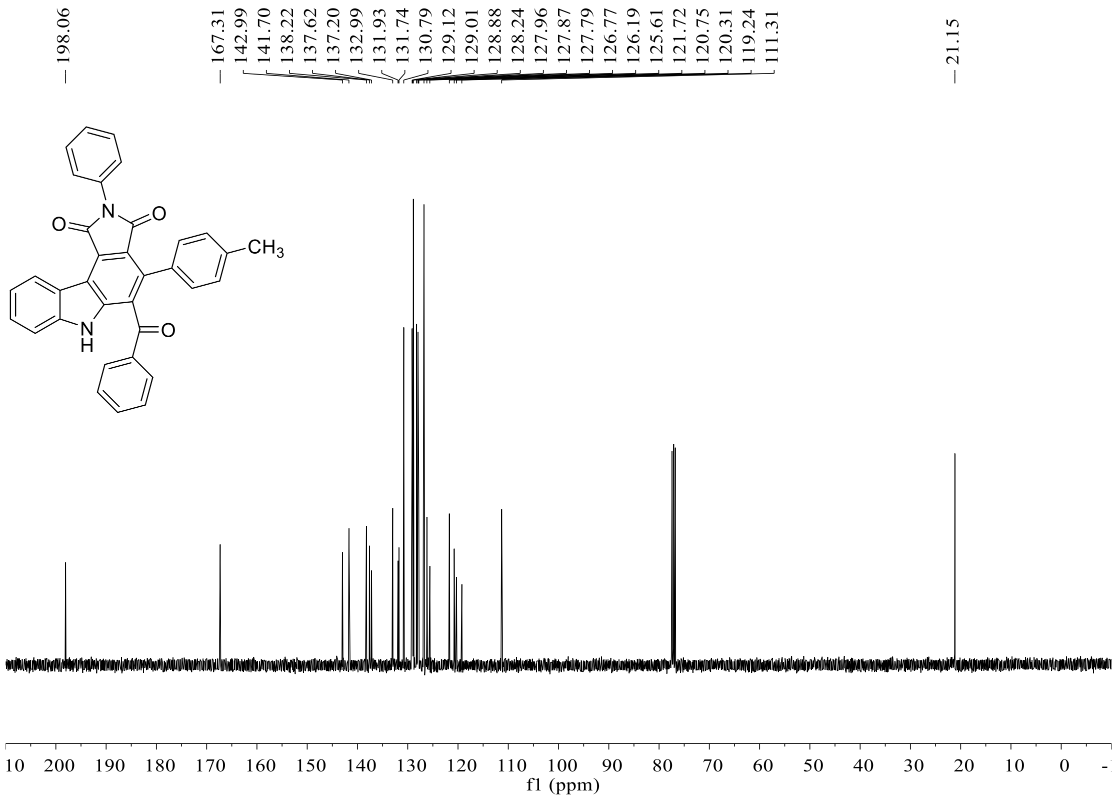
198.06
167.31
142.99
141.70
138.22
137.62
137.20
132.99
131.93
131.74
130.79
129.12
129.01
128.88
128.24
127.96
127.87
127.79
126.77
126.19
125.61
121.72
120.75
120.31
119.24
111.31
21.15
O
N
O
CH3
N
H
O
10 200 190 180 170 160 150 140 130 120 110 100 90 80 70 60 50 40 30 20 10 0
f1 (ppm)

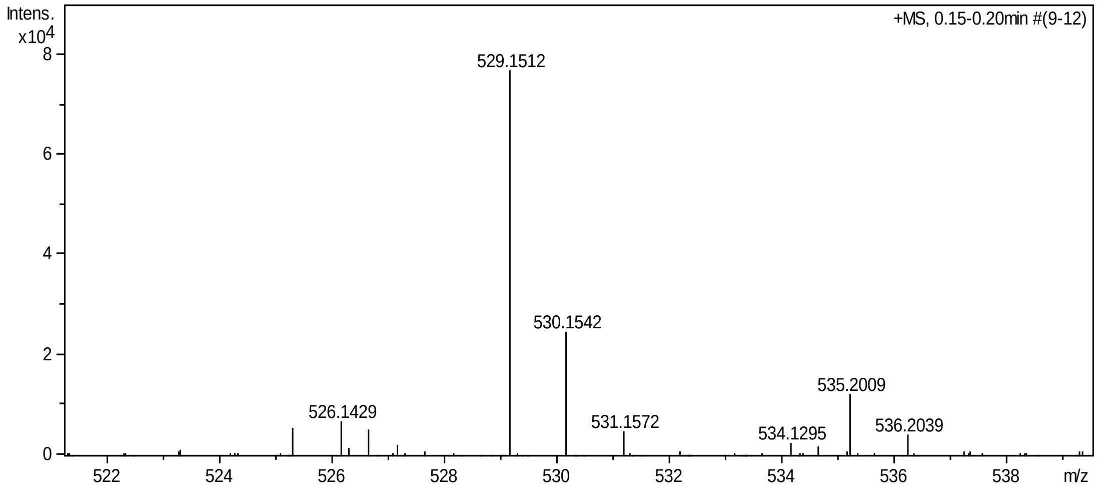
Intens.
x10^4
+MS, 0.15-0.20min #(9-12)
529.1512
530.1542
535.2009
526.1429
531.1572
534.1295
536.2039
0
2
4
6
8
522
524
526
528
530
532
534
536
538
m/z

5-Benzoyl-4-(4-chlorophenyl)-2,6-dimethylpyrrolo[3,4-*c*]carbazole-1,3(2*H*,6*H*)-dione (4b): green solid, 425 mg, 89%, m.p. 231-233 ºC; ^{1}H NMR (400 MHz, $CDCl_3$) δ: 9.23 (d, *J* = 8.0 Hz, 1H, ArH), 7.66-7.62 (m, 1H, ArH), 7.57 (d, *J* = 7.6 Hz, 2H, ArH), 7.49 (d, *J* = 7.6 Hz, 1H, ArH), 7.45-7.39 (m, 2H, ArH), 7.32 (t, *J* = 8.0 Hz, 2H, ArH), 7.20-7.04 (m, 4H, ArH), 3.59 (s, 3H, CH_3), 3.18 (s, 3H, CH_3); ^{13}C {^{1}H} NMR (100 MHz, $CDCl_3$) δ: 196.8, 168.4, 168.2, 143.6, 141.3, 138.1, 134.1, 134.0, 133.6, 132.8, 131.6, 131.7, 131.6, 131.5, 131.4, 131.3, 129.4, 128.9, 128.7, 127.0, 126.7, 126.2, 121.4, 120.6, 120.1, 119.9, 109.0, 32.3, 23.8; IR (KBr) υ: 2967, 1786, 1714, 1612, 1488, 1456, 1358, 1312, 1188, 1025, 988, 767, 756 cm^{-1}; MS (*m/z*): HRMS (ESI-TOF) Calcd. for $C_{29}H_{19}ClNaN_2O_3$ ($[M+Na]^+$): 501.0976, Found: 501.0963.

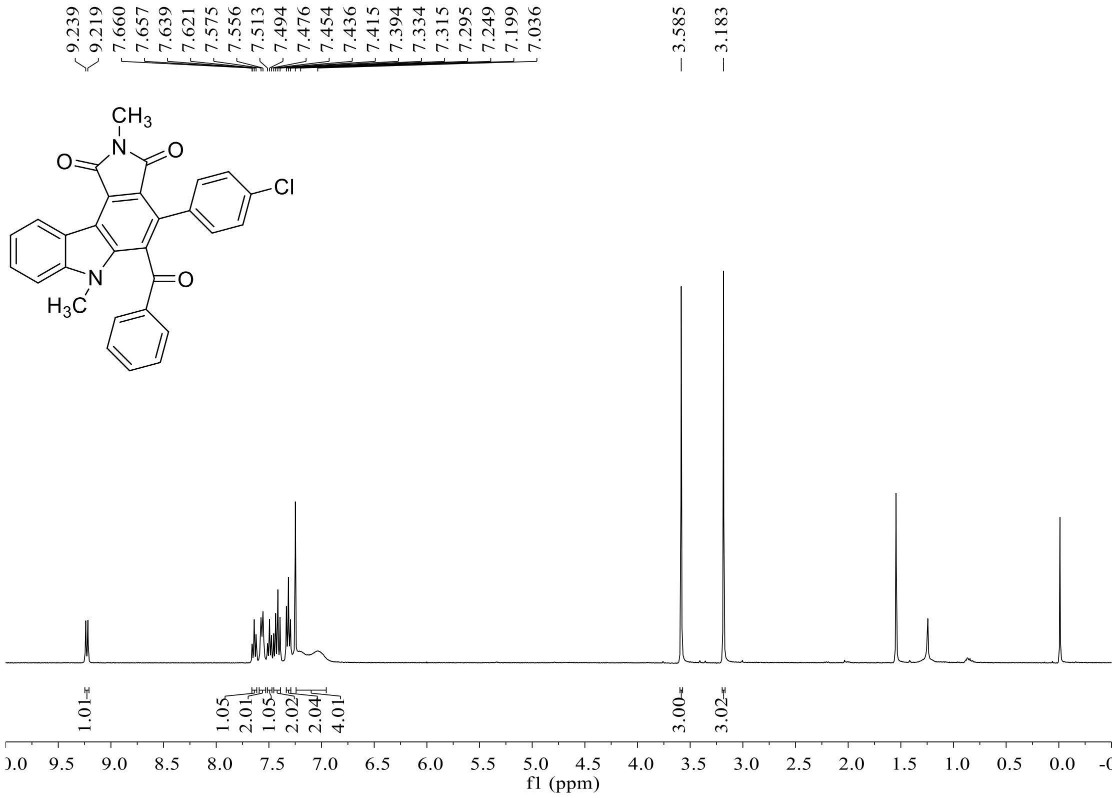

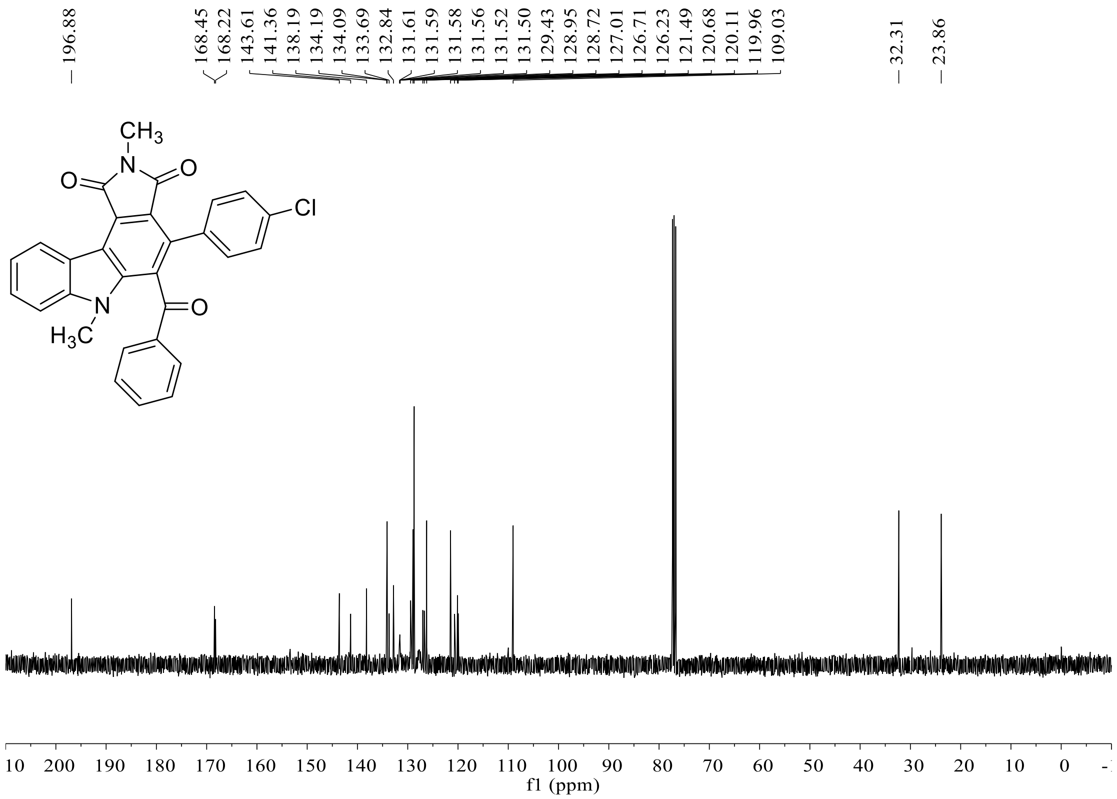
196.88
168.45
168.22
143.61
141.36
138.19
134.19
134.09
133.69
132.84
131.61
131.59
131.58
131.56
131.52
131.50
129.43
128.95
128.72
127.01
126.71
126.23
121.49
120.68
120.11
119.96
109.03
32.31
23.86
CH3
N
O
O
Cl
N
H3C
O
f1 (ppm)

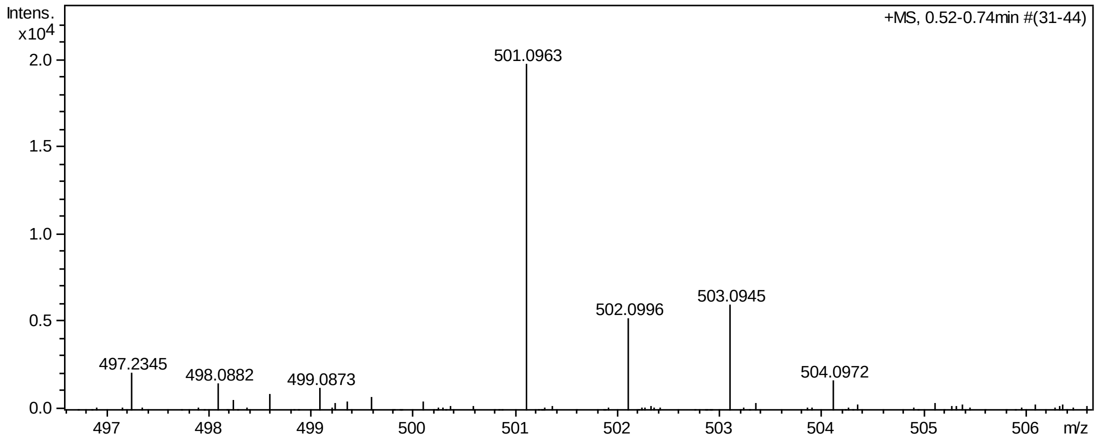
Intens.
x10^4
+MS, 0.52-0.74min #(31-44)
501.0963
502.0996
503.0945
497.2345
498.0882
499.0873
504.0972
m/z

2,6-Dimethyl-5-(4-methylbenzoyl)-4-phenylpyrrolo[3,4-*c*]carbazole-1,3(2*H*,6*H*)-dione (4c): green solid, 421 mg, 92%, m.p. 225-227 °C; ^{1}H NMR (400 MHz, $CDCl_3$) δ: 9.20 (d, *J* = 8.0 Hz, 1H, ArH), 7.64-7.60 (m, 1H, ArH), 7.49 (d, *J* = 7.6 Hz, 2H, ArH), 7.41 (t, *J* = 8.0 Hz, 1H, ArH), 7.38 (d, *J* = 8.4 Hz, 1H, ArH), 7.28-7.26 (m, 2H, ArH), 7.12 (d, *J* = 8.4 Hz, 2H, ArH), 7.07-7.03 (m, 2H, ArH), 3.57 (s, 3H, CH_3), 3.18 (s, 3H, CH_3); ^{13}C {^{1}H} NMR (100 MHz, $CDCl_3$) δ: 196.2, 168.4, 168.2, 145.3, 143.5, 141.2, 135.8, 134.1, 133.6, 132.9, 129.4, 128.8, 126.9, 126.8, 126.1, 121.3, 120.5, 120.1, 119.9, 108.9, 32.1, 23.8, 21.7; IR (KBr) υ: 2943, 1767, 1732, 1611, 1485, 1467, 1358, 1323, 1185, 1032, 981, 786, 732 cm^{-1}; MS (*m/z*): HRMS (ESI-TOF) Calcd. for $C_{30}H_{21}NaN_2O_3$ ($[M+Na]^+$): 515.1133, Found: 515.1134.

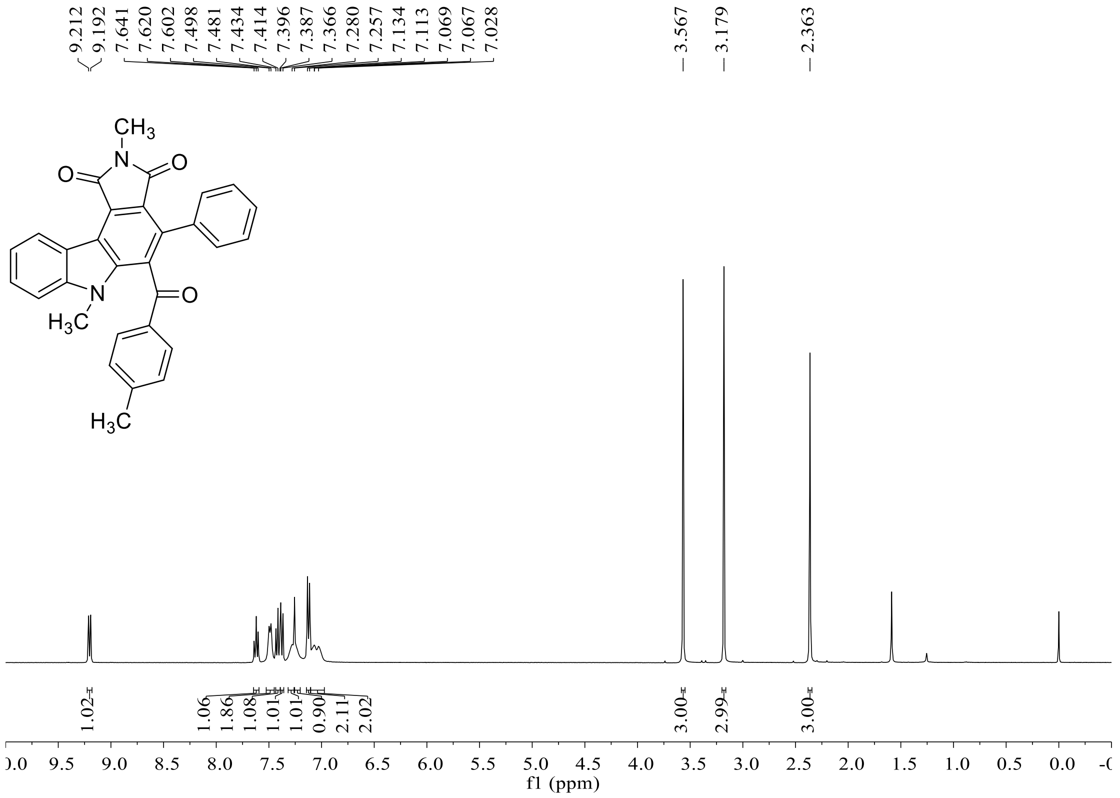

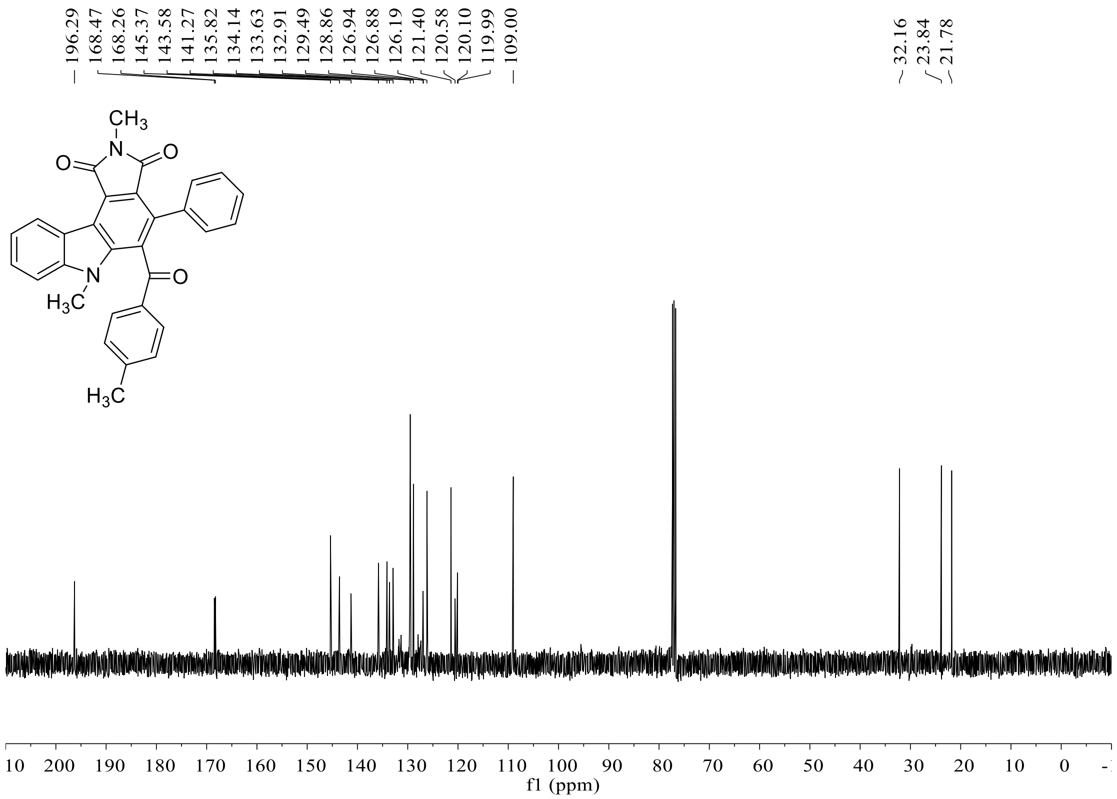
196.29
168.47
168.26
145.37
143.58
141.27
135.82
134.14
133.63
132.91
129.49
128.86
126.94
126.88
126.19
121.40
120.58
120.10
119.99
109.00
32.16
23.84
21.78
f1 (ppm)

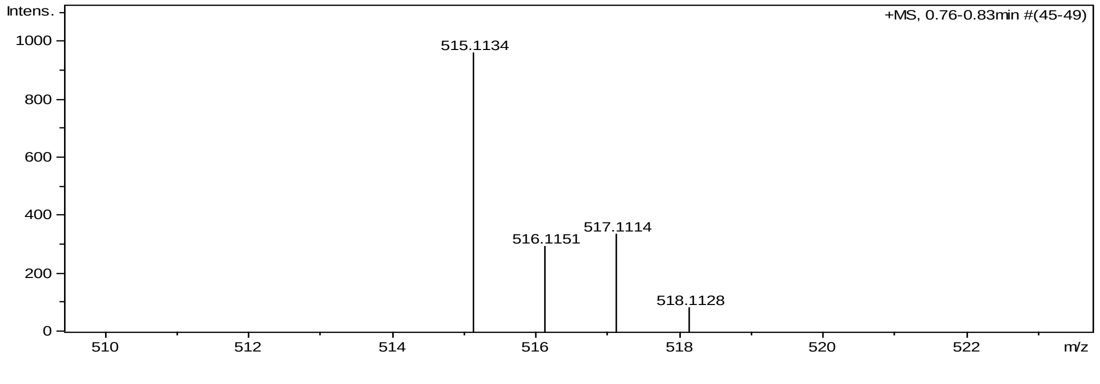
Intens.
+MS, 0.76-0.83min #(45-49)
515.1134
516.1151
517.1114
518.1128
m/z

2,6-Dimethyl-5-(4-methylbenzoyl)-4-(*p*-tolyl)pyrrolo[3,4-*c*]carbazole-1,3(2*H*,6*H*)-dione (4d): green solid, 430 mg, 91%, m.p. 216-218 °C; ^{1}H NMR (400 MHz, $CDCl_3$) δ: 9.23 (d, *J* = 8.0 Hz, 1H, ArH), 7.64-7.60 (m, 1H, ArH), 7.50 (d, *J* = 7.6 Hz, 2H, ArH), 7.42 (t, *J* = 8.0 Hz, 1H, ArH), 7.18-7.09 (m, 4H, ArH), 6.93 (d, *J* = 11.6 Hz, 2H, ArH), 3.57 (s, 3H, CH_3), 3.18 (s, 3H, CH_3), 2.35 (s, 3H, CH_3), 2.28 (s, 3H, CH_3); ^{13}C {^{1}H} NMR (100 MHz, $CDCl_3$) δ: 196.5, 168.6, 168.3, 144.9, 143.5, 141.3, 137.5, 136.0, 135.3, 131.3, 129.6, 129.3, 128.6, 127.0, 126.9, 126.1, 121.2, 120.4, 120.3, 120.2, 108.9, 32.1, 23.7, 21.7, 21.3; IR (KBr) υ: 2965, 1763, 1732, 1611, 1485, 1458, 1376, 1311, 1185, 1021, 968, 786, 754 cm^{-1}; MS (*m*/*z*): HRMS (ESI-TOF) Calcd. for $C_{31}H_{24}NaN_2O_3$ ([M+Na]$^+$): 495.1679, Found: 495.1668.

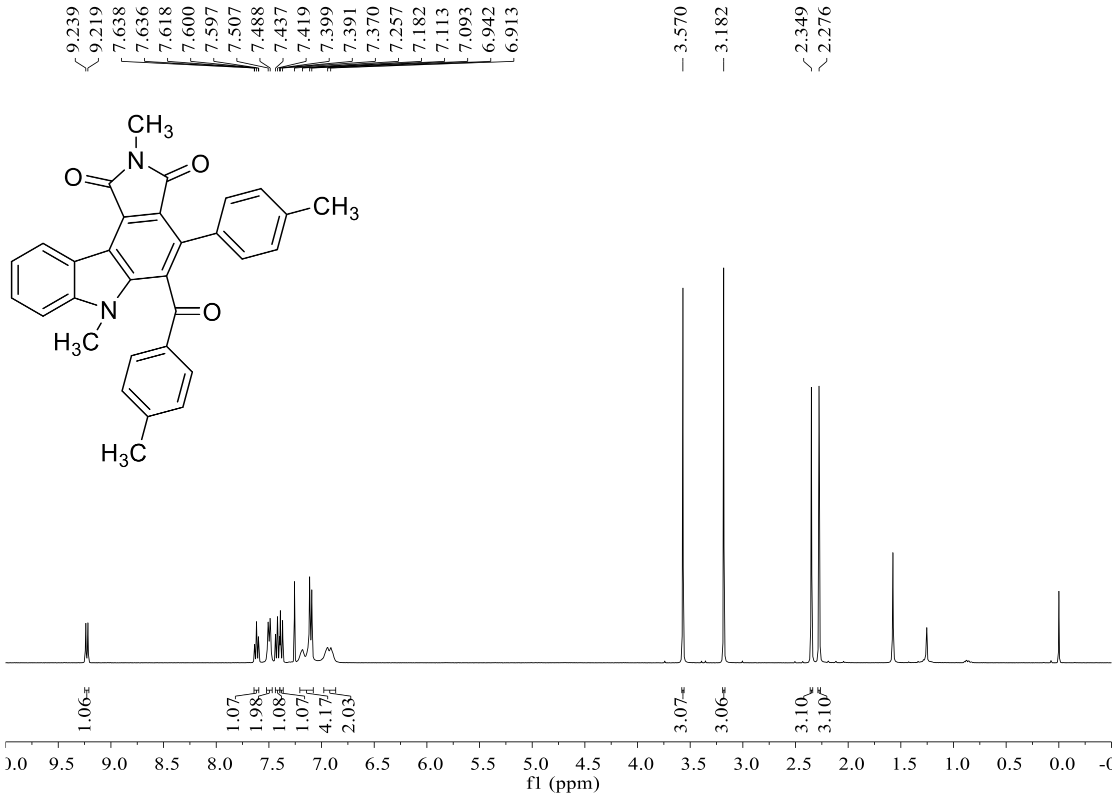

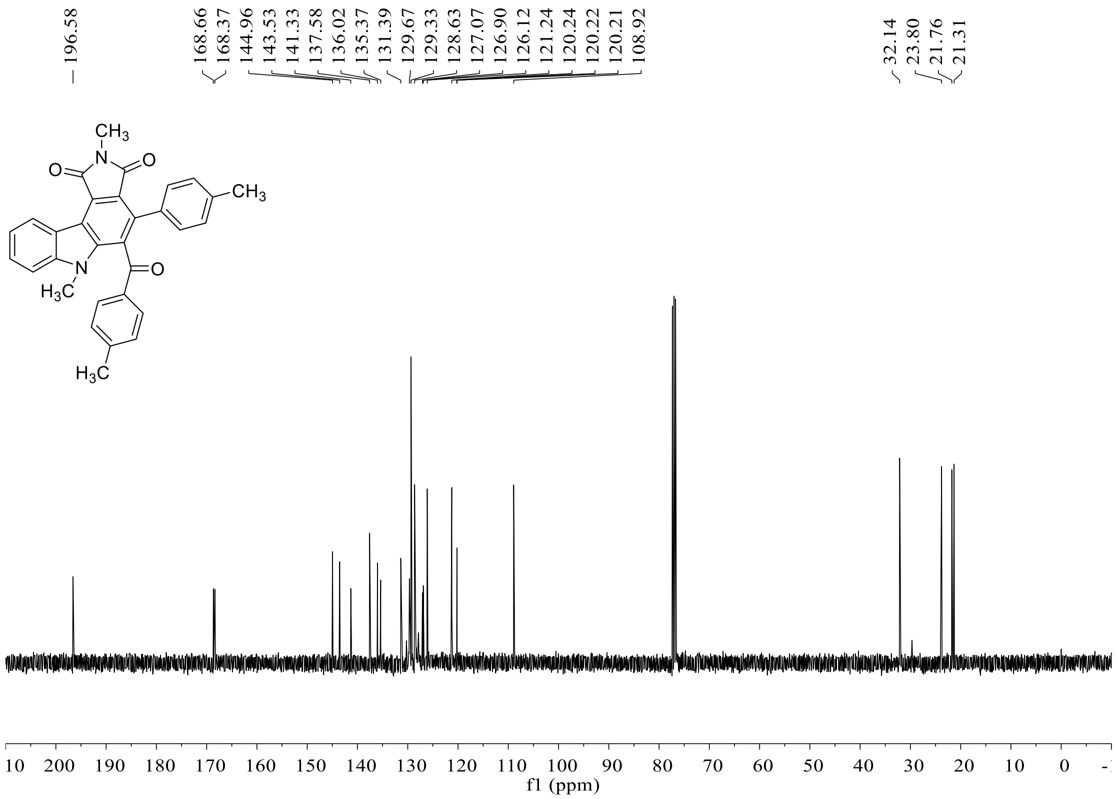
196.58
168.66
168.37
144.96
143.53
141.33
137.58
136.02
135.37
131.39
129.67
129.33
128.63
127.07
126.90
126.12
121.24
120.24
120.22
120.21
108.92
32.14
23.80
21.76
21.31
CH3
O
N
O
CH3
N
H3C
O
H3C
f1 (ppm)

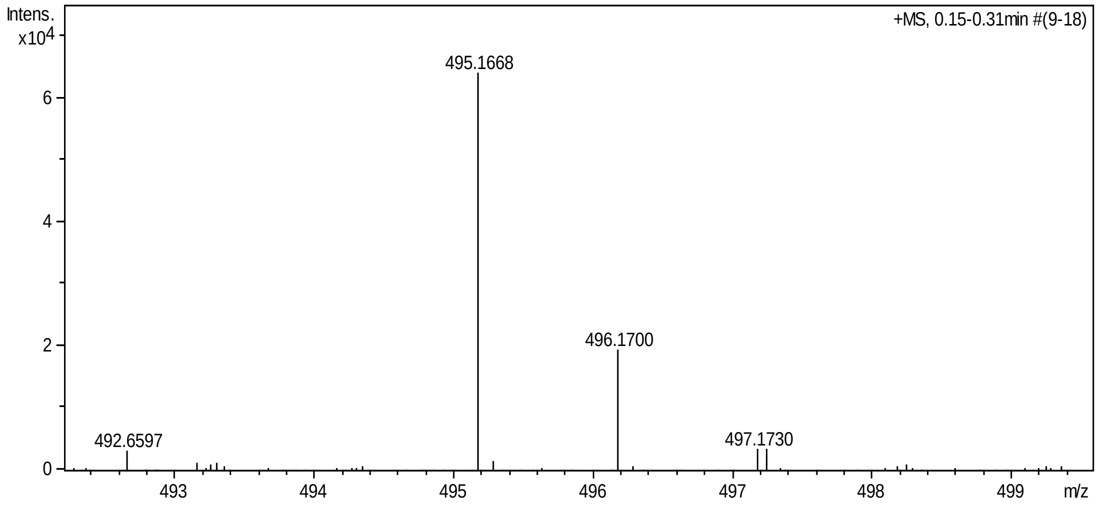
Intens.
x10^4
+MS, 0.15-0.31min #(9-18)
495.1668
496.1700
492.6597
497.1730
m/z

5-Acetyl-6-methyl-2,4-diphenylpyrrolo[3,4-*c*]carbazole-1,3(2*H*,6*H*)-dione (4e): green solid, 377 mg, 85%, m.p. 239-241 °C; ^{1}H NMR (400 MHz, $CDCl_3$) δ: 9.24 (d, J = 7.6 Hz, 1H, ArH), 7.68-7.64 (m, 1H, ArH), 7.49-7.46 (m, 8H, ArH), 7.44-7.40 (m, 3H, ArH), 7.38-7.34 (m, 1H, ArH), 3.76 (s, 3H, CH_3), 2.16 (s, 3H, CH_3); ^{13}C {^{1}H} NMR (100 MHz, $CDCl_3$) δ: 204.7, 167.3, 166.9, 143.6, 140.2, 134.4, 134.0, 131.9, 130.2, 130.1, 128.9, 128.8, 128.6, 128.1, 127.7, 126.7, 126.3, 126.2, 121.4, 121.0, 120.0, 119.1, 109.0, 33.8, 32.4; IR (KBr) υ: 2876, 1863, 1732, 1632, 1488, 1468, 1367, 1321, 1188, 1021, 988, 789, 743 cm^{-1}; MS (m/z): HRMS (ESI-TOF) Calcd. for $C_{29}H_{20}NaN_2O_3$ ($[M+Na]^+$): 467.1366, Found: 467.1366.

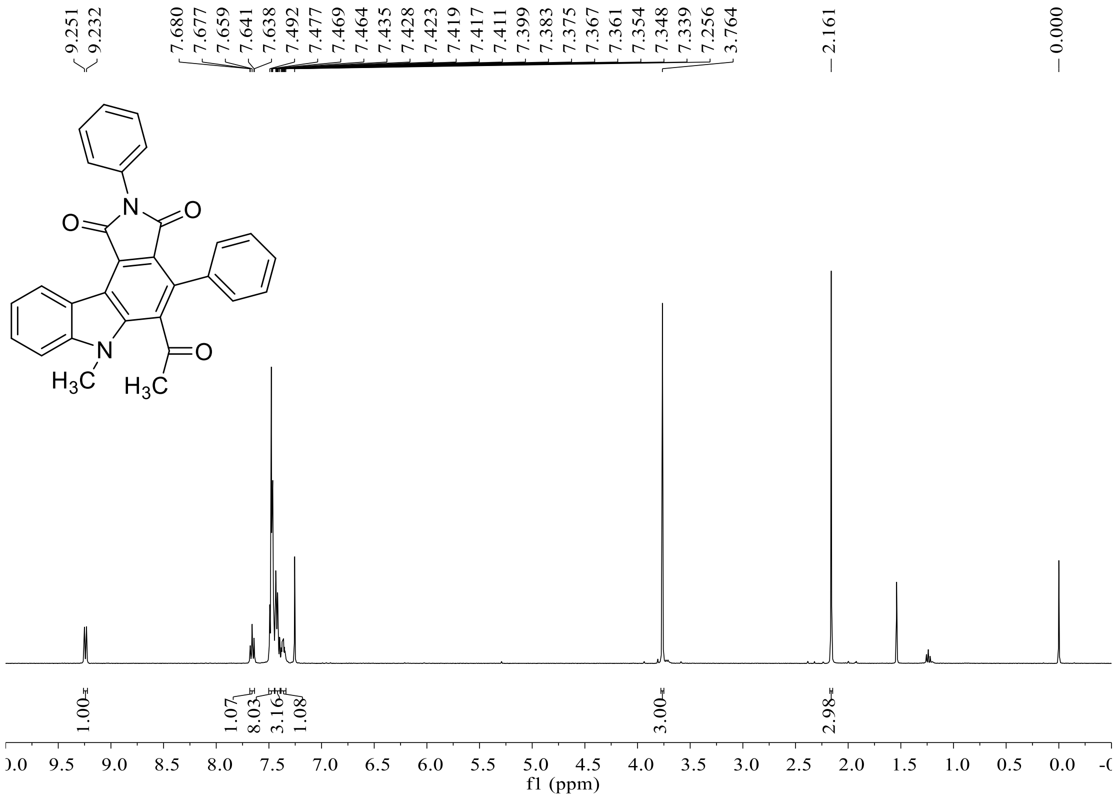

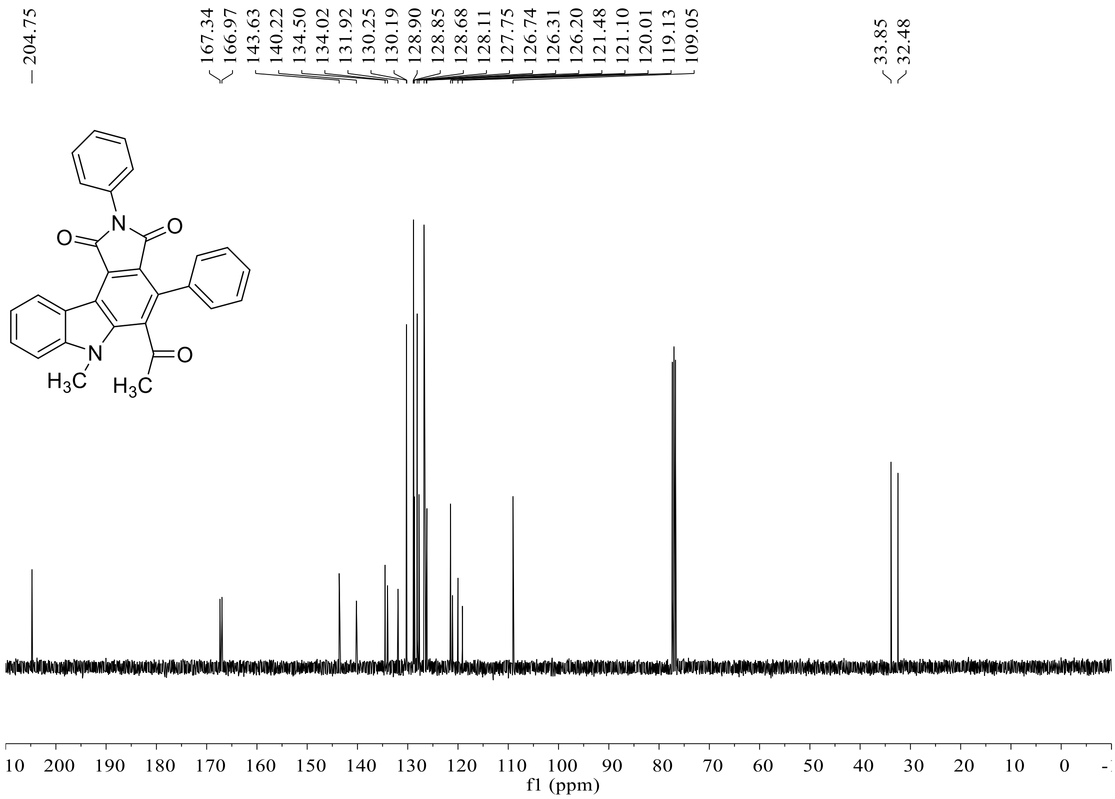
204.75
167.34
166.97
143.63
140.22
134.50
134.02
131.92
130.25
130.19
128.90
128.85
128.68
128.11
127.75
126.74
126.31
126.20
121.48
121.10
120.01
119.13
109.05
33.85
32.48
O
N
O
N
O
H3C
H3C
f1 (ppm)

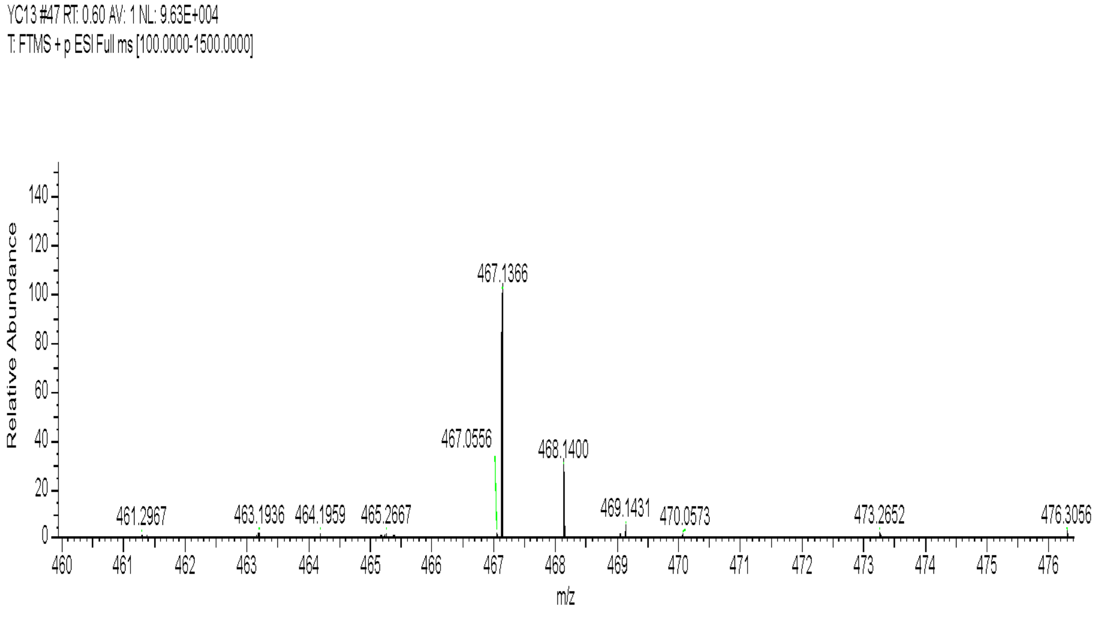
YC13 #47 RT: 0.60 AV: 1 NL: 9.63E+004
T: FTMS + p ESI Full ms [100.0000-1500.0000]
467.1366
467.0556
468.1400
461.2967
463.1936
464.1959
465.2667
469.1431
470.0573
473.2652
476.3056
Relative Abundance
m/z

5-Benzoyl-6-methyl-2,4-diphenylpyrrolo[3,4-*c*]carbazole-1,3(2*H*,6*H*)-dione (4f): green solid, 425 mg, 84%, m.p. 218-220 °C; ^{1}H NMR (400 MHz, $CDCl_3$) δ: 9.29 (d, *J* = 8.0 Hz, 1H, ArH), 7.65 (t, *J* = 8.0 Hz, 1H, ArH), 7.60 (d, *J* = 7.2 Hz, 2H, ArH), 7.51-7.42 (m, 8H, ArH), 7.38-7.34 (m, 4H, ArH), 7.30 (t, *J* = 8.0 Hz, 3H, ArH), 7.20-7.08 (m, 3H, ArH), 3.63 (s, 3H, CH_3); ^{13}C {^{1}H} NMR (100 MHz, $CDCl_3$) δ: 196.9, 167.5, 167.0, 143.6, 141.6, 138.3, 135.6, 134.2, 133.8, 131.9, 130.3, 129.4, 128.9, 128.8, 128.5, 127.9, 127.7, 127.4, 126.7, 126.6, 126.3, 121.4, 120.6, 120.1, 119.4, 109.1, 32.3; IR (KBr) υ: 1831, 1725, 1611, 1495, 1458, 1375, 1321, 1185, 1021, 986, 793, 732 cm^{-1}; MS (*m*/*z*): HRMS (ESI-TOF) Calcd. for $C_{43}H_{33}NaN_3O_4$ ($[M+Na]^+$): 529.1523, Found: 529.1507.

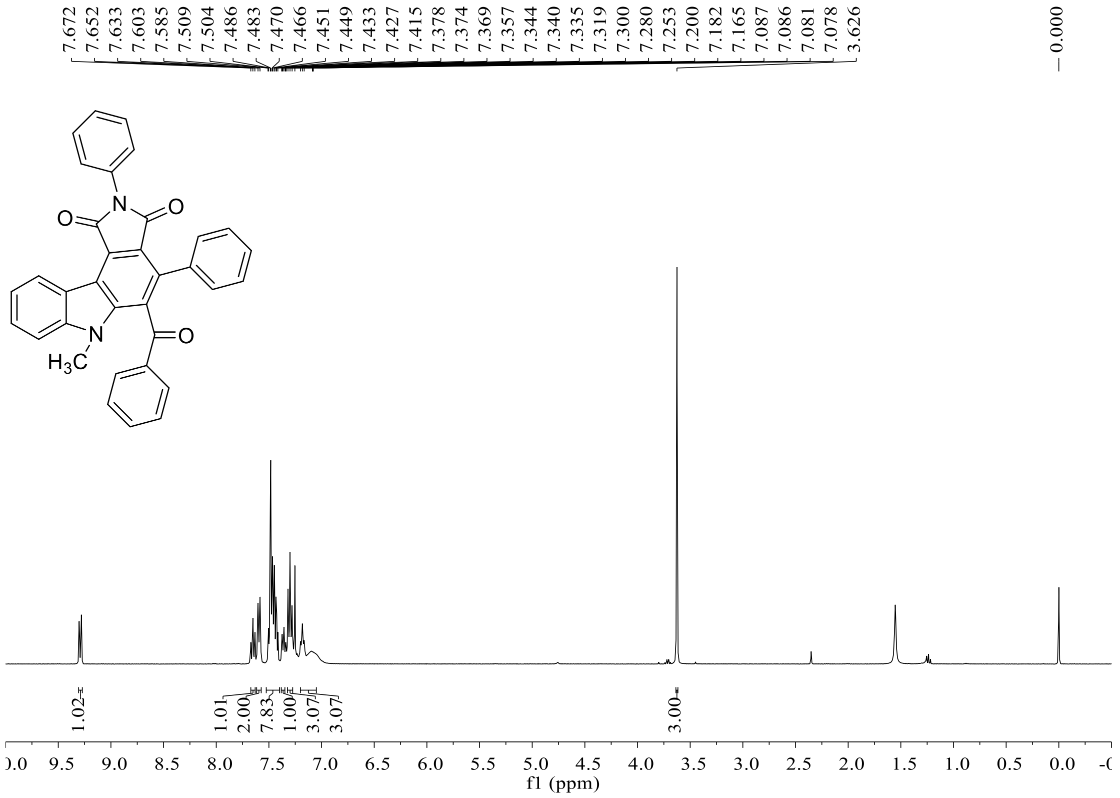

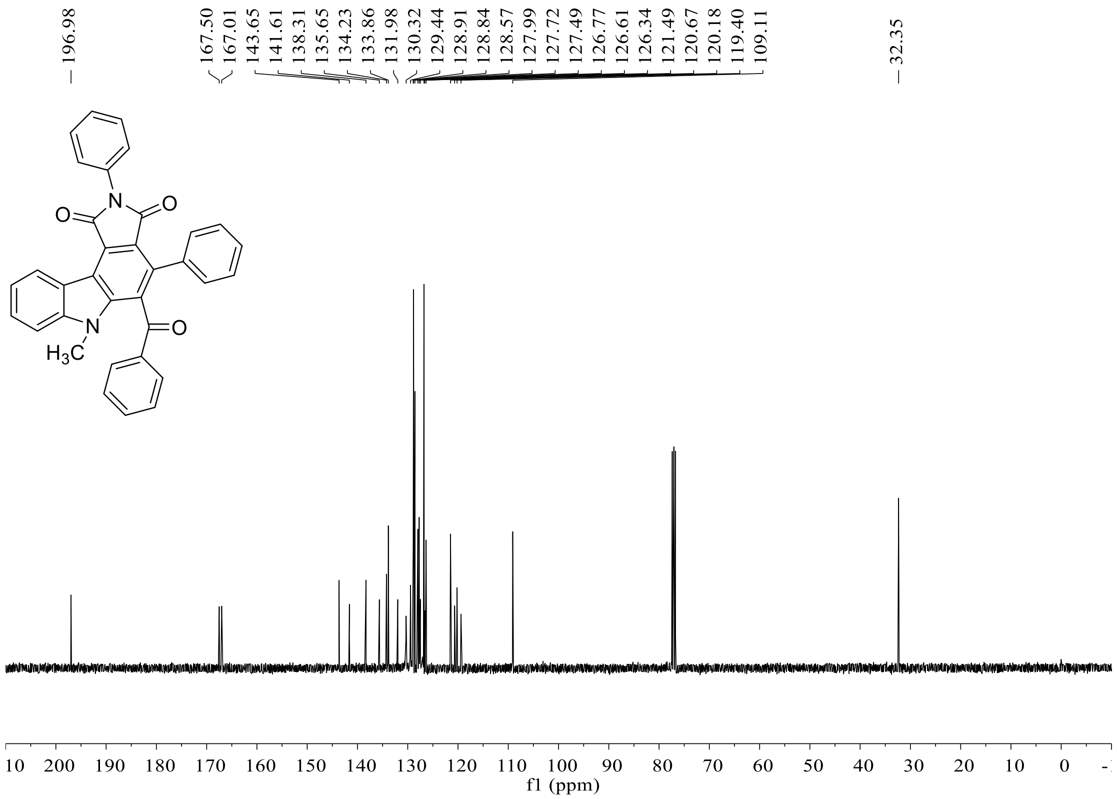
196.98
167.50
167.01
143.65
141.61
138.31
135.65
134.23
133.86
131.98
130.32
129.44
128.91
128.84
128.57
127.99
127.72
127.49
126.77
126.61
126.34
121.49
120.67
120.18
119.40
109.11
32.35
O
N
O
N
H3C
O
f1 (ppm)

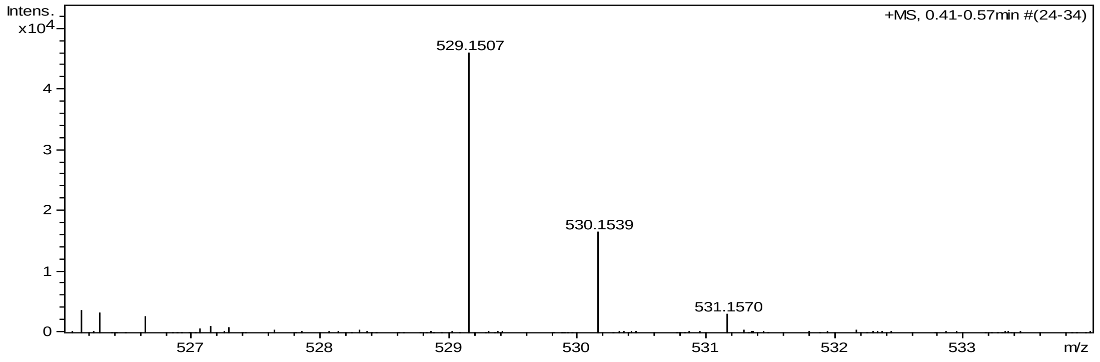
Intens.
x10^4
+MS, 0.41-0.57min #(24-34)
529.1507
530.1539
531.1570
m/z

5-Benzoyl-6-methyl-2-phenyl-4-(*p*-tolyl)pyrrolo[3,4-*c*]carbazole-1,3(2*H*,6*H*)-dione (4g): green solid, 483 mg, 93%, m.p. 225-227 °C; ^{1}H NMR (400 MHz, $CDCl_3$) δ: 9.28 (d, *J* = 8.0 Hz, 1H, ArH), 7.66-7.60 (m, 3H, ArH), 7.51-7.48 (m, 3H, ArH), 7.46-7.40 (m, 4H, ArH), 7.37-7.35 (m, 1H, ArH), 7.31 (t, *J* = 8.0 Hz, 2H, ArH), 7.19-6.87 (m, 4H, ArH), 3.61 (s, 3H, CH_3), 2.24 (s, 3H, CH_3); ^{13}C {^{1}H} NMR (100 MHz, $CDCl_3$) δ: 197.0, 167.5, 167.0, 143.5, 141.5, 138.3, 137.6, 135.8, 133.8, 132.0, 131.2, 129.4, 128.8, 128.5, 127.6, 127.5, 126.7, 126.6, 126.2, 121.4, 120.4, 120.1, 119.4, 109.1, 32.2, 21.3; IR (KBr) υ: 2934, 1756, 1716, 1611, 1485, 1456, 1375, 1311, 1185, 1023, 974, 786, 747 cm^{-1}; MS (*m*/*z*): HRMS (ESI-TOF) Calcd. for $C_{35}H_{25}N_2O_3$ ([M+H]$^+$): 521.1860, Found: 521.1863.

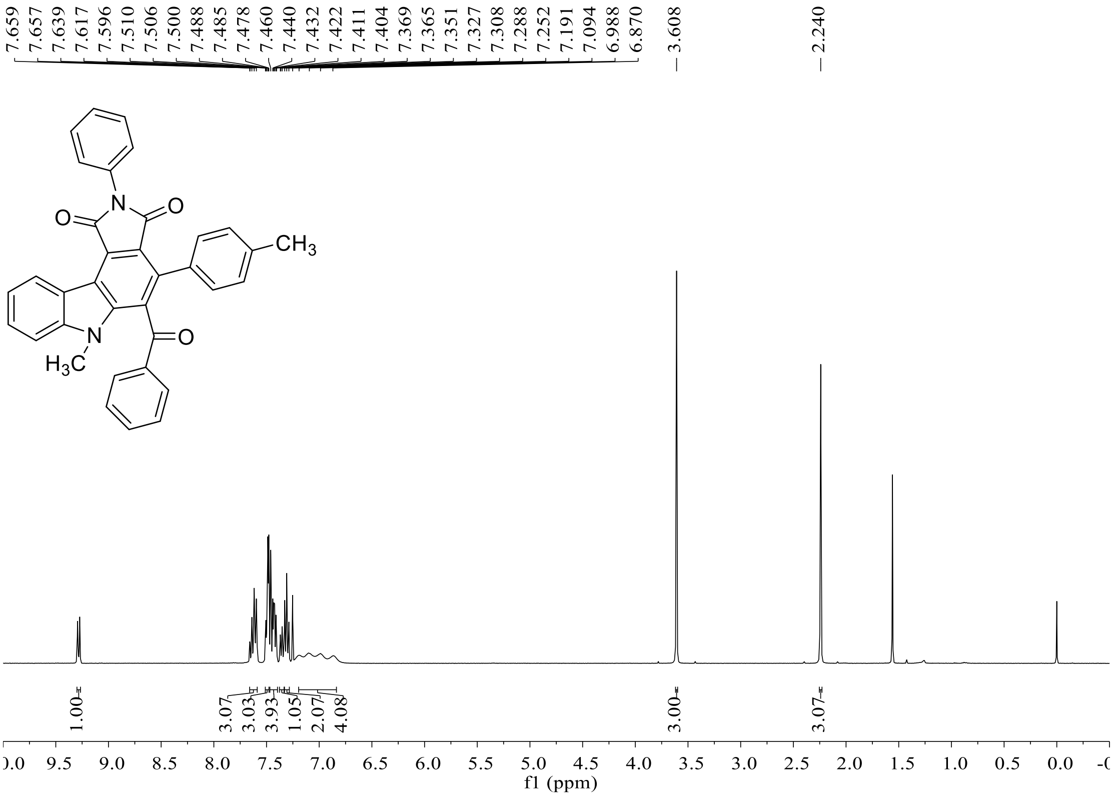

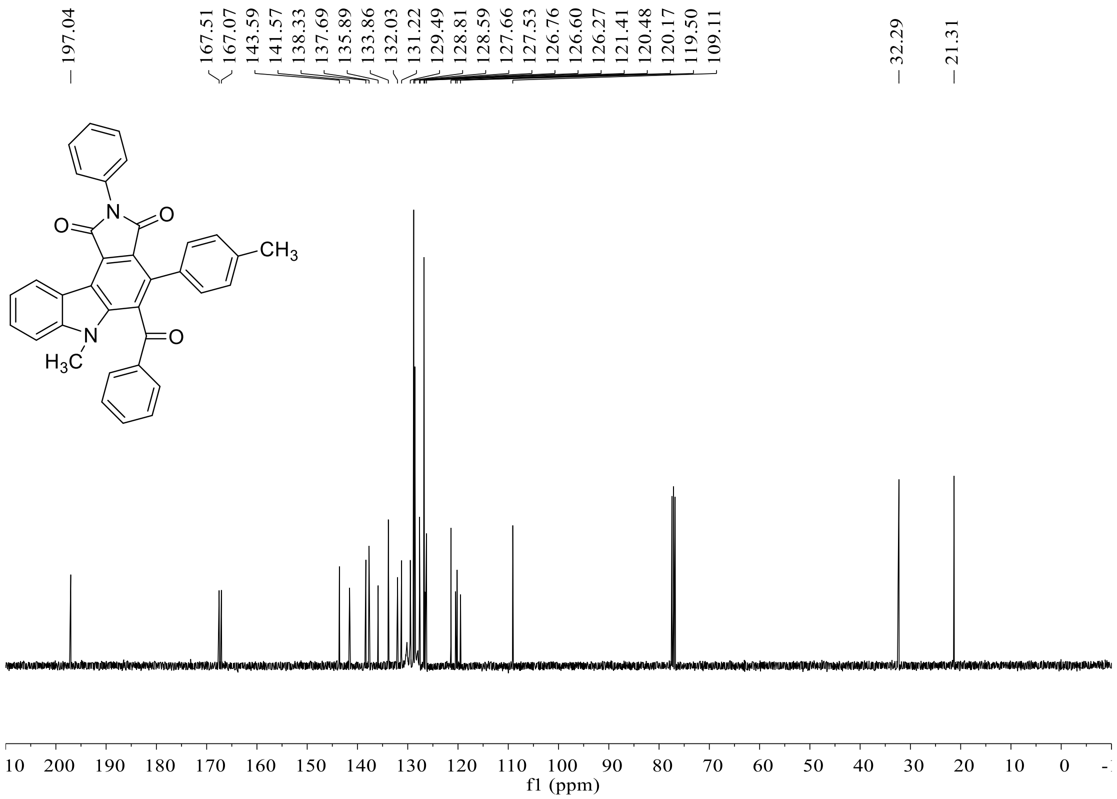
197.04
167.51
167.07
143.59
141.57
138.33
137.69
135.89
133.86
132.03
131.22
129.49
128.81
128.59
127.66
127.53
126.76
126.60
126.27
121.41
120.48
120.17
119.50
109.11
32.29
21.31
O
N
O
CH3
N
H3C
O
f1 (ppm)

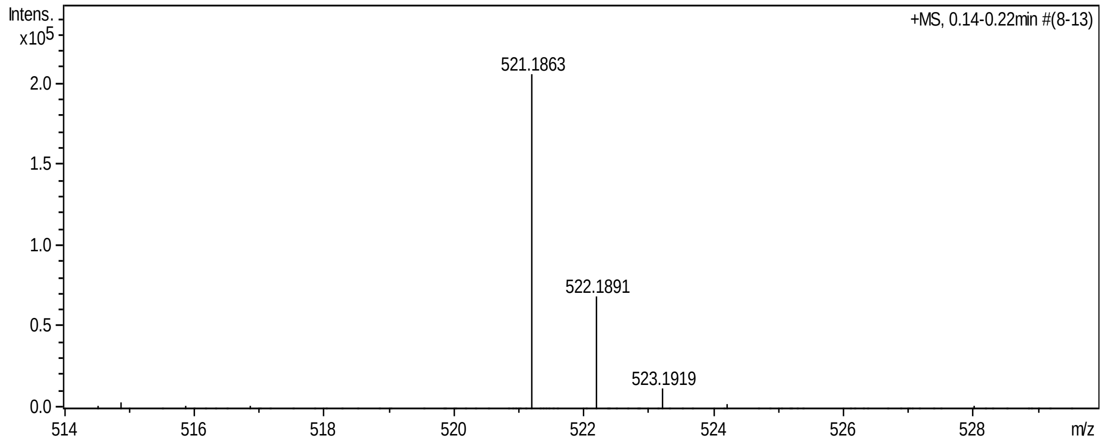
Intens.
x10^5
+MS, 0.14-0.22min #(8-13)
521.1863
522.1891
523.1919
m/z

6-Methyl-5-(4-methylbenzoyl)-4-(3-nitrophenyl)-2-phenylpyrrolo[3,4-*c*]carbazole-1,3(2*H*,6*H*)-dione (4h): yellow solid, 429 mg, 76%, m.p. 228-230 °C; ^{1}H NMR (400 MHz, $CDCl_3$) δ: 9.29 (d, *J* = 8.0 Hz, 1H, ArH), 8.23-7.98 (m, 2H, ArH), 7.70-7.66 (m, 2H, ArH), 7.51-7.45 (m, 9H, ArH), 7.40-7.36 (m, 1H, ArH), 7.13 (t, *J* = 8.0 Hz, 2H, ArH), 3.64 (s, 3H, CH_3), 2.35 (s, 3H, CH_3); ^{13}C {^{1}H} NMR (100 MHz, $CDCl_3$) δ: 167.2, 166.9, 143.7, 141.4, 136.0, 135.6, 132.2, 131.7, 129.7, 129.6, 129.5, 129.5, 129.3, 128.9, 127.9, 126.6, 126.4, 126.4, 122.9, 121.7, 121.2, 120.0, 119.3, 109.2, 32.3, 21.7; IR (KBr) υ: 2968, 1785, 1732, 1627, 1485, 1476, 1358, 1321, 1184, 1026, 975, 785, 736 cm^{-1}; MS (*m*/*z*): HRMS (ESI-TOF) Calcd. for $C_{35}H_{23}NaN_3O_5$ ([M+Na]$^+$): 588.1530, Found: 588.1513.

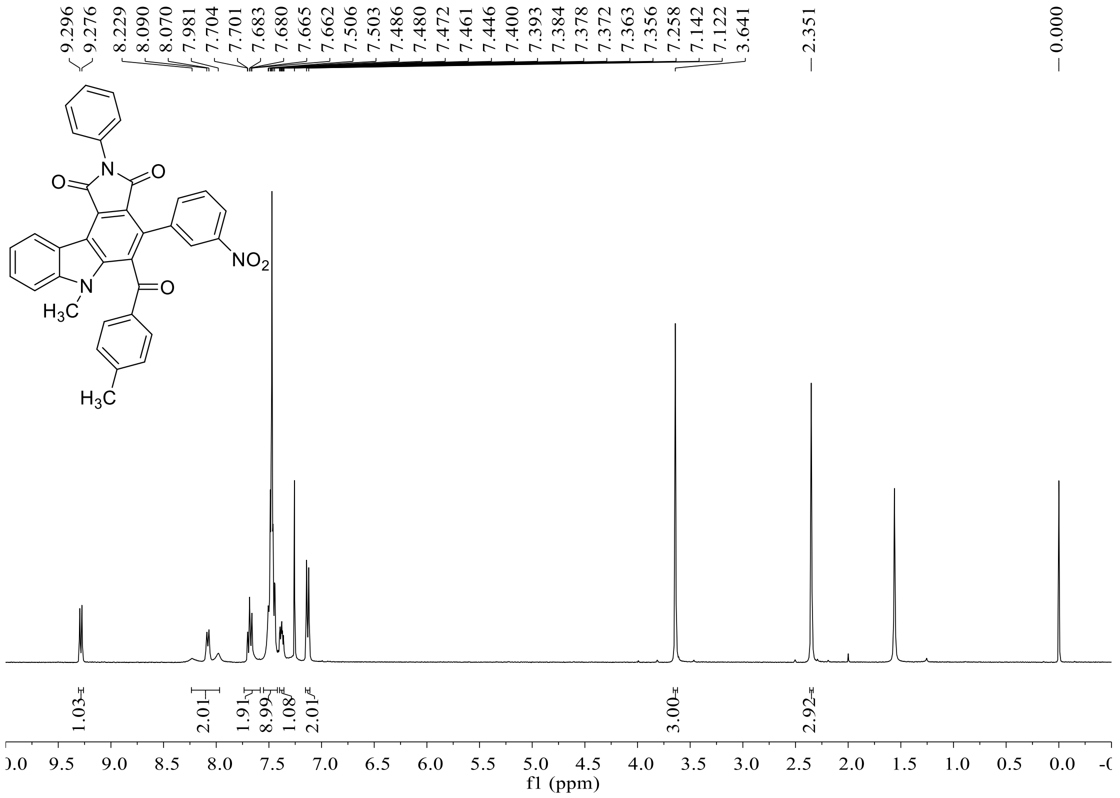

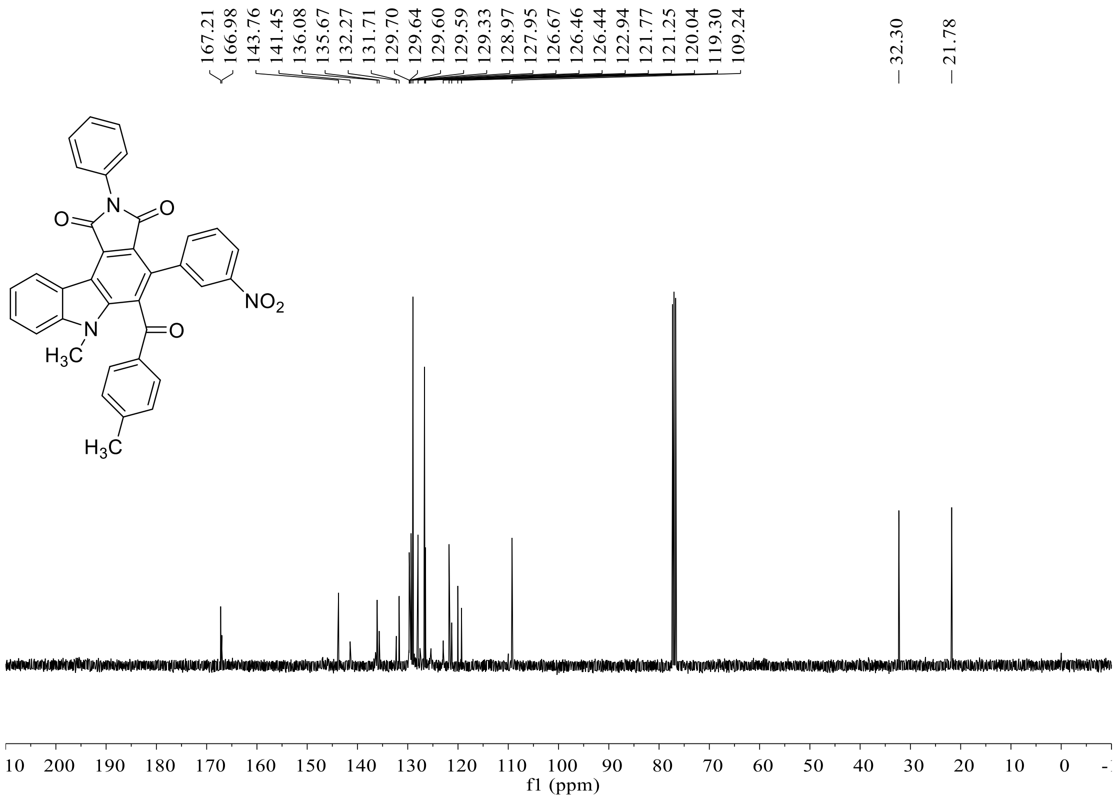

167.21
166.98
143.76
141.45
136.08
135.67
132.27
131.71
129.70
129.64
129.60
129.59
129.33
128.97
127.95
126.67
126.46
126.44
122.94
121.77
121.25
120.04
119.30
109.24
32.30
21.78
O
N
O
NO2
N
H3C
O
H3C
f1 (ppm)

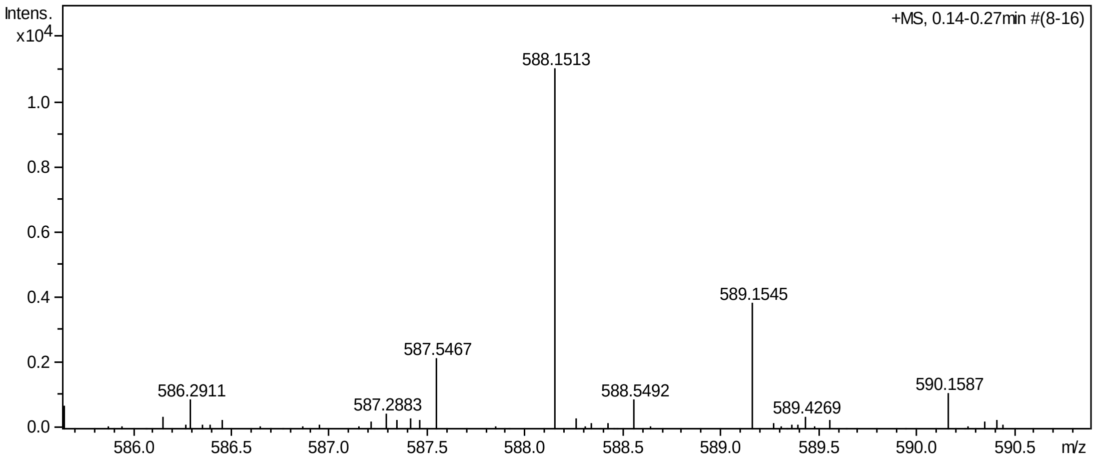

Intens.
x10^4
+MS, 0.14-0.27min #(8-16)
588.1513
589.1545
587.5467
586.2911
587.2883
588.5492
589.4269
590.1587
m/z

5-(4-Methoxybenzoyl)-6-methyl-2-phenyl-4-(*p*-tolyl)pyrrolo[3,4-*c*]carbazole-1,3(2*H*,6*H*)-dione (4i): yellow solid, 473 mg, 86%, m.p. 232-234 °C; ^{1}H NMR (400 MHz, $CDCl_3$) δ: 9.28 (d, *J* = 8.0 Hz, 1H, ArH), 7.64 (t, *J* = 8.0 Hz, 1H, ArH), 7.52-7.40 (m, 8H, ArH), 7.38-7.34 (m, 1H, ArH), 7.26 (s, 1H, ArH), 7.12-7.05 (m, 3H, ArH), 6.83 (d, *J* = 6.8 Hz, 1H, ArH), 6.63 (d, *J* = 7.2 Hz, 1H, ArH), 3.73 (s, 3H, OCH_3), 3.61 (s, 3H, OCH_3), 2.35 (s, 3H, CH_3); ^{13}C {^{1}H} NMR (100 MHz, $CDCl_3$) δ: 196.6, 167.5, 167.2, 159.1, 145.0, 143.6, 141.5, 135.8, 135.5, 131.9, 131.6, 131.4, 129.6, 129.6, 129.3, 128.8, 128.7, 127.9, 127.6, 126.7, 126.5, 126.3, 126.2, 121.3, 120.4, 120.1, 119.5, 113.1, 113.1, 112.7, 109.0, 55.0, 32.1, 21.7; IR (KBr) υ: 3056, 1832, 1754, 1611, 1524, 1474, 1368, 1311, 1185, 1023, 985, 787, 747 cm^{-1}; MS (*m*/*z*): HRMS (ESI-TOF) Calcd. for $C_{36}H_{26}NaN_2O_4$ ([M+Na]$^{+}$): 573.1785, Found: 573.1783.

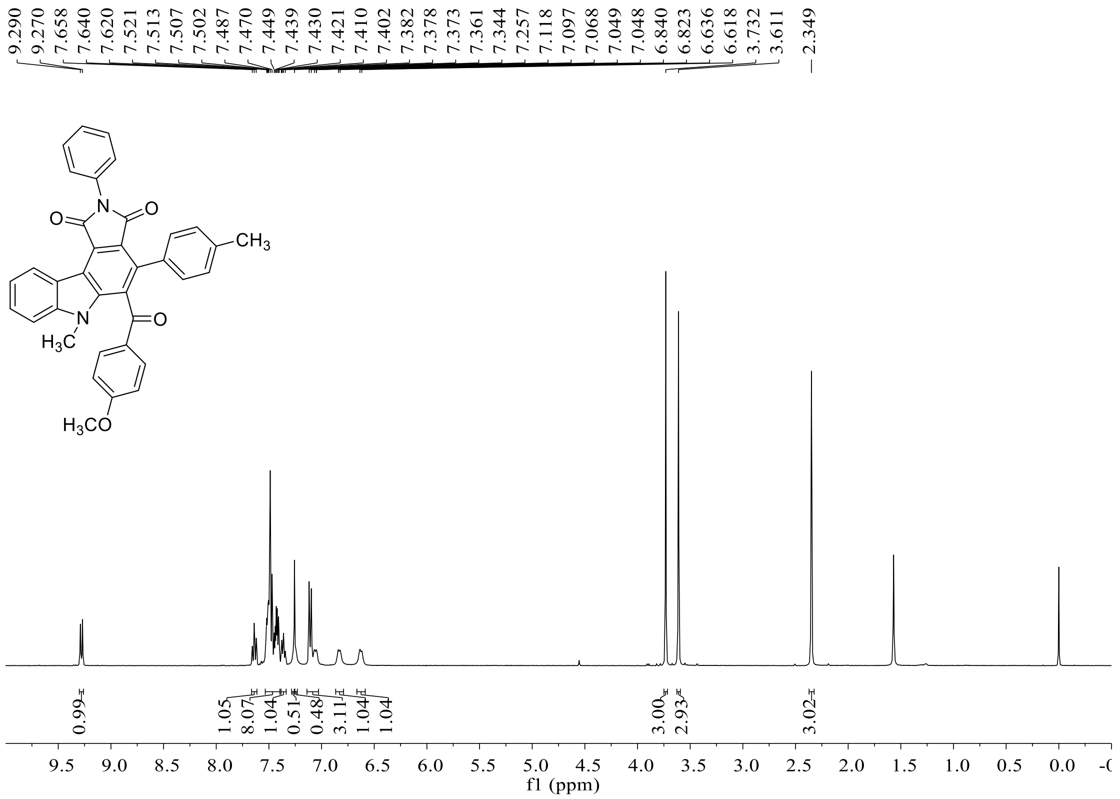

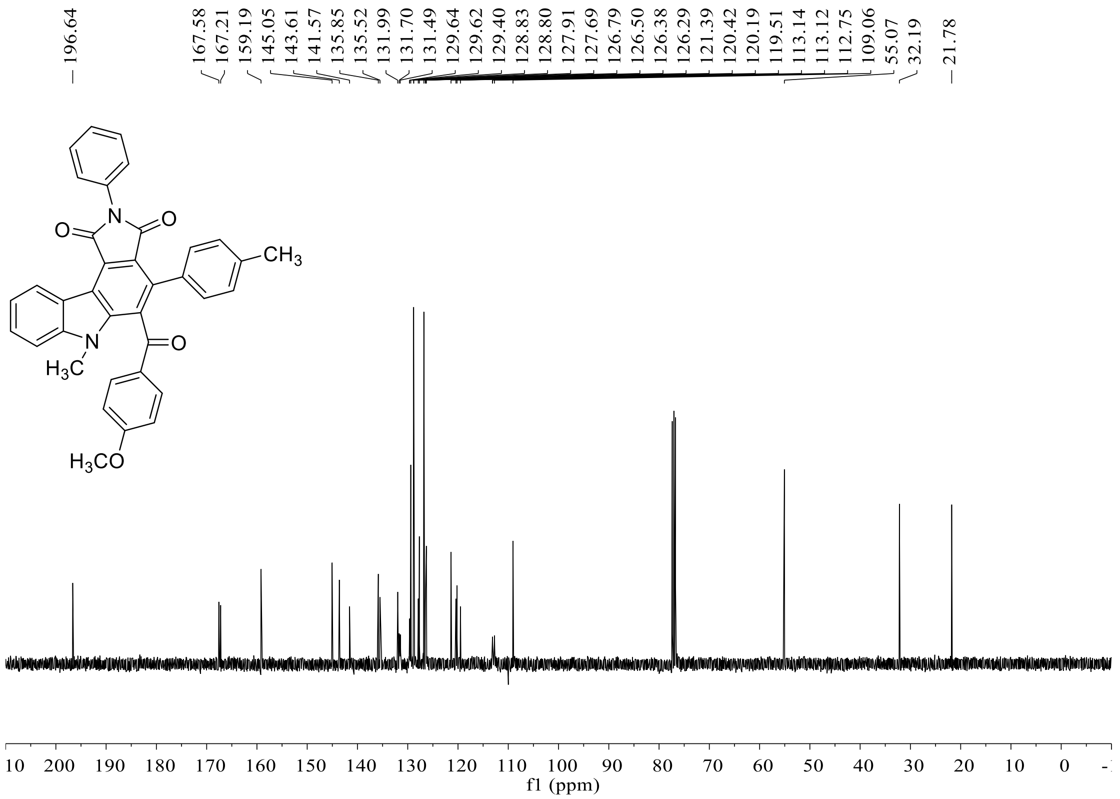
196.64
167.58
167.21
159.19
145.05
143.61
141.57
135.85
135.52
131.99
131.70
131.49
129.64
129.62
129.40
128.83
128.80
127.91
127.69
126.79
126.50
126.38
126.29
121.39
120.42
120.19
119.51
113.14
113.12
112.75
109.06
55.07
32.19
21.78
O
N
O
CH3
N
O
H3C
H3CO
10 200 190 180 170 160 150 140 130 120 110 100 90 80 70 60 50 40 30 20 10 0
f1 (ppm)

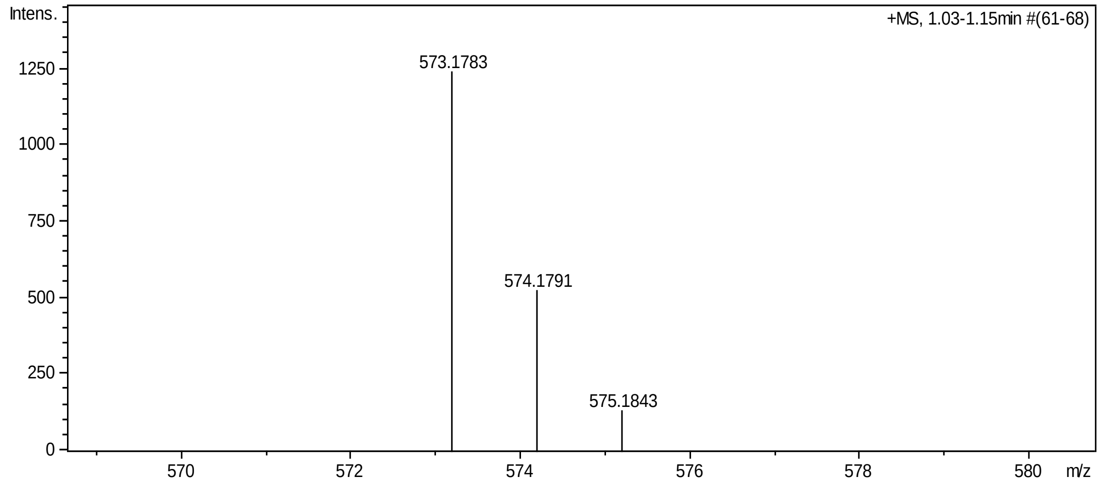
Intens.
+MS, 1.03-1.15min #(61-68)
1250
1000
750
500
250
0
573.1783
574.1791
575.1843
570
572
574
576
578
580
m/z

5-(3-Chlorobenzoyl)-6-methyl-2-phenyl-4-(*p*-tolyl)pyrrolo[3,4-*c*]carbazole-1,3(2*H*,6*H*)-dione (4j): green solid, 465 mg, 84%, m.p. 211-213 °C; ^{1}H NMR (400 MHz, $CDCl_3$) δ: 9.27 (d, *J* = 7.6 Hz, 1H, ArH), 7.65 (t, *J* = 8.0 Hz, 1H, ArH), 7.50-7.48 (m, 6H, ArH), 7.45-7.41 (m, 2H, ArH), 7.39-7.35 (m, 1H, ArH), 7.21-6.99 (m, 6H, ArH), 3.62 (s, 3H, CH_3), 2.36 (s, 3H, CH_3); ^{13}C {^{1}H} NMR (100 MHz, $CDCl_3$) δ: 167.3, 166.8, 143.6, 141.4, 136.0, 135.8, 133.7, 131.8, 129.4, 129.0, 128.9, 128.1, 127.8, 126.7, 126.4, 126.3, 121.5, 120.8, 120.1, 119.3, 109.1, 32.2, 21.7; IR (KBr) υ: 2932, 1832, 1723, 1621, 1588, 1456, 1354, 1321, 1186, 1023, 956, 789, 768 cm^{-1}; MS (*m*/*z*): HRMS (ESI-TOF) Calcd. for $C_{35}H_{23}ClNaN_2O_3$ ([M+Na]$^+$): 577.1289, Found: 577.1271.

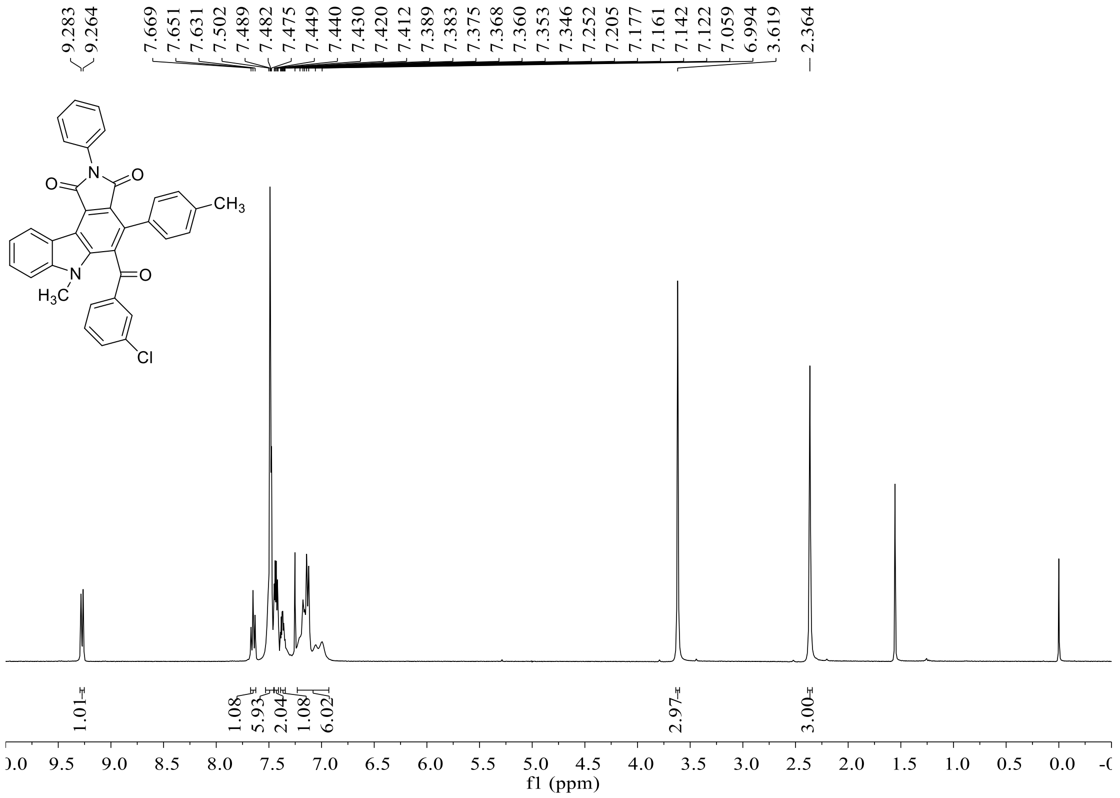

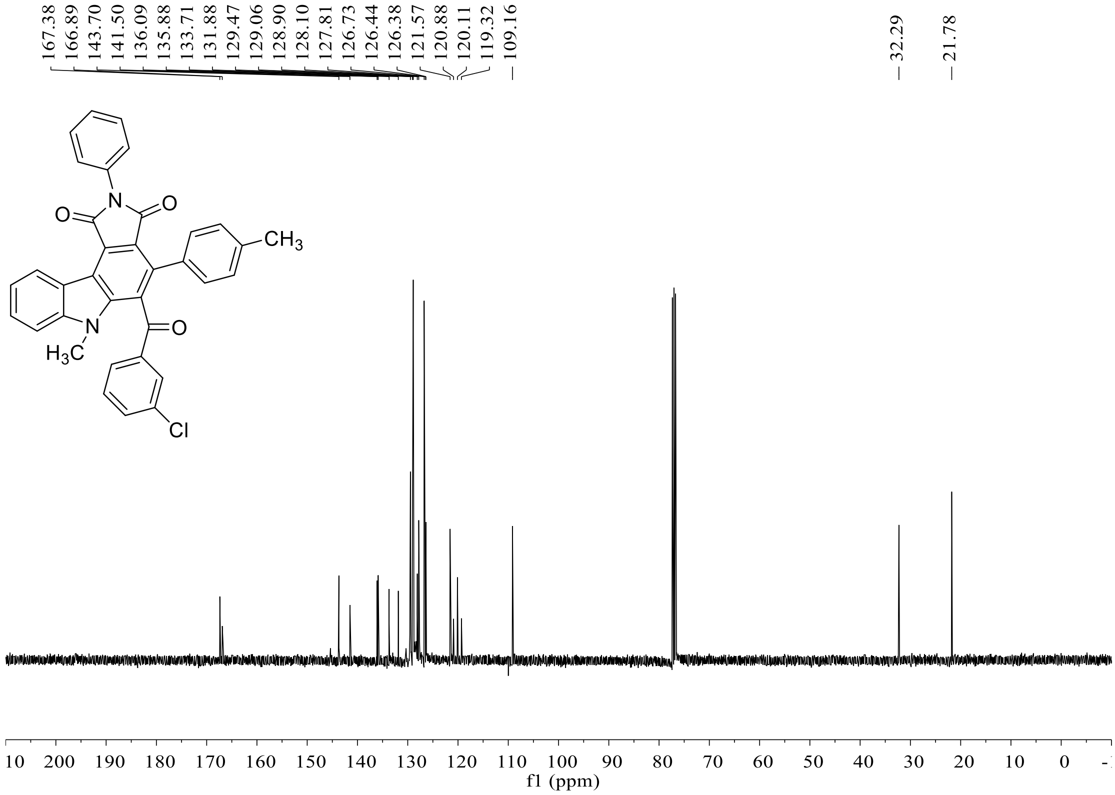
167.38
166.89
143.70
141.50
136.09
135.88
133.71
131.88
129.47
129.06
128.90
128.10
127.81
126.73
126.44
126.38
121.57
120.88
120.11
119.32
109.16
32.29
21.78
O
N
O
CH3
N
H3C
O
Cl
10
200
190
180
170
160
150
140
130
120
110
100
90
80
70
60
50
40
30
20
10
0
f1 (ppm)

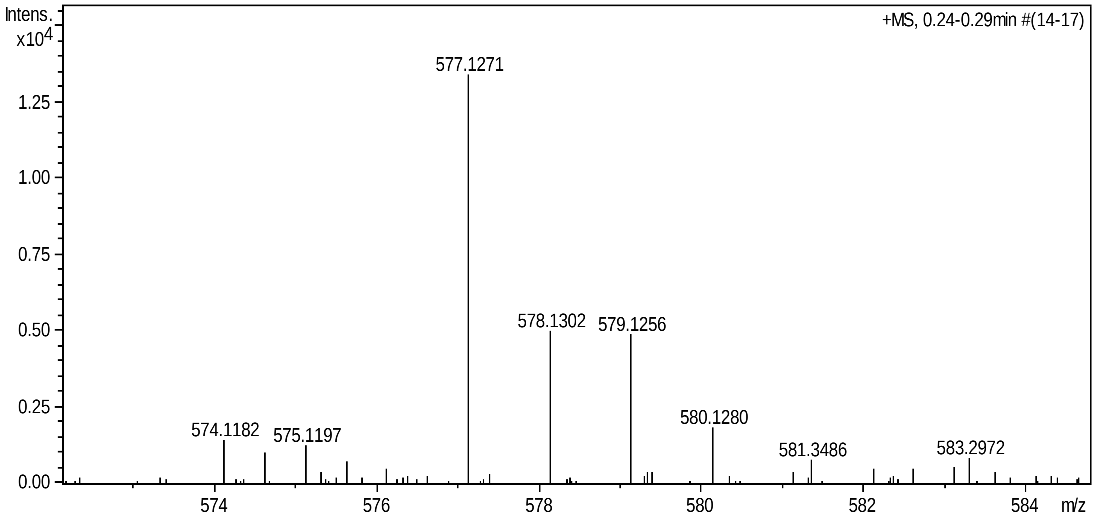
Intens.
x10^4
+MS, 0.24-0.29min #(14-17)
577.1271
1.25
1.00
0.75
578.1302
579.1256
0.50
0.25
580.1280
574.1182
575.1197
581.3486
583.2972
0.00
574
576
578
580
582
584
m/z

5-Benzoyl-2-benzyl-6-methyl-4-(*p*-tolyl)pyrrolo[3,4-*c*]carbazole-1,3(2*H*,6*H*)-dione (4k): green solid, 497 mg, 93%, m.p. >300 °C; ^{1}H NMR (400 MHz, $CDCl_3$) δ: 9.24 (d, *J* = 8.0 Hz, 1H, ArH), 7.61 (t, *J* = 8.0 Hz, 1H, ArH), 7.56 (d, *J* = 7.6 Hz, 2H, ArH), 7.49-7.44 (m, 3H, ArH), 7.43-7.37 (m, 2H, ArH), 7.32-7.28 (m, 4H, ArH), 7.25-7.23 (m, 1H, ArH), 7.10-6.94 (m, 4H, ArH), 4.86 (s, 2H, CH), 3.57 (s, 3H, CH_3), 2.25 (s, 3H, CH_3); ^{13}C {^{1}H} NMR (100 MHz, $CDCl_3$) δ: 197.1, 168.2, 167.8, 143.5, 141.4, 138.3, 137.6, 136.8, 135.5, 133.7, 131.2, 129.4, 128.8, 128.7, 128.6, 128.5, 127.6, 127.0, 126.9, 126.2, 121.3, 120.4, 120.1, 119.9, 108.9, 41.5, 32.2, 21.2; IR (KBr) υ: 2967, 1876, 1754, 1632, 1556, 1468, 1378, 1323, 1186, 1023, 965, 785, 736 cm^{-1}; MS (*m*/*z*): HRMS (ESI-TOF) Calcd. for $C_{36}H_{26}NaN_2O_3$ ([M+Na]$^+$): 557.1836, Found: 557.1829.

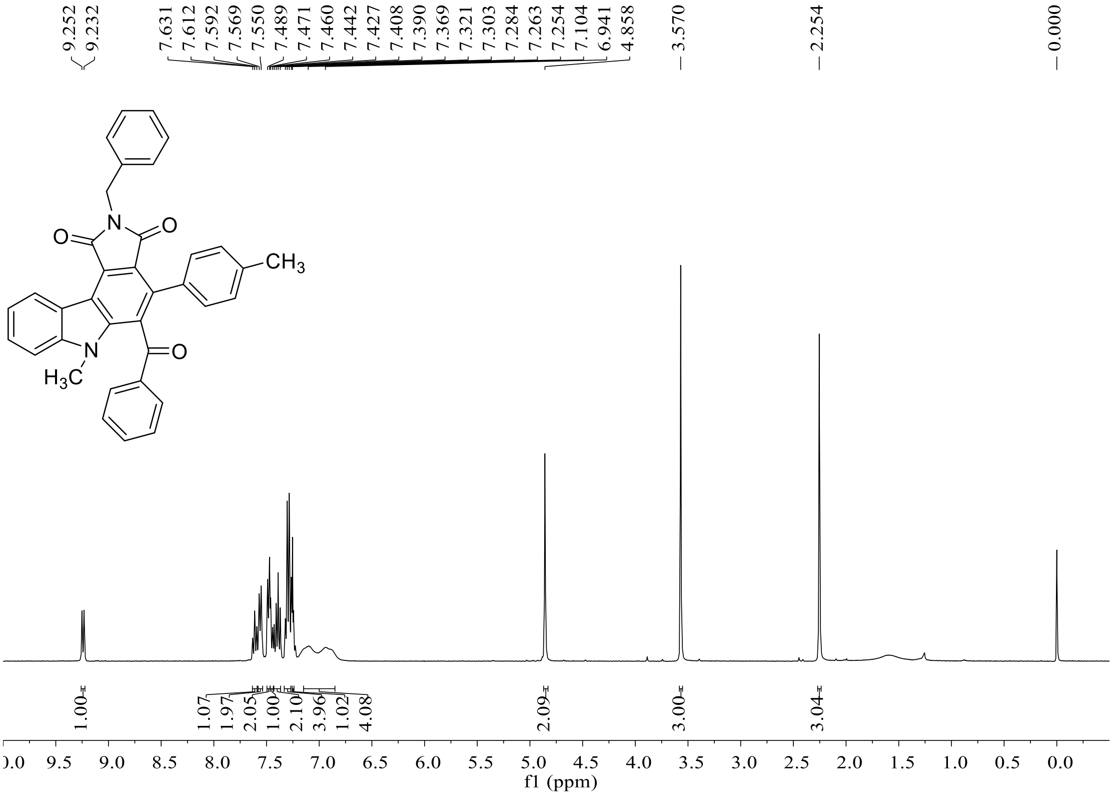

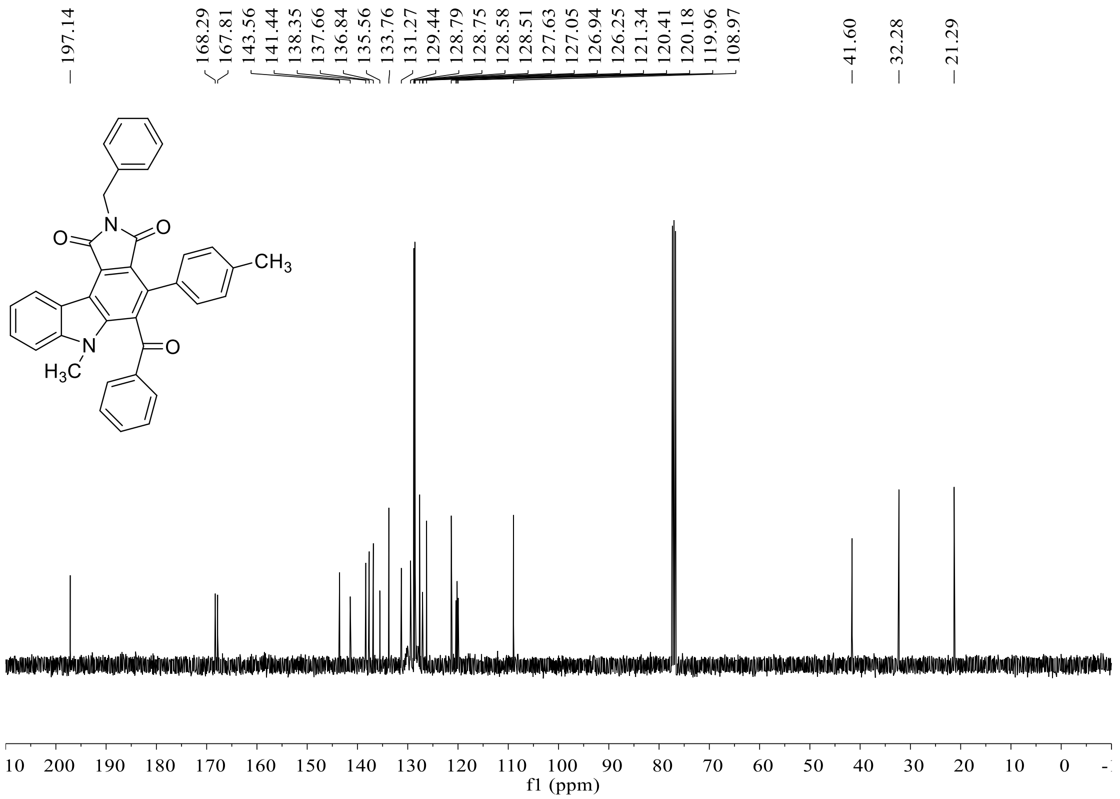
197.14
168.29
167.81
143.56
141.44
138.35
137.66
136.84
135.56
133.76
131.27
129.44
128.79
128.75
128.58
128.51
127.63
127.05
126.94
126.25
121.34
120.41
120.18
119.96
108.97
41.60
32.28
21.29
N
O
O
CH3
N
H3C
O
200
190
180
170
160
150
140
130
120
110
100
90
80
70
60
50
40
30
20
10
0
f1 (ppm)

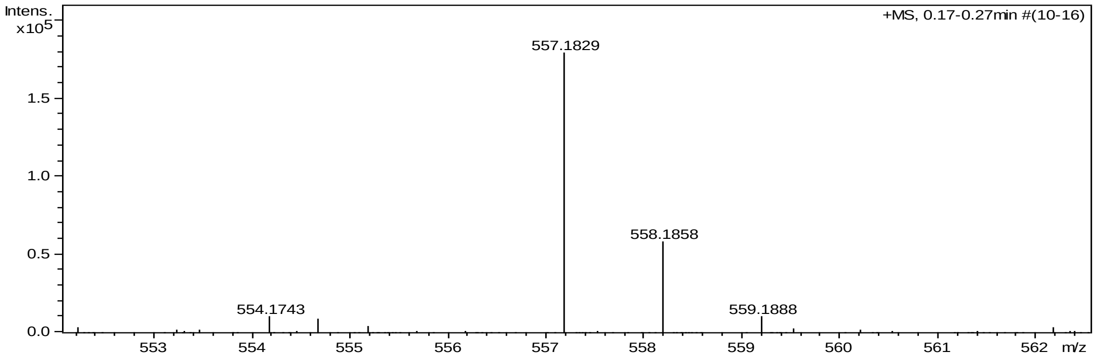
Intens.
x10^5
+MS, 0.17-0.27min #(10-16)
557.1829
558.1858
554.1743
559.1888
1.5
1.0
0.5
0.0
553
554
555
556
557
558
559
560
561
562
m/z

2-Benzyl-5-(4-chlorobenzoyl)-6-methyl-4-phenylpyrrolo[3,4-*c*]carbazole-1,3(2*H*,6*H*)-dione (4l): yellow solid, 482 mg, 87%, m.p. 215-217 °C; ^{1}H NMR (400 MHz, $CDCl_3$) δ: 9.25 (d, *J* = 8.0 Hz, 1H, ArH), 7.66-7.62 (m, 1H, ArH), 7.50-7.47 (m, 4H, ArH), 7.45-7.40 (m, 2H, ArH), 7.33-7.27 (m, 4H, ArH), 7.24-7.11 (m, 6H, ArH), 4.86 (s, 2H, CH), 3.58 (s, 3H, CH_3); ^{13}C {^{1}H} NMR (100 MHz, $CDCl_3$) δ: 195.8, 168.1, 167.6, 143.6, 141.4, 140.3, 136.7, 136.6, 135.2, 134.1, 130.6, 130.3, 130.2, 130.2, 130.2, 128.9, 128.8, 128.7, 128.5, 128.1, 127.6, 127.1, 126.3, 121.5, 120.7, 120.1, 119.8, 109.0, 41.6, 32.3; IR (KBr) υ: 1835, 1723, 1653, 1527, 1468, 1367, 1321, 1186, 1023, 986, 785, 768 cm^{-1}; MS (*m*/*z*): HRMS (ESI-TOF) Calcd. for $C_{35}H_{23}ClNaN_2O_3$ ($[M+Na]^+$): 577.1289, Found: 577.1271.

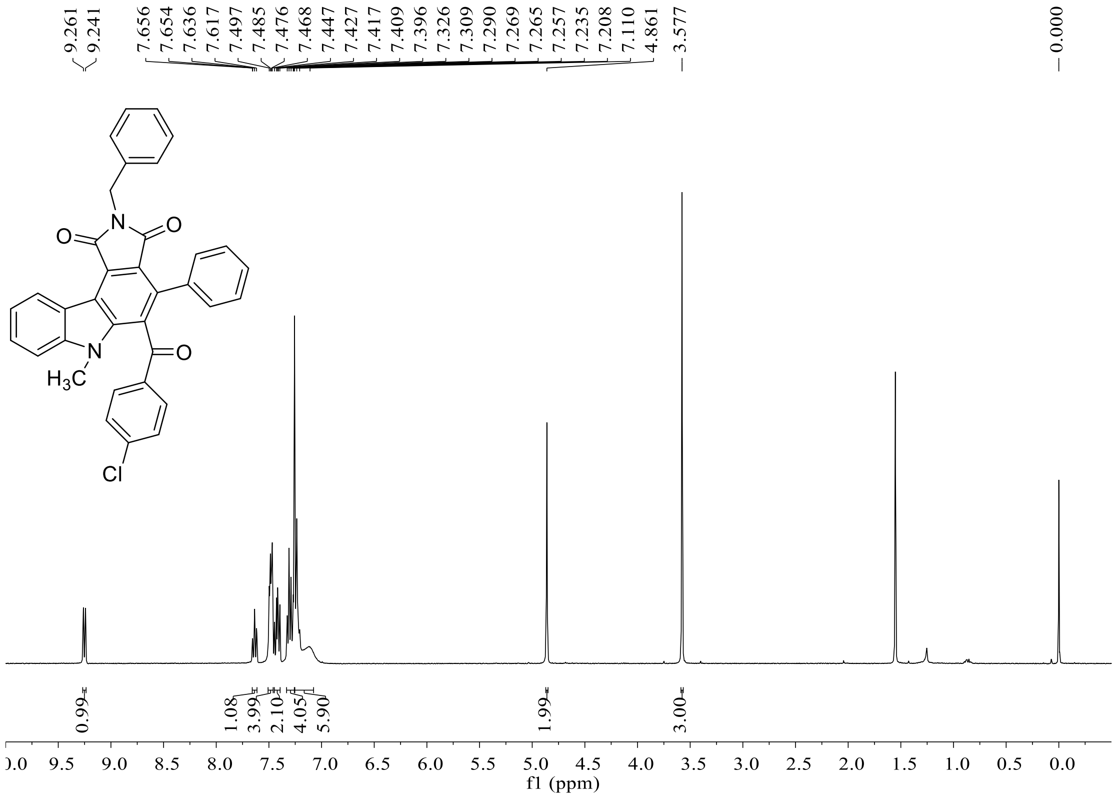

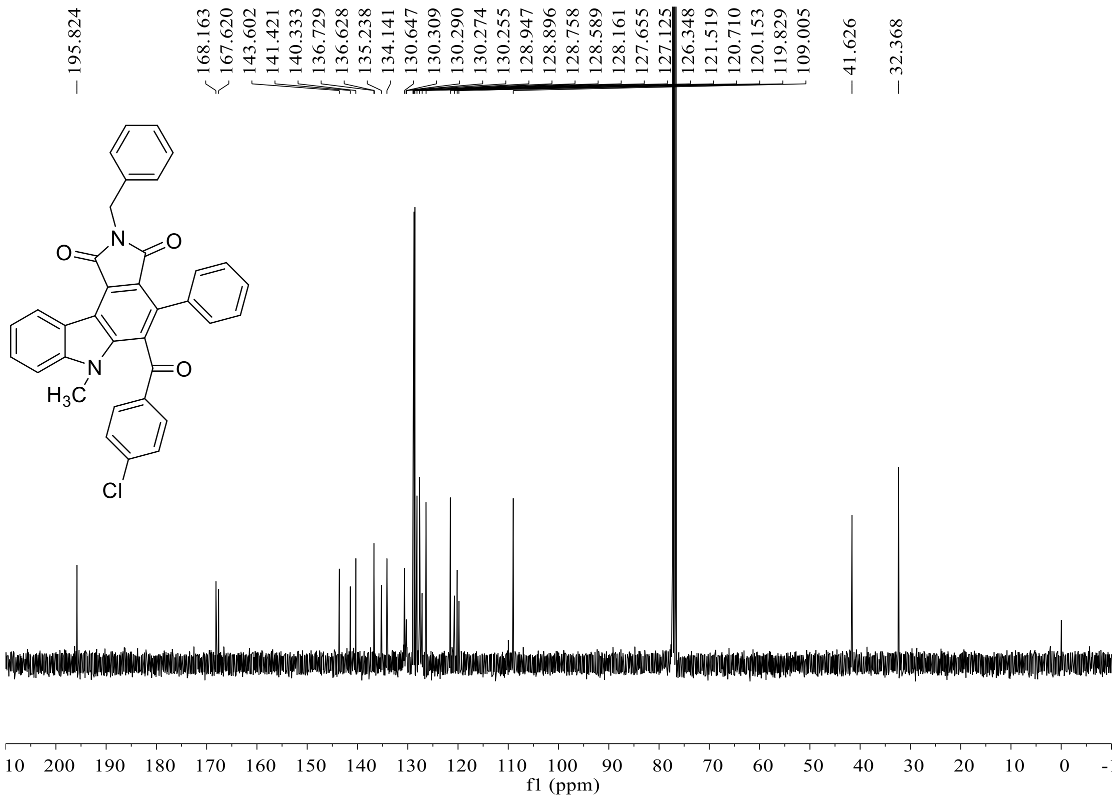
195.824
168.163
167.620
143.602
141.421
140.333
136.729
136.628
135.238
134.141
130.647
130.309
130.290
130.274
130.255
128.947
128.896
128.758
128.589
128.161
127.655
127.125
126.348
121.519
120.710
120.153
119.829
109.005
41.626
32.368
O
N
O
O
H_3C
N
Cl
f1 (ppm)

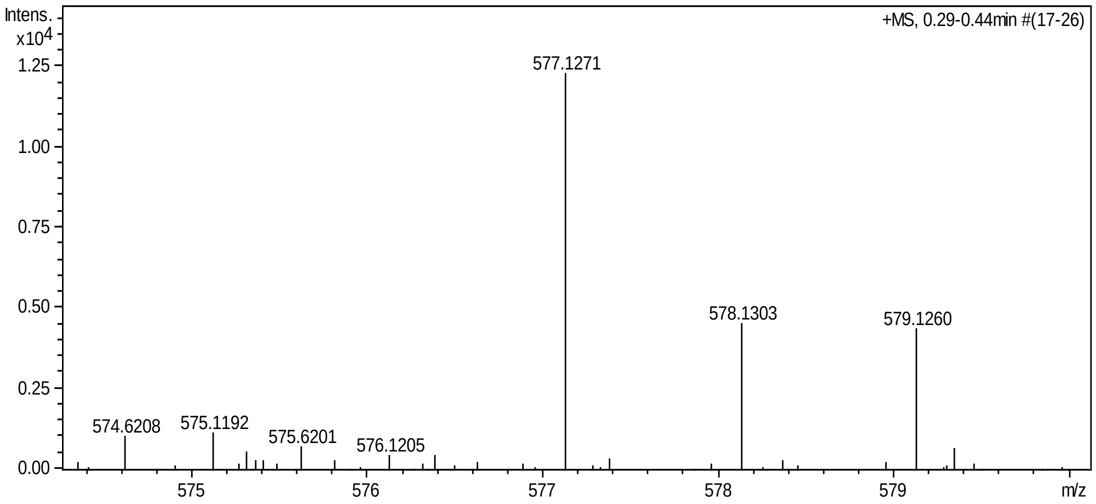
Intens.
x10^4
+MS, 0.29-0.44min #(17-26)
577.1271
578.1303
579.1260
574.6208
575.1192
575.6201
576.1205
m/z

(9-Methyl-2,4-diphenyl-9*H*-carbazole-1,3-diyl)bis(phenylmethanone) (6a):

White solid, 165 mg, 61%, m.p. 249-251 °C; ^{1}H NMR (400 MHz, $CDCl_3$) δ: 7.64 (d, *J* = 7.6 Hz, 3H, ArH), 7.43 (t, *J* = 7.8 Hz, 2H, ArH), 7.39-7.34 (m, 6H, ArH), 7.28-7.24 (m, 3H, ArH), 7.09 (t, *J* = 7.6 Hz, 2H, ArH), 7.01 (s, 2H, ArH), 6.96 (t, *J* = 7.8 Hz, 2H, ArH), 6.84 (d, *J* = 6.8 Hz, 4H, ArH), 3.64 (s, 3H, CH_3); ^{13}C NMR (400 MHz, $CDCl_3$) δ: 198.5, 198.4, 142.4, 138.9, 138.7, 137.6, 137.5, 136.8, 135.9, 135.2, 133.3, 132.2, 131.5, 131.4, 129.5, 129.2, 128.3, 128.2, 127.8, 127.6, 127.0, 126.9, 126.4, 122.4, 122.0, 121.8, 121.6, 119.6, 108.7, 32.1; IR(KBr) υ: 3057, 3023, 2907, 2360, 2339, 1720, 1605, 1482, 1320, 1267, 1172, 1009, 936, 805, 743, 612, 447 cm^{-1}; MS (*m*/*z*): HRMS (ESI) Calcd. for $C_{39}H_{27}NO_2$ ([M+Na]$^+$): 564.1934, found: 564.1926.

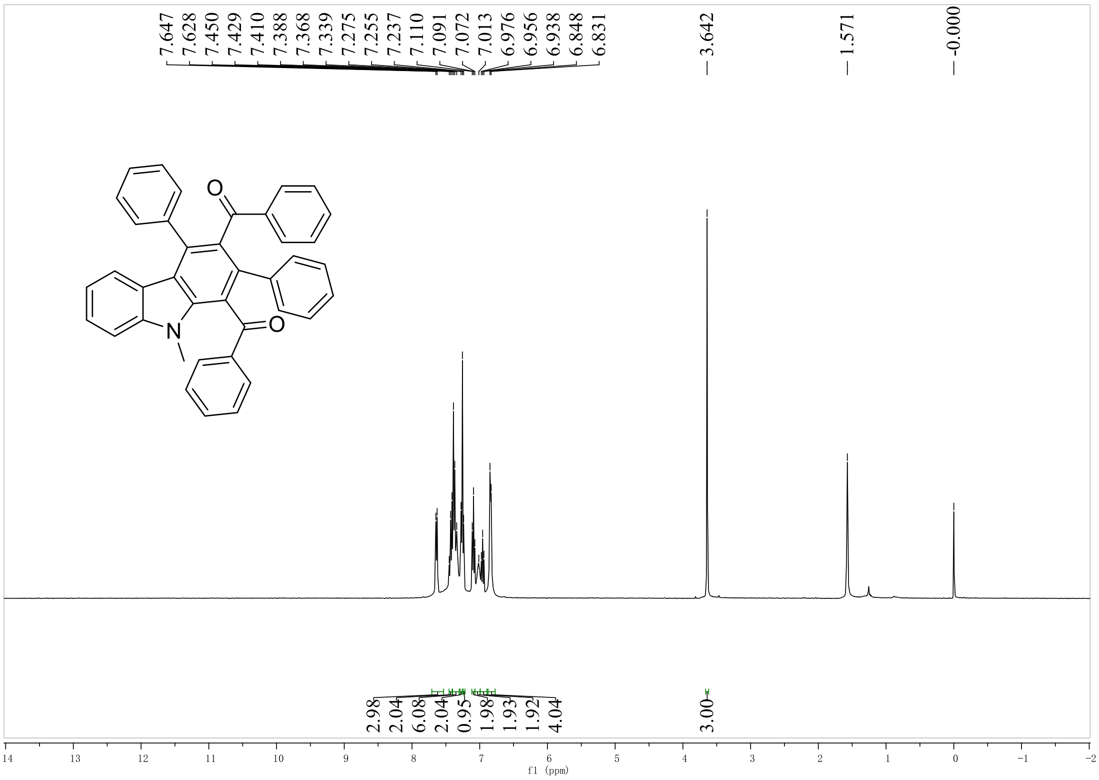

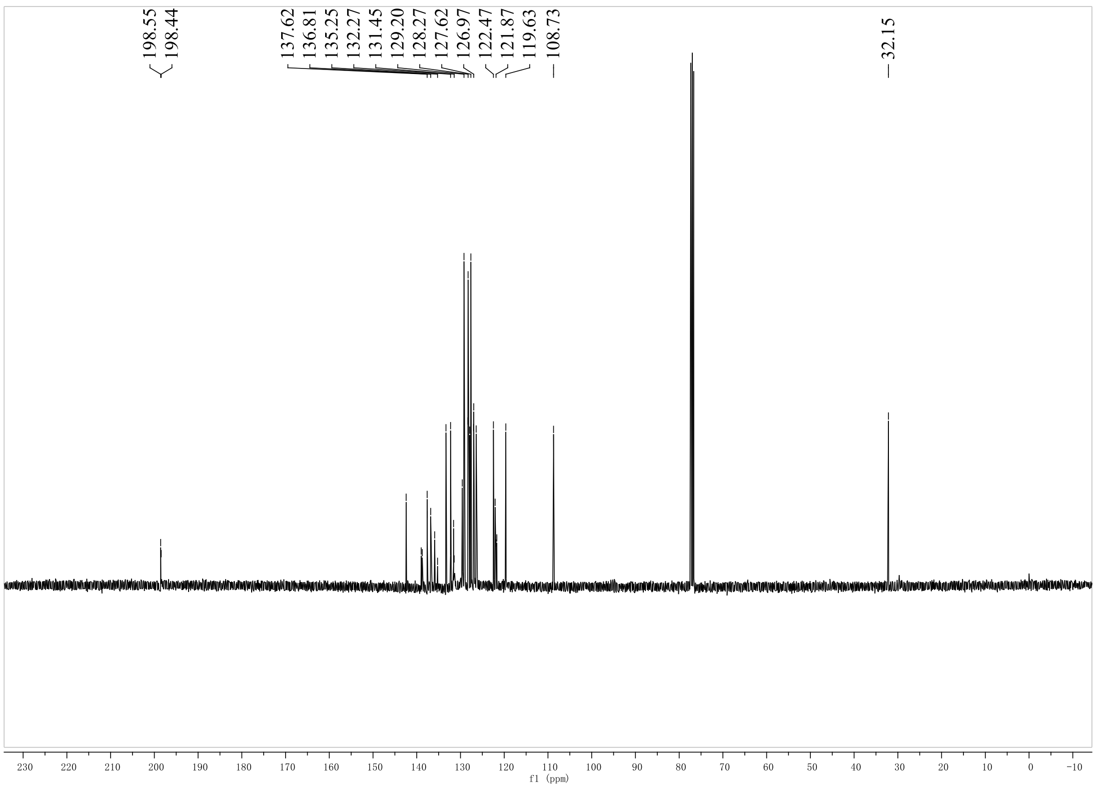
198.55
198.44
137.62
136.81
135.25
132.27
131.45
129.20
128.27
127.62
126.97
122.47
121.87
119.63
108.73
32.15
f1 (ppm)

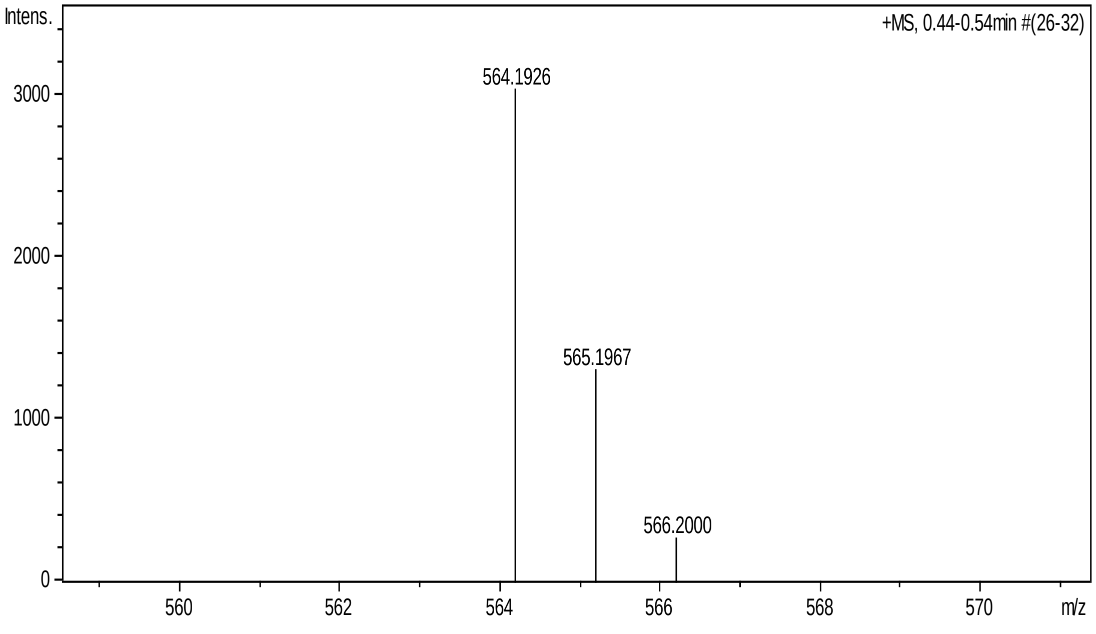
Intens.
+MS, 0.44-0.54min #(26-32)
564.1926
565.1967
566.2000
m/z

(9-Methyl-2,4-di-*p*-tolyl-9*H*-carbazole-1,3-diyl)bis(*p*-tolylmethanone) (6b):

White solid, 188 mg, 63%, m.p. 174-175 °C; ^{1}H NMR (400 MHz, $CDCl_3$) δ: 7.55 (d, *J* = 7.2 Hz, 2H, ArH), 7.41 (t, *J* = 7.6 Hz, 1H, ArH), 7.35 (s, 1H, ArH), 7.33-7.26 (m, 4H, ArH), 7.06 (d, *J* = 7.6 Hz, 4H, ArH), 6.95 (t, *J* = 7.6 Hz, 2H, ArH), 6.89 (t, *J* = 7.6 Hz, 4H, ArH), 6.66 (s, 2H, ArH), 3.60 (s, 3H, CH_3), 2.36 (s, 3H, CH_3), 2.32 (s, 3H, CH_3), 2.25 (s, 3H, CH_3) , 2.04 (s, 3H, CH_3); ^{13}C NMR (400 MHz, $CDCl_3$) δ: 198.3, 198.0, 144.2, 142.8, 142.3, 137.4, 137.2, 136.6, 136.4, 135.6, 135.1, 134.6, 133.8, 132.0, 131.4, 129.8, 129.4, 129.0, 128.2, 127.6, 126.1, 122.4, 122.2, 121.9, 121.4, 119.4, 108.5, 31.9, 21.7, 21.5, 21.3, 20.9; IR(KBr) υ: 3025, 2919, 2863, 2339, 1720, 1605, 1482, 1320, 1267, 1172, 1009, 937, 818, 773, 670, 447 cm^{-1}; MS (*m*/*z*): HRMS (ESI) Calcd. for $C_{43}H_{35}NO_2$ ($[M+Na]^+$): 620.2560, found: 620.2565.

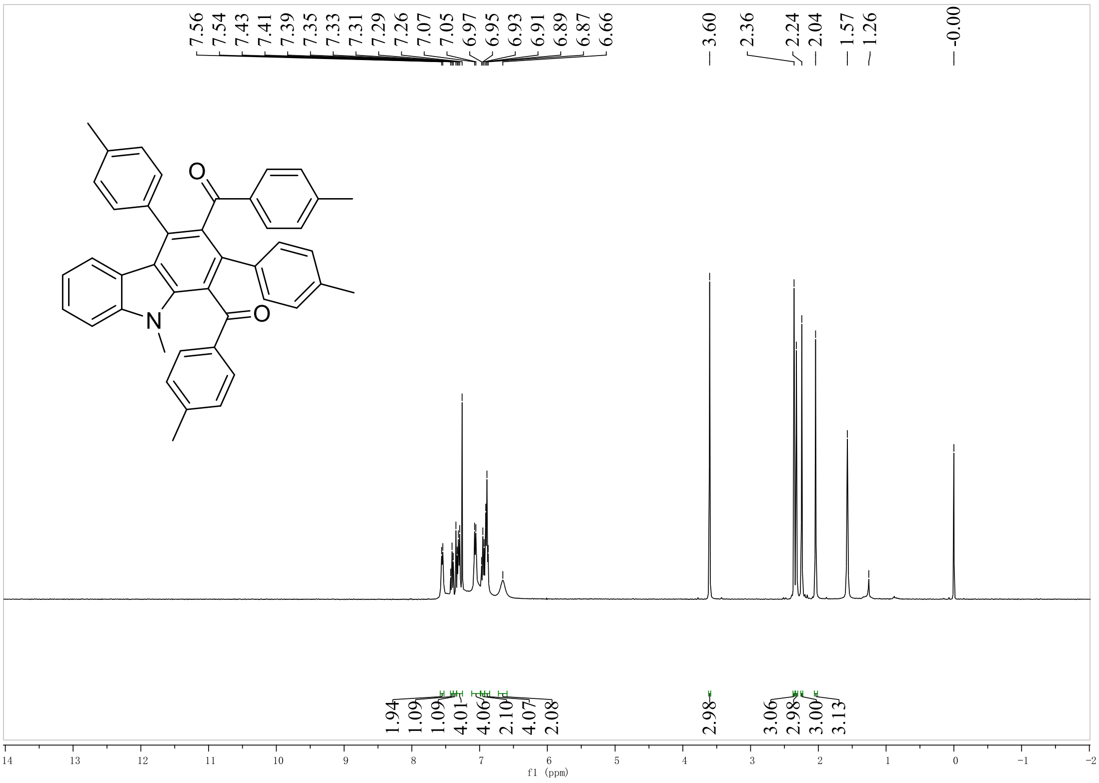

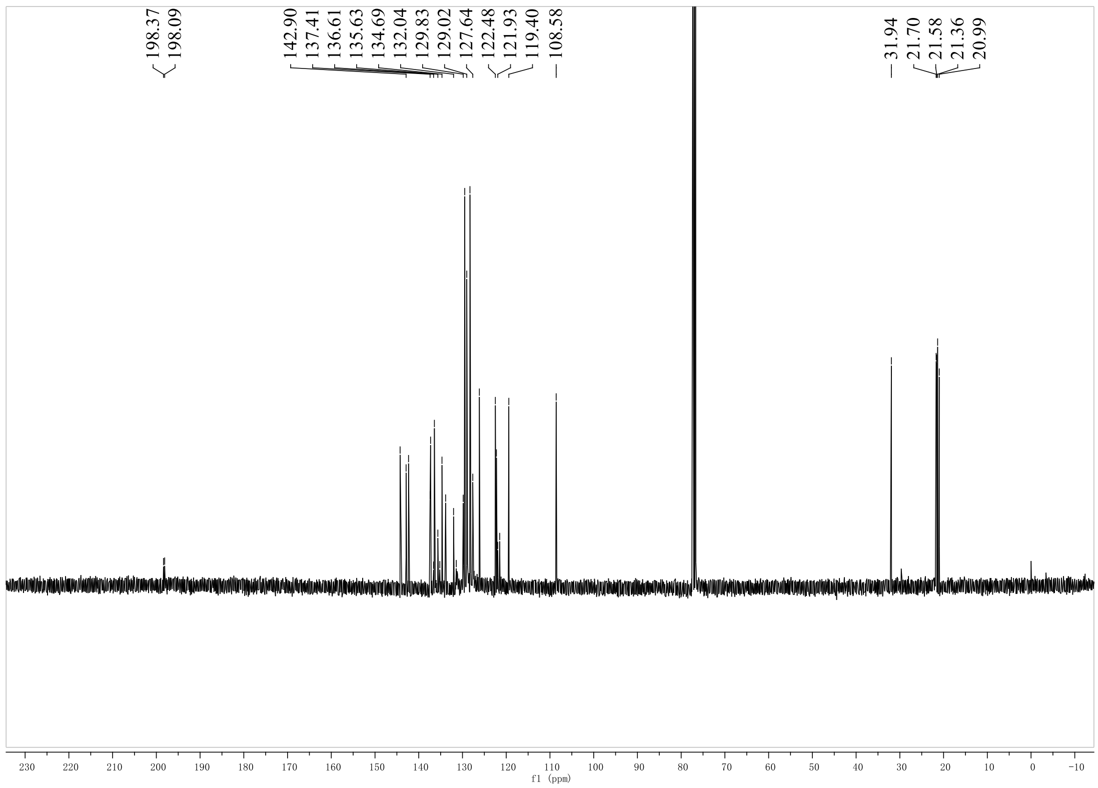
198.37
198.09
142.90
137.41
136.61
135.63
134.69
132.04
129.83
129.02
127.64
122.48
121.93
119.40
108.58
31.94
21.70
21.58
21.36
20.99
f1 (ppm)

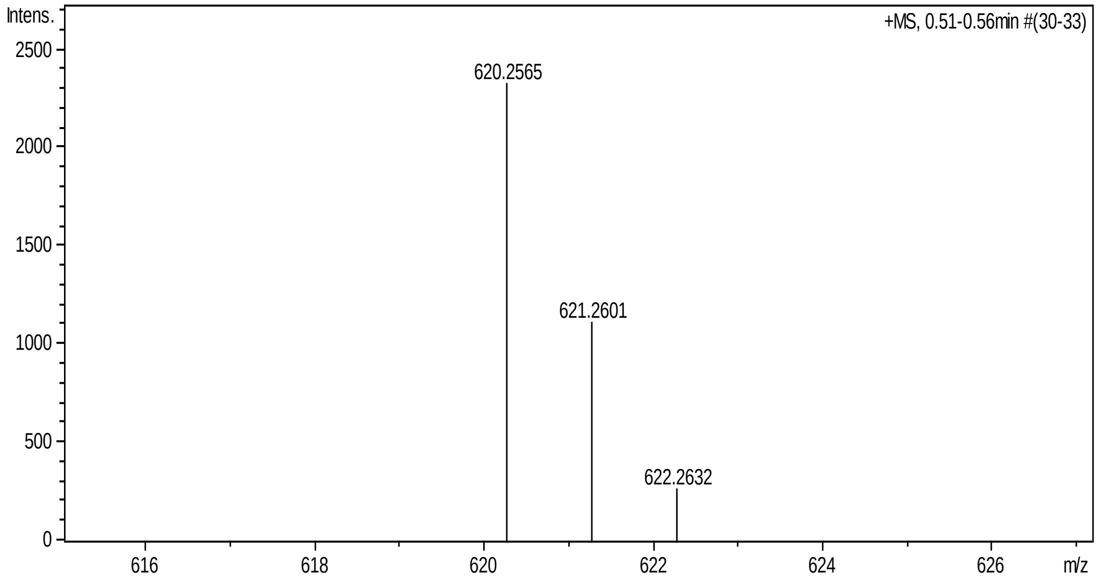
Intens.
+MS, 0.51-0.56min #(30-33)
620.2565
621.2601
622.2632
m/z

(2-(Chlorophenyl)-9-methyl-4-(*p*-tolyl)-9*H*-carbazole-1,3-diyl)bis(*p*-tolylmethanone) (6c):

White solid, 210 mg, 68%, m.p. 171-173 °C; ^{1}H NMR (400 MHz, $CDCl_3$) δ: 7.54 (d, J = 7.2 Hz, 2H, ArH), 7.44-7.34 (m, 3H, ArH), 7.30-7.26 (m, 3H, ArH), 7.09 (d, J = 8.0 Hz, 4H, ArH), 6.97 (t, J = 7.6 Hz, 2H, ArH), 6.93-6.89 (m, 4H, ArH), 6.84 (s, 2H, ArH), 3.61 (s, 3H, CH_3), 2.36 (s, 3H, CH_3), 2.34 (s, 3H, CH_3), 2.26 (s, 3H, CH_3); ^{13}C NMR (400 MHz, $CDCl_3$) δ: 198.1, 197.7, 144.7, 143.3, 142.4, 137.4, 137.3, 136.4, 136.2, 135.7, 135.4, 134.4, 133.1, 131.7, 129.7, 129.7, 129.5, 129.4, 129.2, 129.0, 128.9, 128.7, 128.6, 128.5, 128.4, 127.1, 126.4, 122.5, 122.1, 121.9, 121.8, 119.5, 108.6, 31.9, 21.7, 21.5, 21.3; IR(KBr) υ: 3021, 2917, 2863, 2339, 1721, 1605, 1481, 1320, 1267, 1172, 1009, 937, 818, 773, 670, 447 cm^{-1}; MS (m/z): HRMS (ESI) Calcd. for $C_{42}H_{32}ClNO_2$ ($[M+Na]^+$): 640.2014, found: 640.2004.

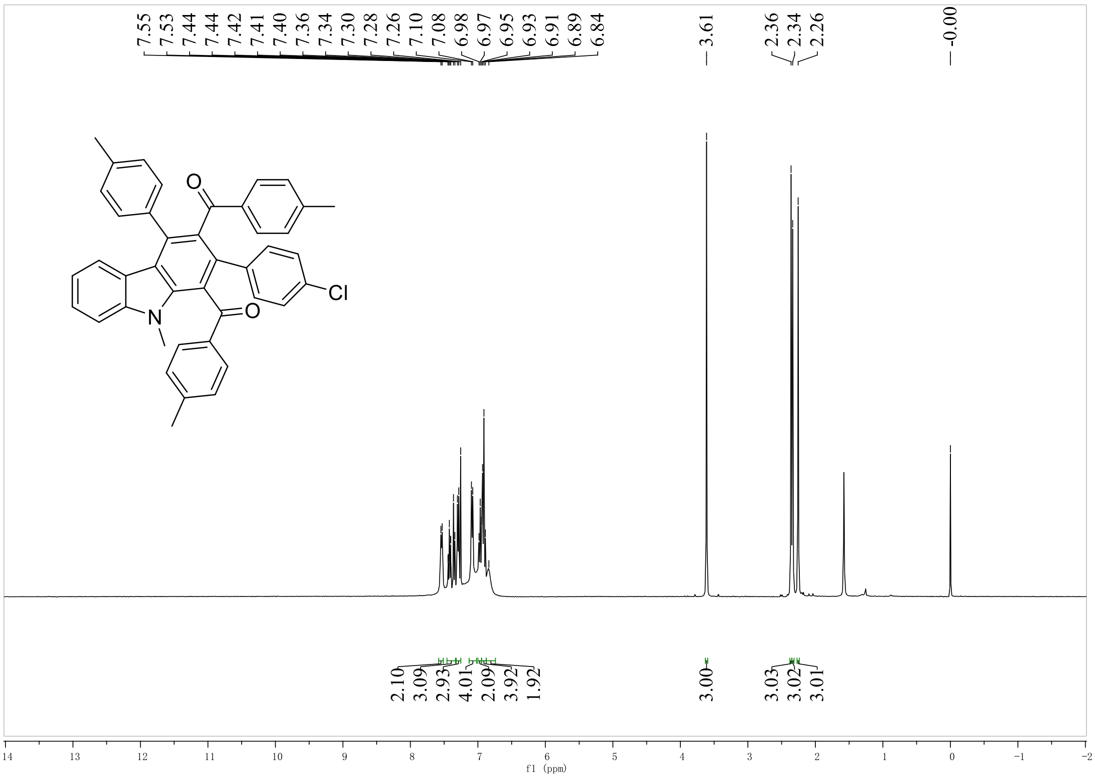

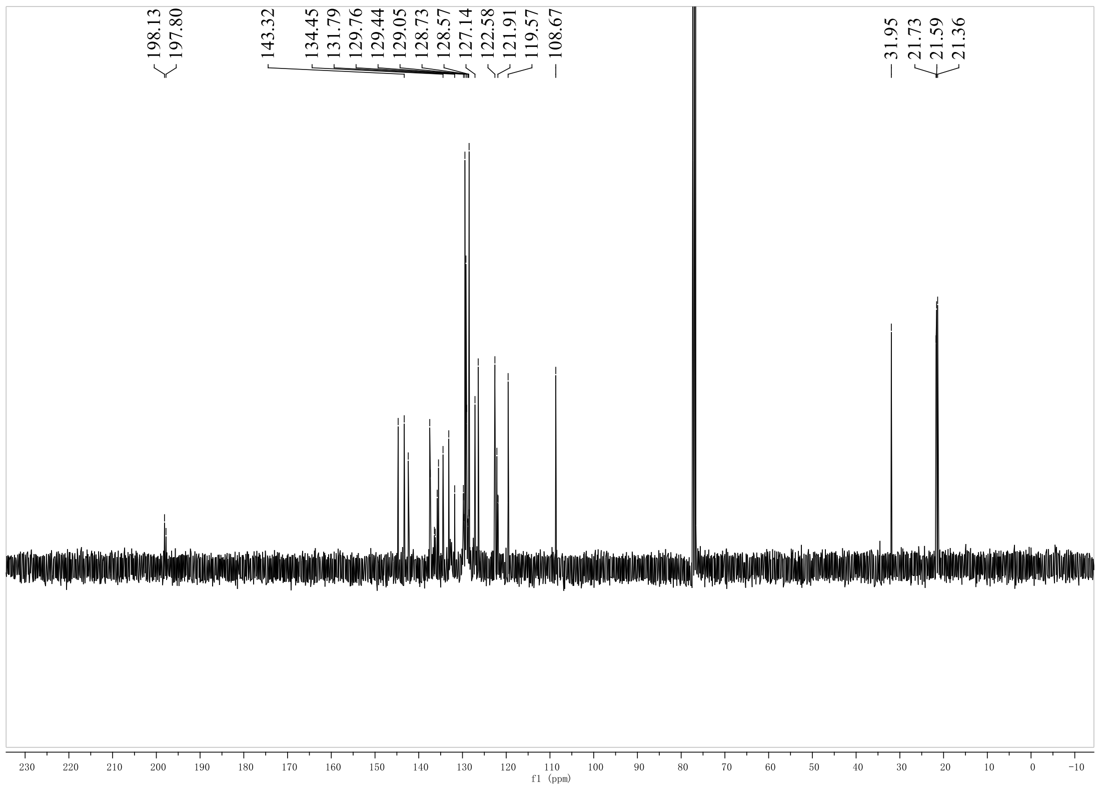

198.13
197.80
143.32
134.45
131.79
129.76
129.44
129.05
128.73
128.57
127.14
122.58
121.91
119.57
108.67
31.95
21.73
21.59
21.36
230 220 210 200 190 180 170 160 150 140 130 120 110 100 90 80 70 60 50 40 30 20 10 0 -10
f1 (ppm)

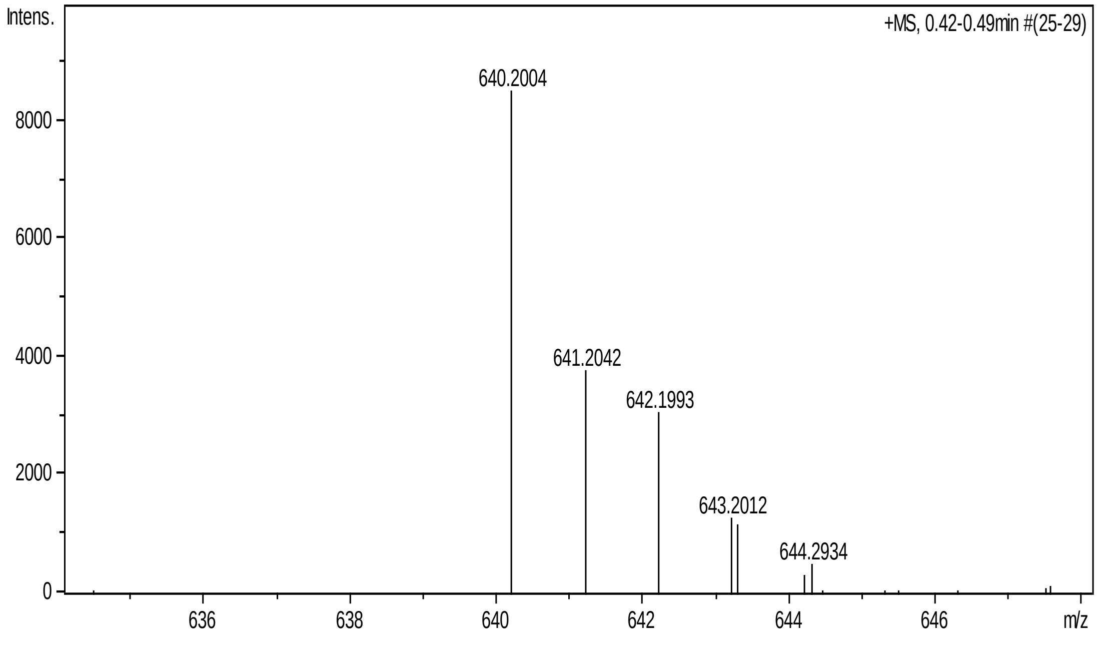

Intens.
+MS, 0.42-0.49min #(25-29)
640.2004
641.2042
642.1993
643.2012
644.2934
8000
6000
4000
2000
0
636
638
640
642
644
646
m/z

(1-(4-Methoxybenzoyl)-9-methyl-2-(*o*-tolyl)-4-(*p*-tolyl)-9*H*-carbazol-3-yl)(*p*-tolyl)methanone) (6d):

White solid, 196 mg, 64%, m.p. 168-169 °C; ^{1}H NMR (400 MHz, $CDCl_3$) δ: 7.60 (s, 2H, ArH), 7.44-7.31 (m, 5H, ArH), 7.23-7.03 (m, 3H, ArH), 6.99-6.91 (m, 4H, ArH), 6.82-6.77 (m, 4H, ArH), 6.68 (s, 1H, ArH), 6.51 (s, 1H, ArH), 3.81 (s, 3H, OCH_3), 3.62 (s, 3H, CH_3), 2.35 (s, 3H, CH_3), 2.25 (s, 3H, CH_3), 2.01 (d, *J* = 7.6 Hz, 3H, CH_3); ^{13}C NMR (400 MHz, $CDCl_3$) δ: 163.6, 159.3, 144.1, 142.9, 142.8, 142.2, 137.3, 134.6, 134.4, 132.0, 131.6, 129.2, 129.0, 128.9, 128.2, 127.4, 126.1, 122.4, 122.2, 119.4, 113.5, 113.5, 113.4, 113.3, 109.9, 108.6, 55.4, 31.7, 21.5, 21.3, 20.5; IR(KBr) υ: 3054, 2920, 1887, 1665, 1447, 1320, 1267, 1172, 1009, 952, 805, 743, 651, 462 cm^{-1}; MS (*m/z*): HRMS (ESI) Calcd. for $C_{43}H_{35}NO_4$ ($[M+Na]^+$): 636.2509, found: 636.2508.

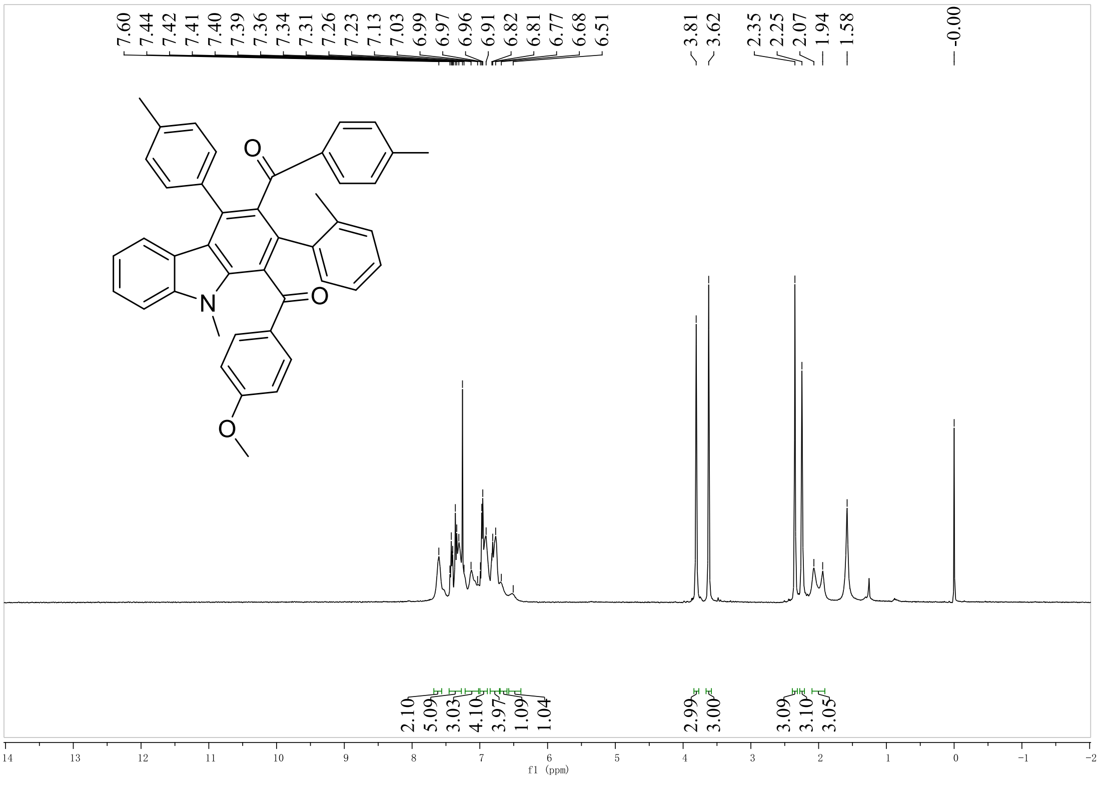

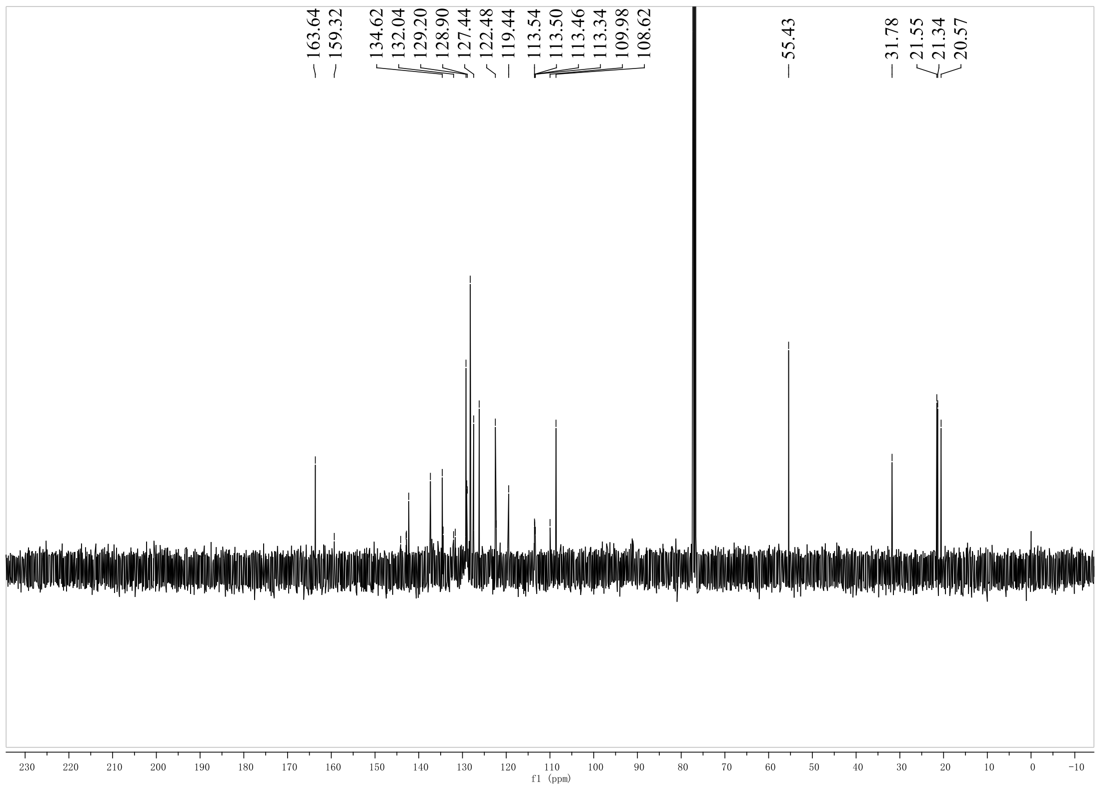
163.64
159.32
134.62
132.04
129.20
128.90
127.44
122.48
119.44
113.54
113.50
113.46
113.34
109.98
108.62
55.43
31.78
21.55
21.34
20.57
f1 (ppm)

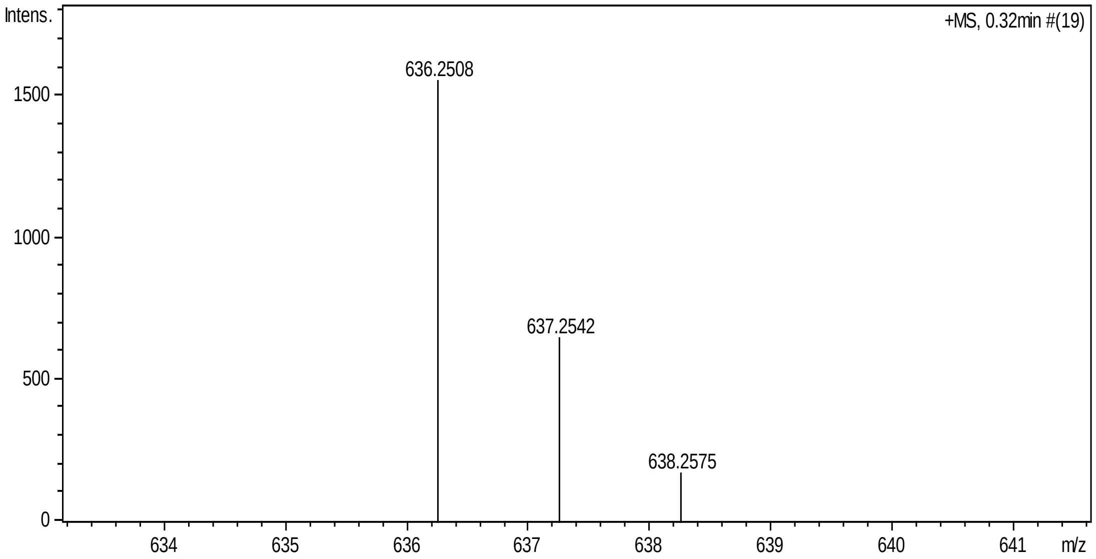
Intens.
+MS, 0.32min #(19)
636.2508
637.2542
638.2575
m/z

(1-(4-Methoxybenzoyl)-4-(4-methoxyphenyl)-9-methyl-2-(*o*-tolyl)-9*H*-carbazol-3-yl)(*p*-tolyl)methanone) (6e):

White solid, 195 mg, 62%, m.p. 161-162 °C; ^{1}H NMR (400 MHz, $CDCl_3$) δ: 7.60 (s, 2H, ArH), 7.45-7.31 (m, 5H, ArH), 7.23-7.10 (m, 1H, ArH), 6.98 (d, *J* = 6.4 Hz, 2H, ArH), 6.91 (s, 3H, ArH), 6.84-6.77 (m, 6H, ArH), 6.52 (s, 1H, ArH), 3.81 (s, 6H, OCH_3), 3.62 (s, 3H, CH_3), 2.25 (s, 3H, CH_3), 2.01 (d, *J* = 10.4 Hz, 3H, CH_3); ^{13}C NMR (400 MHz, $CDCl_3$) δ: 163.6, 159.0, 142.8, 142.8, 142.2, 140.7, 134.4, 131.2, 129.9, 129.2, 129.1, 128.2, 127.4, 126.1, 122.4, 122.2, 119.4, 113.7, 113.6, 113.5, 113.5, 113.3, 108.9, 108.6, 55.4, 55.1, 31.7, 21.5, 20.5,; IR(KBr) υ: 3055, 2920, 2360, 1664, 1433, 1320, 1267, 1131, 1009, 937, 807, 743, 636, 462 cm^{-1}; MS (*m*/*z*): HRMS (ESI) Calcd. for $C_{43}H_{35}NO_4$ ($[M+Na]^+$): 652.2458, found: 652.2464.

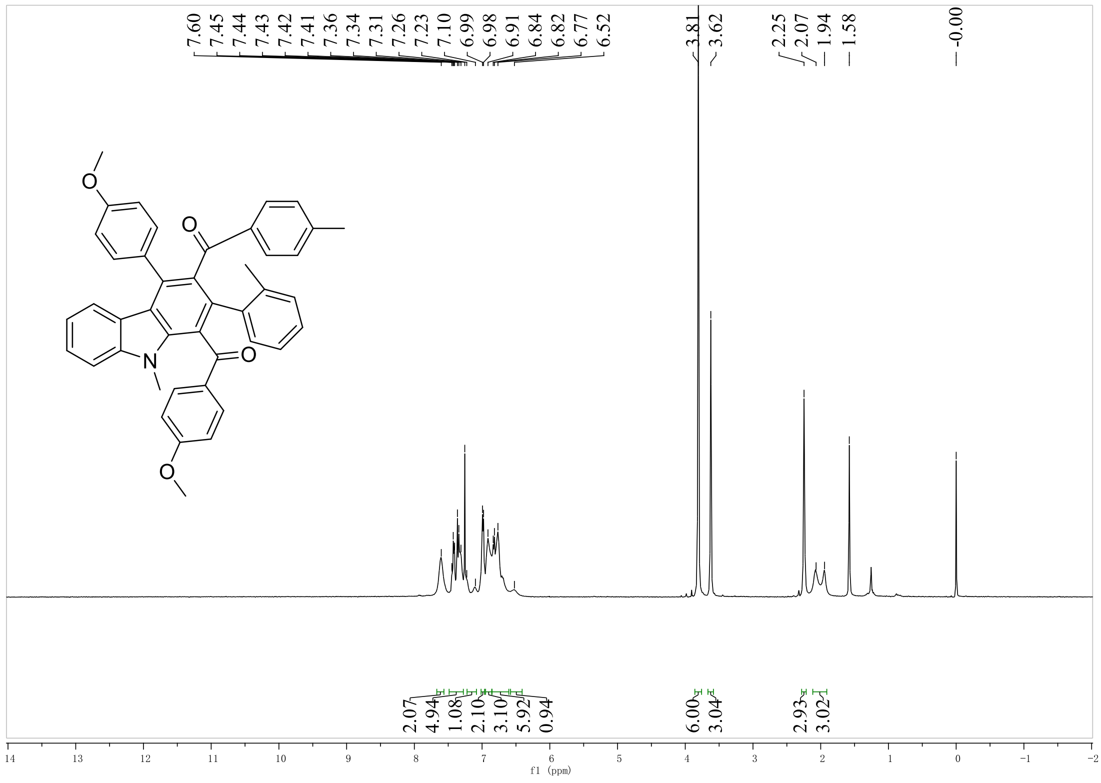

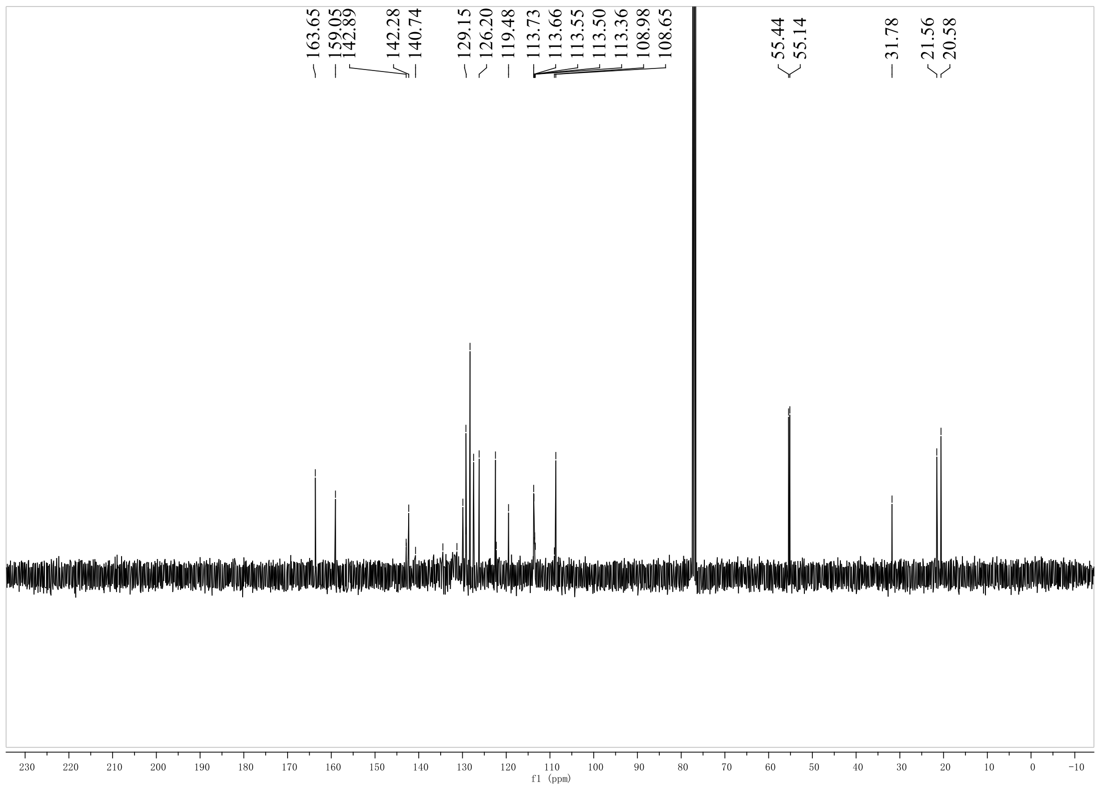
163.65
159.05
142.89
142.28
140.74
129.15
126.20
119.48
113.73
113.66
113.55
113.50
113.36
108.98
108.65
55.44
55.14
31.78
21.56
20.58
f1 (ppm)

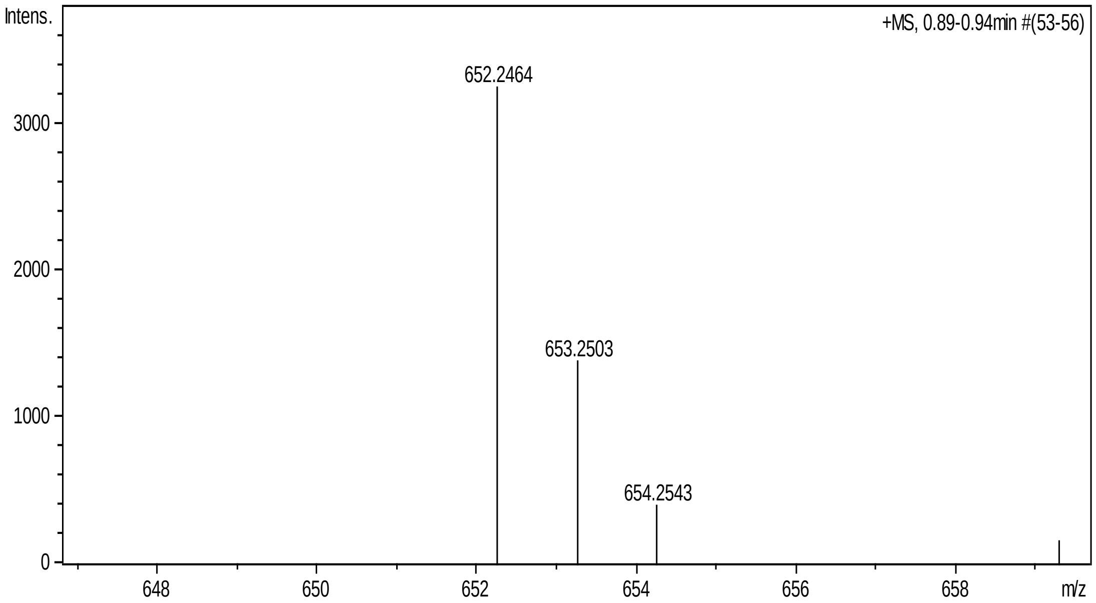
Intens.
+MS, 0.89-0.94min #(53-56)
652.2464
653.2503
654.2543
m/z

(2,4-Bis(4-methoxyphenyl)-9-methyl-3-(4-methylbenzoyl)-9*H*-carbazol-1-yl)(4-chlorophenyl)methanone) (6f):

White solid, 214 mg, 66%, m.p. 191-192 °C; ^{1}H NMR (400 MHz, $CDCl_3$) δ: 7.58 (d, J = 8.0 Hz, 3H, ArH), 7.43 (t, J = 7.2 Hz, 2H, ArH), 7.36 (d, J = 8.4 Hz, 1H, ArH), 7.29 (d, J = 7.6 Hz, 3H, ArH), 7.24 (d, J = 8.0 Hz, 2H, ArH), 6.98 (t, J = 7.6 Hz, 3H, ArH), 6.92 (t, J = 8.4 Hz, 4H, ArH), 6.42 (s, 2H, ArH), 3.82 (s, 3H, OCH_3), 3.61 (s, 3H, CH_3), 3.58 (s, 3H, CH_3), 2.24 (s, 3H, CH_3); ^{13}C NMR (400 MHz, $CDCl_3$) δ: 159.1, 158.4, 143.0, 142.3, 139.6, 139.3, 138.8, 137.4, 137.1, 135.6, 134.8, 132.4, 130.8, 129.7, 129.3, 129.0, 128.6, 128.4, 126.3, 123.6, 122.5, 122.1, 121.9, 121.3, 119.6, 113.7, 113.0, 112.6, 108.6, 55.1, 54.9, 32.1, 21.5; IR(KBr) υ: 3025, 2919, 2359, 1664, 1432, 1321, 1265, 1132, 1009, 937, 807, 743, 636, 462 cm^{-1}; MS (m/z): HRMS (ESI) Calcd. for $C_{42}H_{32}ClNO_4$ ($[M+Na]^+$): 672.1912, found: 672.1918.

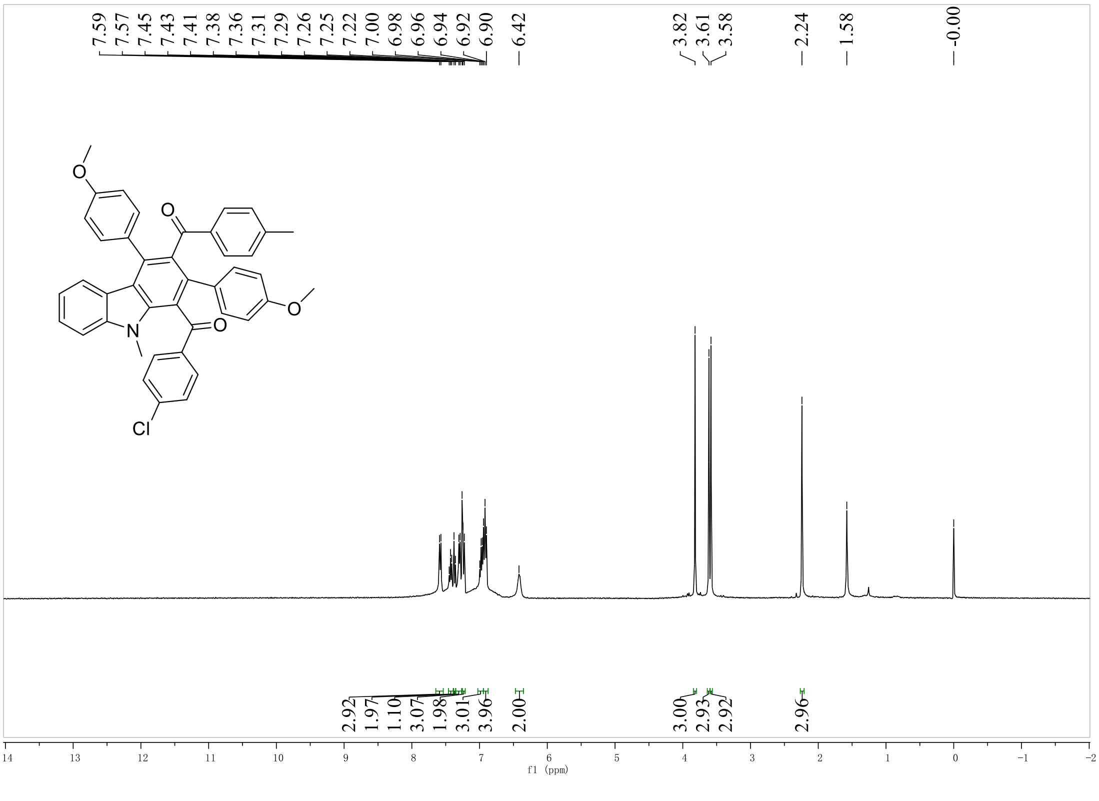

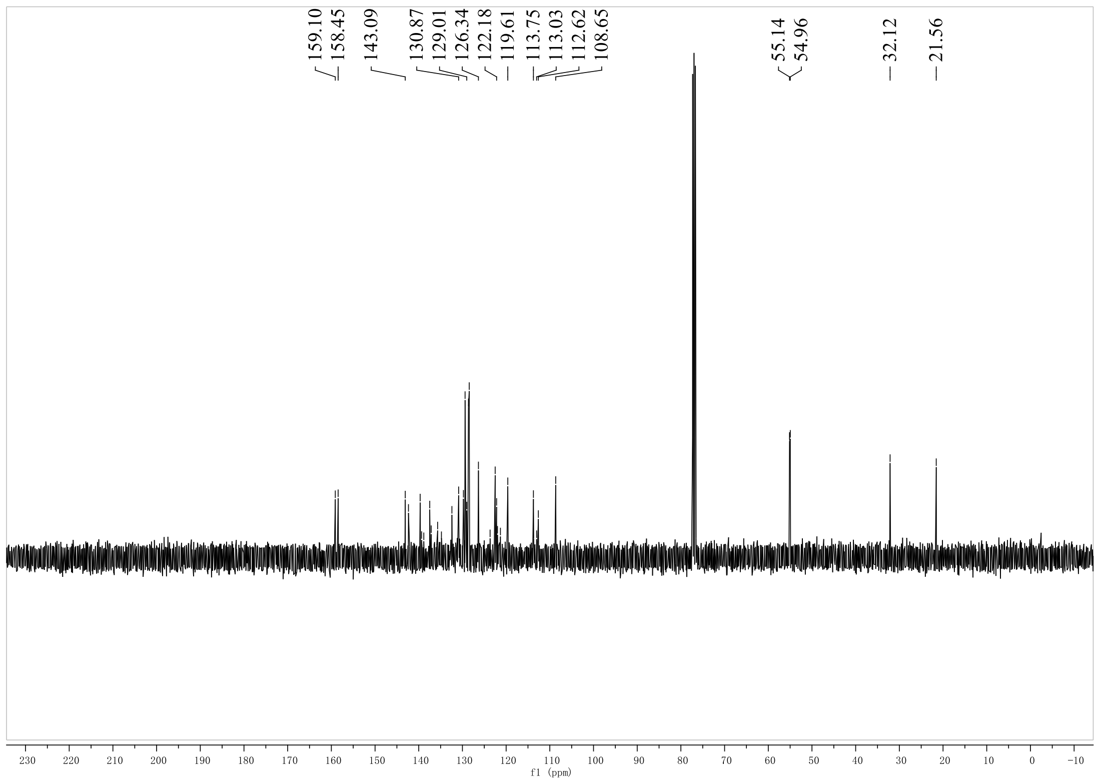
159.10
158.45
143.09
130.87
129.01
126.34
122.18
119.61
113.75
113.03
112.62
108.65
55.14
54.96
32.12
21.56
230
220
210
200
190
180
170
160
150
140
130
120
110
100
90
80
70
60
50
40
30
20
10
0
−10
f1 (ppm)

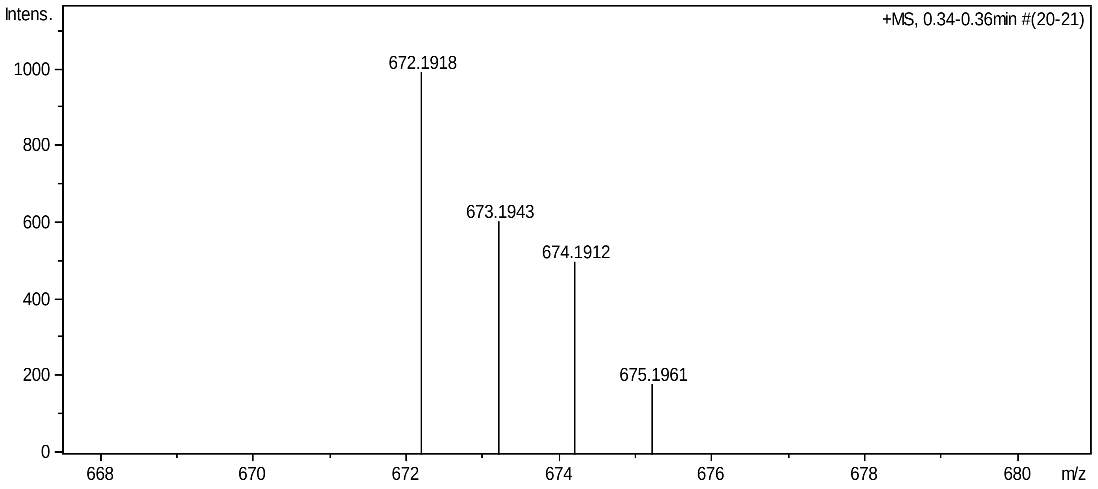
Intens.
+MS, 0.34-0.36min #(20-21)
1000
800
600
400
200
0
672.1918
673.1943
674.1912
675.1961
668
670
672
674
676
678
680
m/z

(1-Benzoyl-4-(4-chlorophenyl)-9-methyl-2-phenyl-9*H*-carbazol-3-yl)(*p*-tolyl)methanone) (6g):

White solid, 174 mg, 59%, m.p. 171-172 °C; ^{1}H NMR (400 MHz, $CDCl_3$) δ: 7.62 (d, *J* = 7.2 Hz, 2H, ArH), 7.45 (t, *J* = 7.6 Hz, 2H, ArH), 7.40-7.37 (m, 2H, ArH), 7.29-7.23 (m, 5H, ArH), 7.00 (t, *J* = 7.6 Hz, 3H, ArH), 6.90 (d, *J* = 7.6 Hz, 3H, ArH), 6.88-6.85 (m, 3H, ArH), 3.64 (s, 3H, CH_3), 2.24 (s, 3H, CH_3); ^{13}C NMR (400 MHz, $CDCl_3$) δ: 198.3, 197.9, 143.3, 142.4, 138.6, 137.5, 136.6, 136.3, 136.1, 135.1, 134.3, 133.9, 133.3, 131.8, 131.4, 131.4, 131.4, 129.5, 129.3, 128.6, 128.5, 128.2, 127.0, 126.9, 126.5, 122.2, 122.1, 121.8, 121.5, 119.7, 108.8, 32.1, 21.5; IR(KBr) υ: 3053, 2931, 2361, 1662, 1430, 1320, 1262, 1132, 1009, 937, 803, 727, 642, 479 cm^{-1}; MS (*m*/*z*): HRMS (ESI) Calcd. for $C_{40}H_{28}ClNO_2$ ([M+Na]$^{+}$): 612.1701, found: 612.1707.

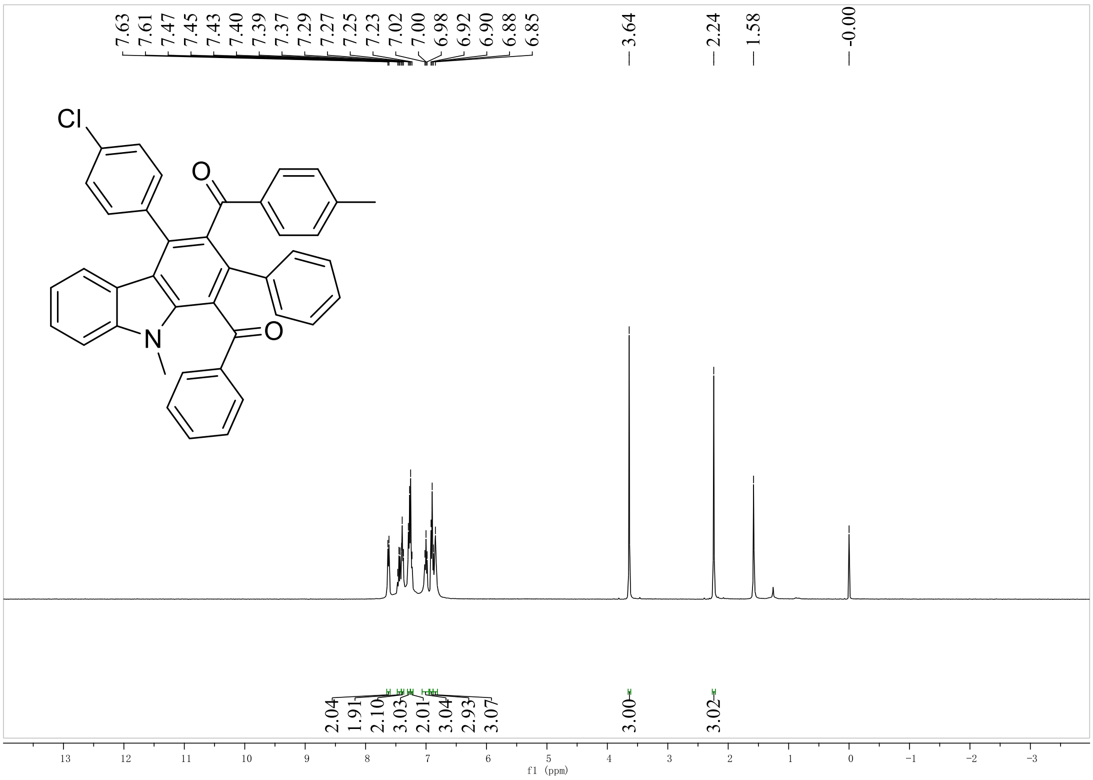

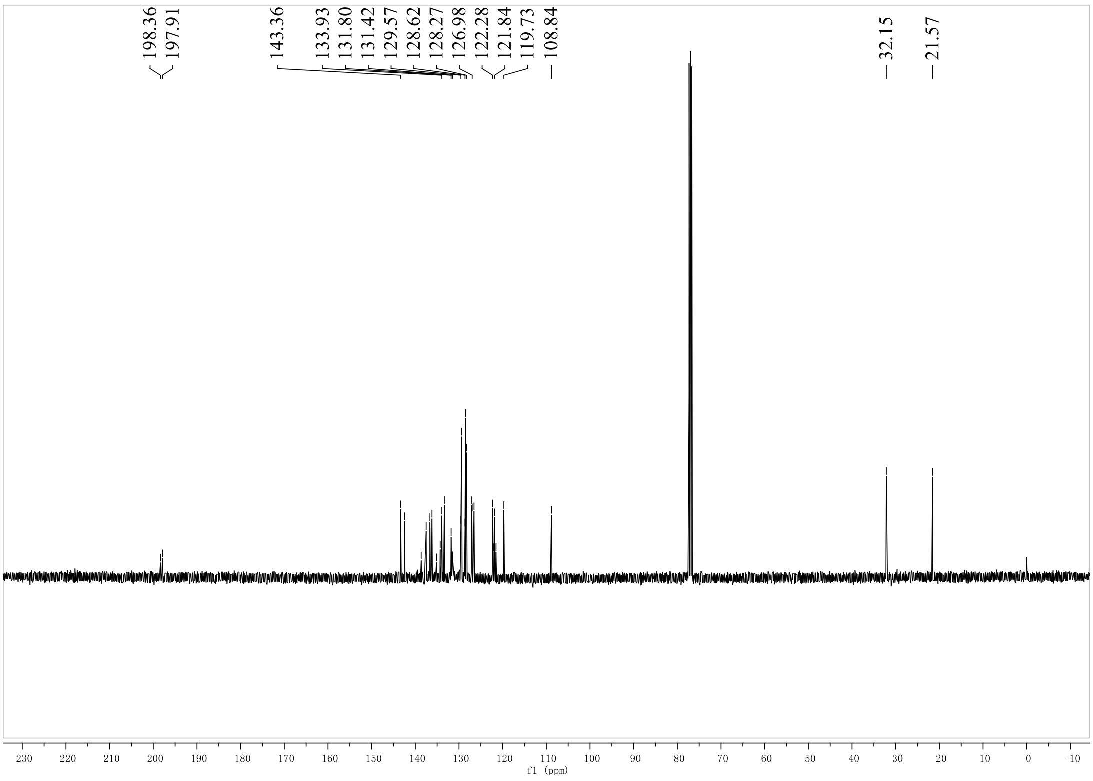
198.36
197.91
143.36
133.93
131.80
131.42
129.57
128.62
128.27
126.98
122.28
121.84
119.73
108.84
32.15
21.57
f1 (ppm)

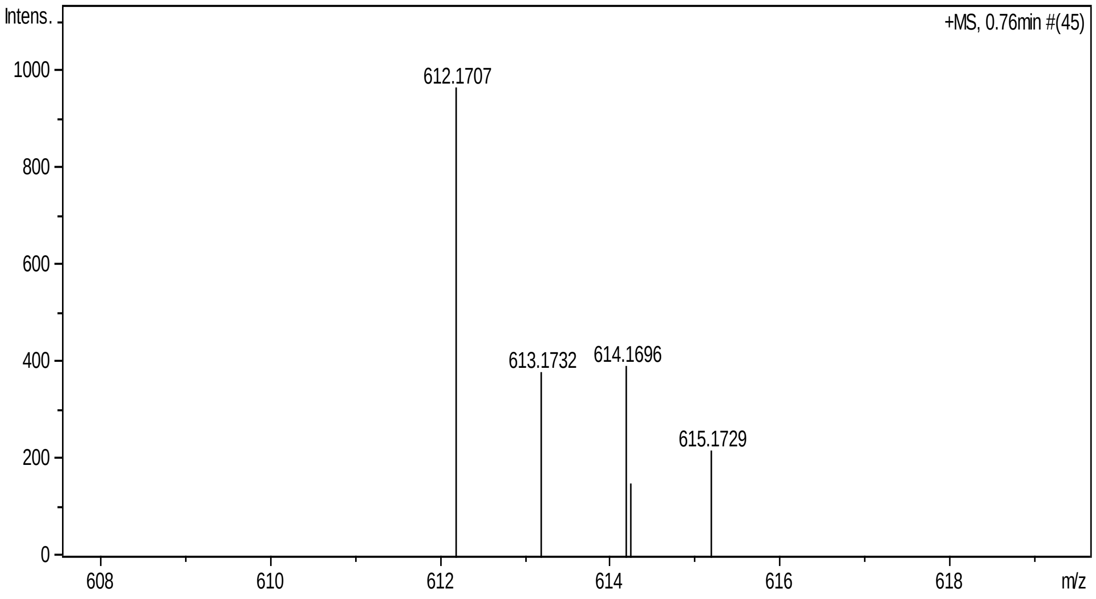
Intens.
+MS, 0.76min #(45)
612.1707
613.1732
614.1696
615.1729
m/z

(2,4-Bis(4-chlorophenyl)-9-methyl-9*H*-carbazole-1,3-diyl)bis(*p*-tolylmethanone) (6h): White solid, 191 mg, 60%, m.p. 182-183 °C; ^{1}H NMR (400 MHz, $CDCl_3$) δ: 7.54 (d, *J* = 7.6 Hz, 2H, ArH), 7.45 (t, *J* = 8.0 Hz, 2H, ArH), 7.37 (d, *J* = 8.0 Hz, 2H, ArH), 7.29-7.26 (m, 3H, ArH), 7.09 (d, *J* = 7.6 Hz, 3H, ArH), 7.00 (t, *J* = 7.2 Hz, 2H, ArH), 6.98 (d, *J* = 8.0 Hz, 3H, ArH), 6.90-6.85 (m, 3H, ArH), 3.62 (s, 3H, CH_3), 2.34 (s, 3H, CH_3), 2.27 (s, 3H, CH_3); ^{13}C NMR (400 MHz, $CDCl_3$) δ: 197.8, 197.6, 144.8, 143.6, 142.4, 137.3, 136.2, 136.0, 135.2, 134.1, 134.0, 133.6, 133.2, 131.7, 129.7, 129.4, 129.2, 128.6, 127.2, 126.6, 122.3, 121.7, 121.6, 119.7, 108.8, 31.9, 21.7, 21.6; IR(KBr) υ: 30531, 2932, 2360, 1661, 1430, 1320, 1262, 1132, 1009, 937, 804, 726, 643, 478 cm^{-1}; MS (*m*/*z*): HRMS (ESI) Calcd. for $C_{41}H_{29}Cl_2NO_2$ ([M+Na]$^+$): 660.1468, found: 660.1469.

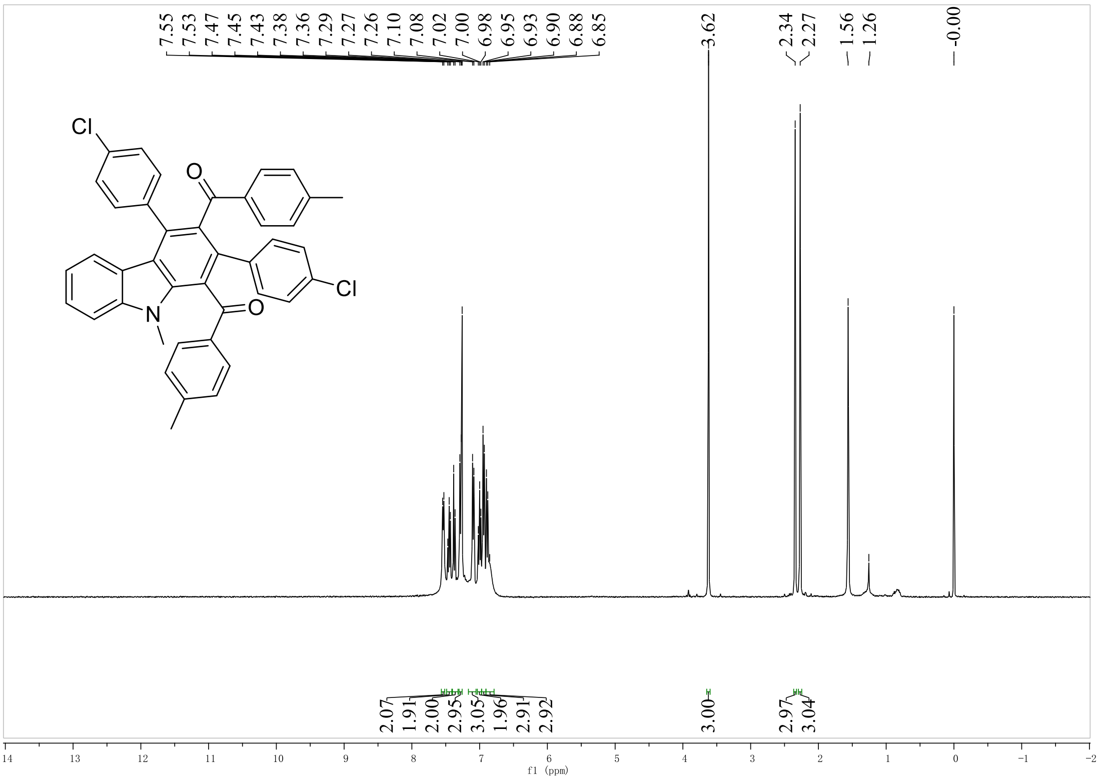

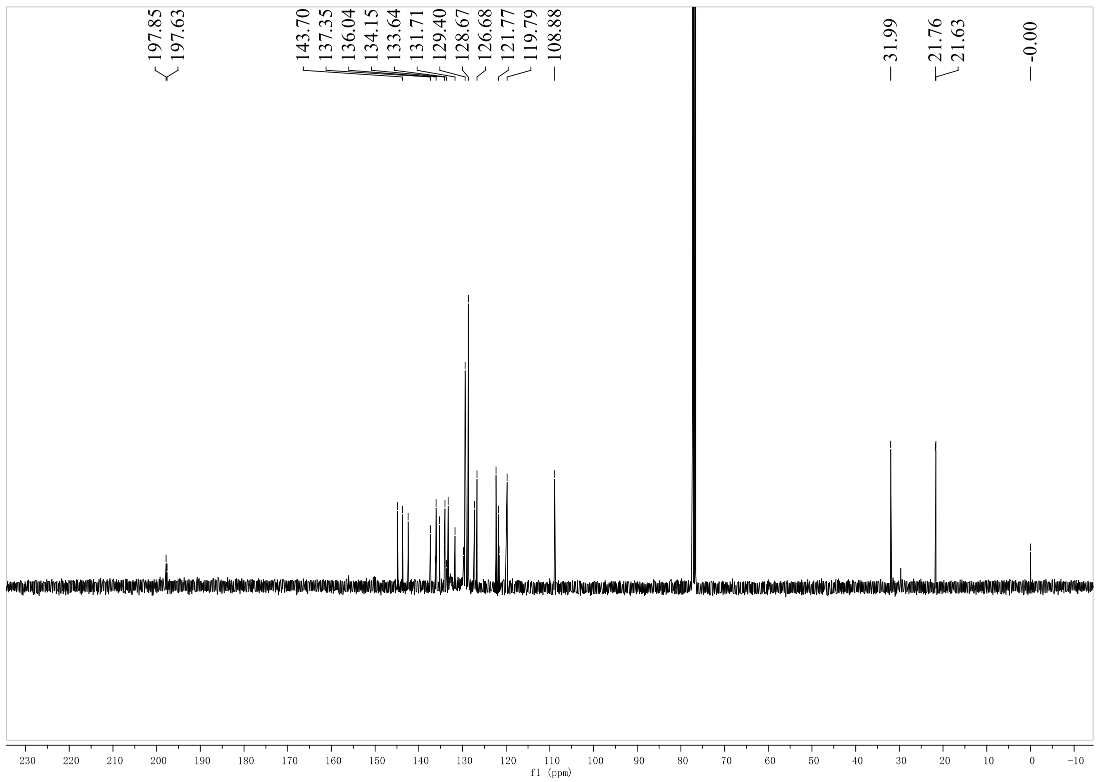
197.85
197.63
143.70
137.35
136.04
134.15
133.64
131.71
129.40
128.67
126.68
121.77
119.79
108.88
31.99
21.76
21.63
-0.00
f1 (ppm)

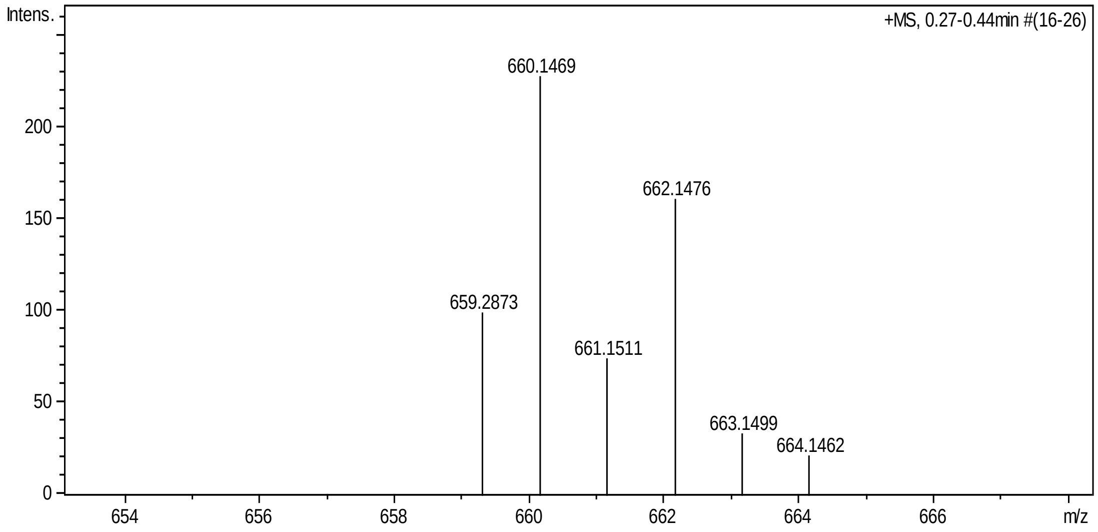
Intens.
+MS, 0.27-0.44min #(16-26)
660.1469
662.1476
659.2873
661.1511
663.1499
664.1462
m/z

(4-(4-Chlorophenyl)-1-(4-methoxybenzoyl)-9-methyl-2-(4-nitrophenyl)-9*H*-carbazol-3-yl)(*p*-tolyl)methanone) (6i):

White solid, 229 mg, 69%, m.p. 196-197 °C; ^{1}H NMR (400 MHz, $CDCl_3$) δ: 7.76 (d, *J* = 27.2 Hz, 2H, ArH), 7.62 (s, 2H, ArH), 7.52-7.38 (m, 4H, ArH), 7.32-7.27 (m, 3H, ArH), 7.26-7.17 (m, 3H, ArH), 7.01 (t, *J* = 7.2 Hz, 1H, ArH), 6.93 (t, *J* = 8.4 Hz, 1H, ArH), 6.70 (d, *J* = 8.4 Hz, 2H, ArH), 3.81 (s, 3H, OCH_3), 3.65 (s, 3H, CH_3), 2.26 (s, 3H, CH_3); ^{13}C NMR (400 MHz, $CDCl_3$) δ: 197.4, 195.8, 164.2, 146.5, 144.1, 144.1, 142.5, 137.2, 135.9, 135.7, 134.2, 134.1, 132.4, 131.4, 131.2, 129.3, 129.0, 128.8, 128.8, 128.7, 128.7, 128.6, 126.9, 122.4, 122.1, 122.1, 121.6, 119.9, 114.0, 108.9, 55.5, 31.9, 21.6; IR(KBr) υ: 3055, 2934, 2363, 1661, 1431, 1321, 1262, 1133, 1007, 935, 803, 727, 644, 476 cm^{-1}; MS (*m*/*z*): HRMS (ESI) Calcd. for $C_{41}H_{29}ClN_2O_5$ ($[M+Na]^+$): 687.1657, found: 687.1663.

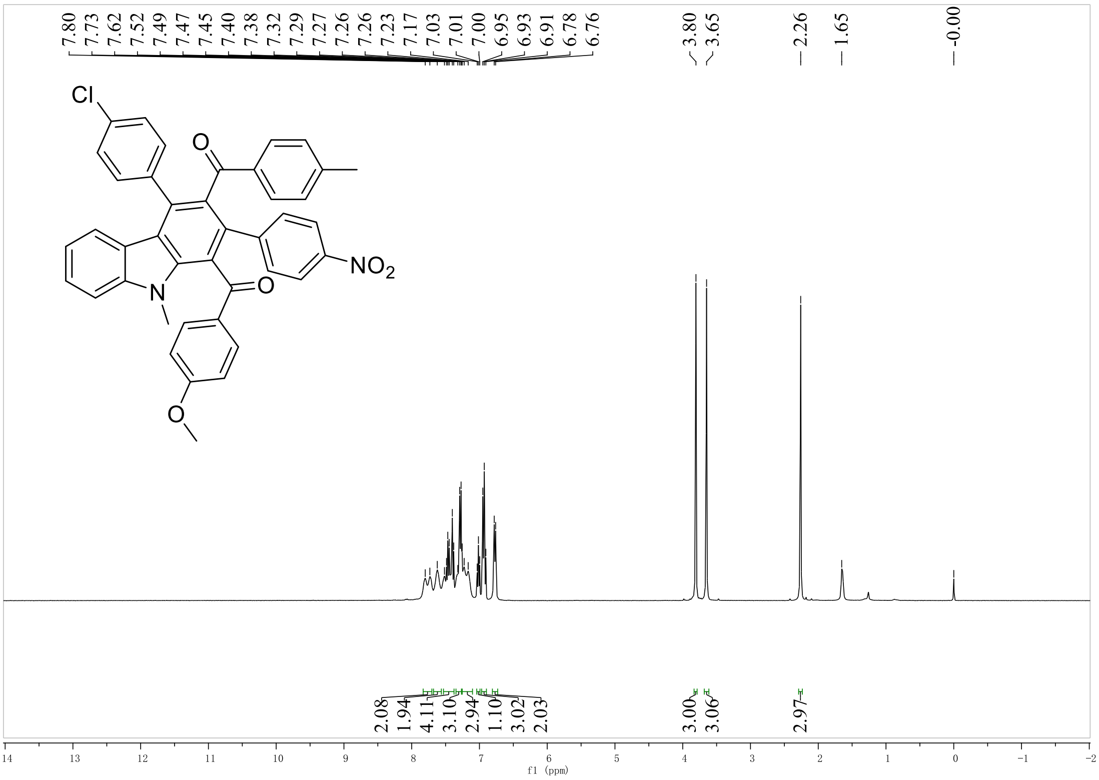

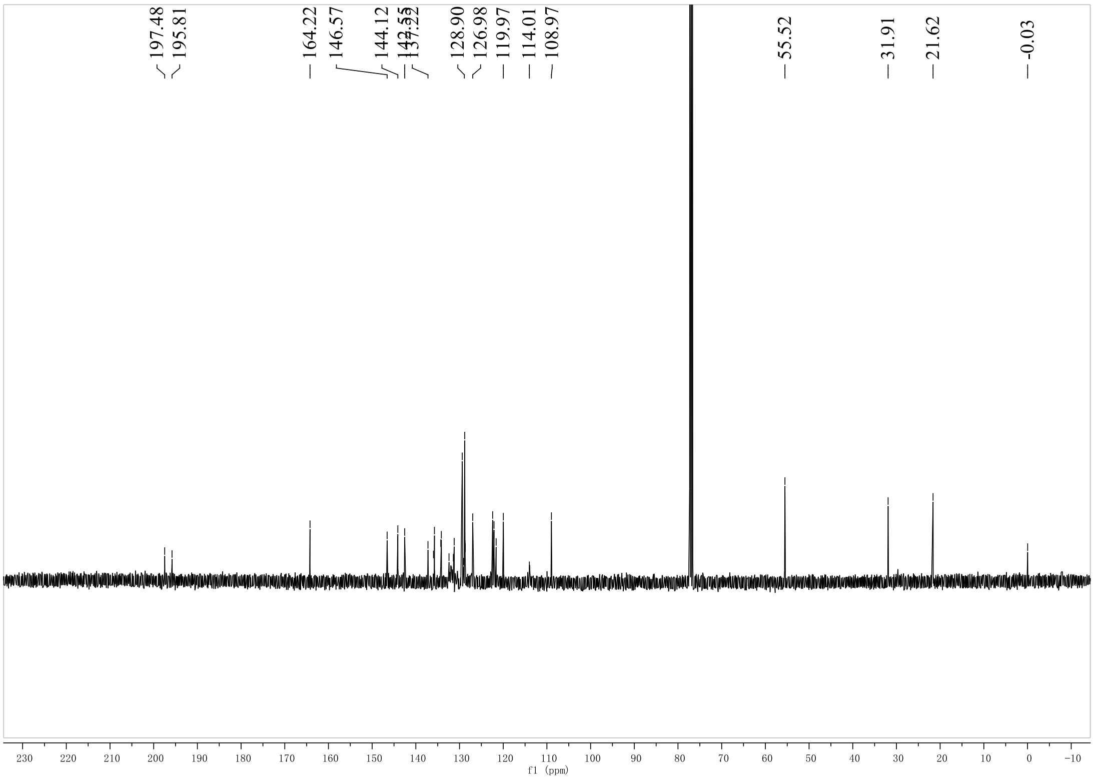
197.48
195.81
164.22
146.57
144.12
137.22
128.90
126.98
119.97
114.01
108.97
55.52
31.91
21.62
-0.03
f1 (ppm)

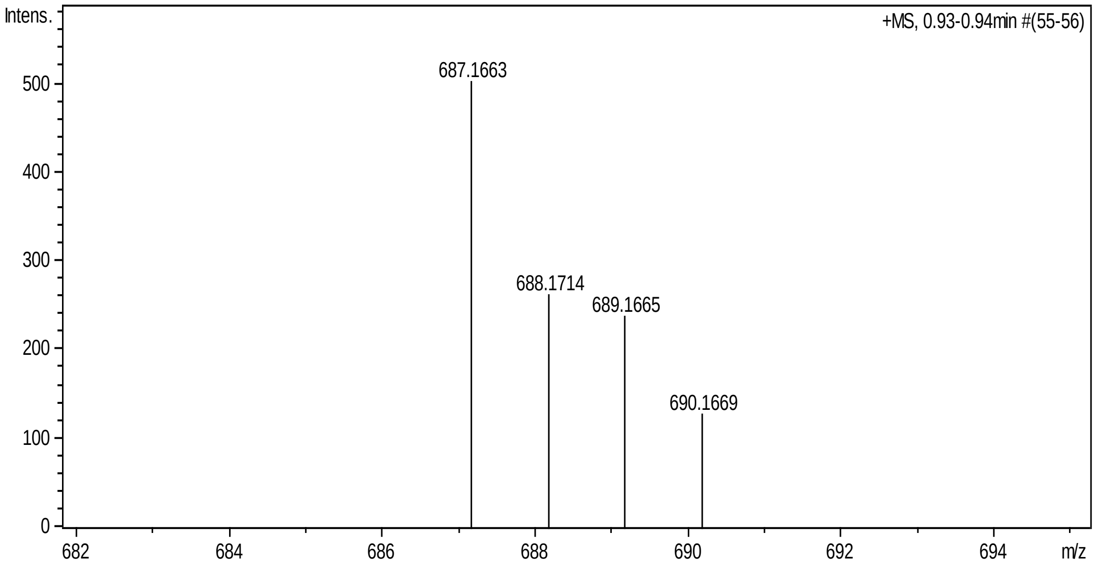
Intens.
+MS, 0.93-0.94min #(55-56)
687.1663
688.1714
689.1665
690.1669
m/z

(1-(4-Chlorobenzoyl)-4-(2-chlorophenyl)-2-(4-methoxyphenyl)-9-methyl-9*H*-carbazol-3-yl)(*p*-tolyl)methanone) (6j):

White solid, 206 mg, 63%, m.p. 187-188 °C; ^{1}H NMR (400 MHz, $CDCl_3$) δ: 7.58 (d, *J* = 8.4 Hz, 2H, ArH), 7.45 (t, *J* = 8.0 Hz, 2H, ArH), 7.38 (d, *J* = 8.0 Hz, 2H, ArH), 7.29-7.23 (m, 5H, ArH), 7.00 (t, *J* = 7.6 Hz, 2H, ArH), 6.92 (d, *J* = 7.6 Hz, 3H, ArH), 6.87 (d, *J* = 8.0 Hz, 2H, ArH), 6.41 (d, *J* = 5.6 Hz, 2H, ArH), 3.62 (s, 3H, CH_3), 3.58 (s, 3H, OCH_3), 2.25 (s, 3H, CH_3); ^{13}C NMR (400 MHz, $CDCl_3$) δ: 158.5, 143.4, 142.3, 139.8, 138.4, 137.4, 136.9, 136.0, 134.7, 134.4, 133.9, 132.5, 132.0, 130.8, 129.3, 128.7, 128.6, 128.6, 128.5, 126.5, 122.2, 121.8, 121.4, 119.7, 112.7, 112.6, 112.5, 108.8, 54.9, 32.1, 21.5; IR(KBr) υ: 3054, 2932, 2362, 1661, 1431, 1322, 1261, 1131, 1008, 937, 803, 727, 642, 478 cm^{-1}; MS (*m*/*z*): HRMS (ESI) Calcd. for $C_{41}H_{29}Cl_2NO_3$ ($[M+Na]^+$): 676.1417, found: 676.1430.

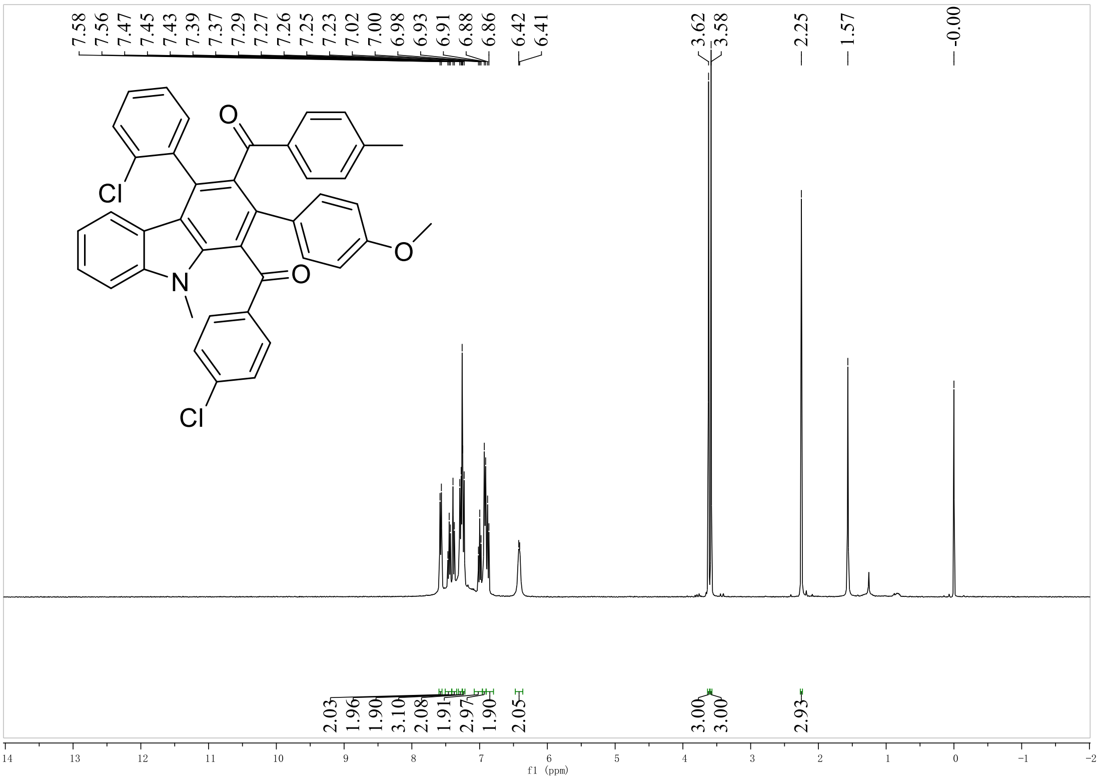

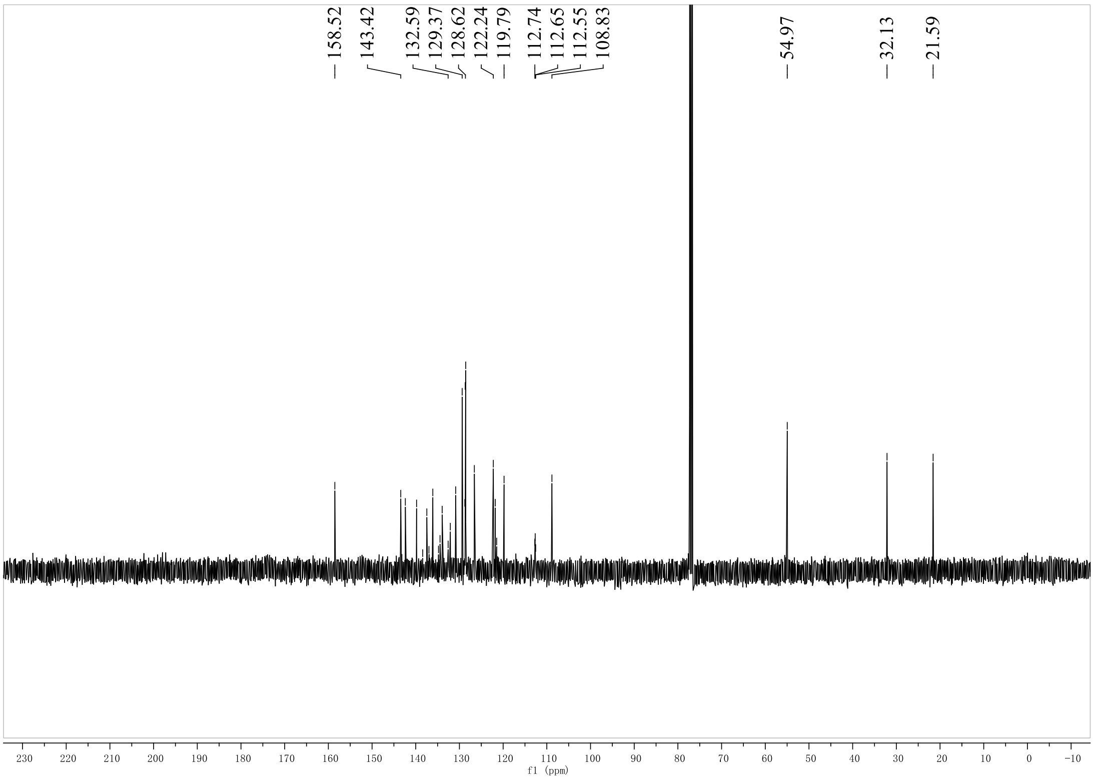

158.52
143.42
132.59
129.37
128.62
122.24
119.79
112.74
112.65
112.55
108.83
54.97
32.13
21.59
230
220
210
200
190
180
170
160
150
140
130
120
110
100
90
80
70
60
50
40
30
20
10
0
−10
f1 (ppm)

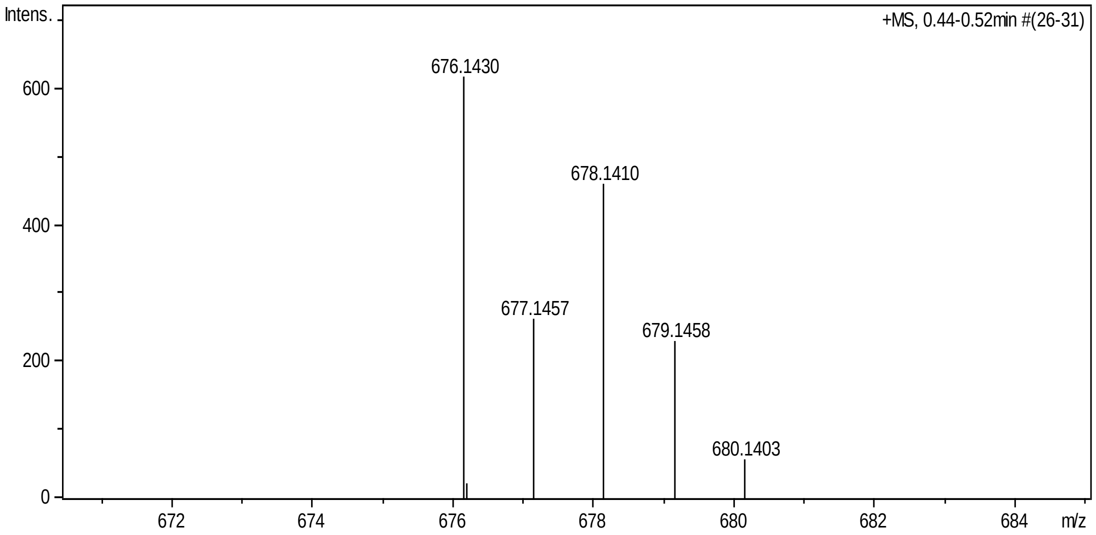

Intens.
+MS, 0.44-0.52min #(26-31)
676.1430
678.1410
677.1457
679.1458
680.1403
600
400
200
0
672
674
676
678
680
682
684
m/z

(3-(4-Chlorobenzoyl)-2-(3-chlorophenyl)-4-(4-methoxyphenyl)-9-methyl-9*H*-carbazol-1-yl)(*p*-tolyl)methanone) (6k):

White solid, 199 mg, 61%, m.p. 184-185 °C; ^{1}H NMR (400 MHz, CDCl$_3$) δ: 7.76 (d, *J* = 30.0 Hz, 2H, ArH), 7.62 (s, 2H, ArH), 7.52-7.39 (m, 4H, ArH), 7.33-7.27 (m, 3H, ArH), 7.23-7.16 (m, 3H, ArH), 7.02 (d, *J* = 7.6 Hz, 1H, ArH), 6.95-6.90 (m, 3H, ArH), 6.77 (d, *J* = 8.4 Hz, 2H, ArH), 3.81 (s, 3H, OCH$_3$), 3.65 (s, 3H, CH$_3$), 2.27 (s, 3H, CH$_3$); ^{13}C NMR (400 MHz, CDCl$_3$) δ: 197.2, 159.2, 142.4, 138.8, 138.5, 137.5, 137.2, 136.2, 135.6, 133.3, 132.9, 131.3, 131.3, 131.1, 130.4, 129.6, 129.4, 129.1, 128.2, 128.1, 127.2, 126.6, 122.5, 122.2, 122.0, 119.7, 113.8, 113.8, 108.7, 55.1, 32.0, 21.7; IR(KBr) υ: 3055, 2932, 2363, 1664, 1430, 1320, 1262, 1135, 1011, 937, 802, 727, 642, 474 cm^{-1}; MS (*m/z*): HRMS (ESI) Calcd. for $C_{41}H_{29}Cl_2NO_3$ ([M+Na]$^+$): 676.1417, found: 676.1420.

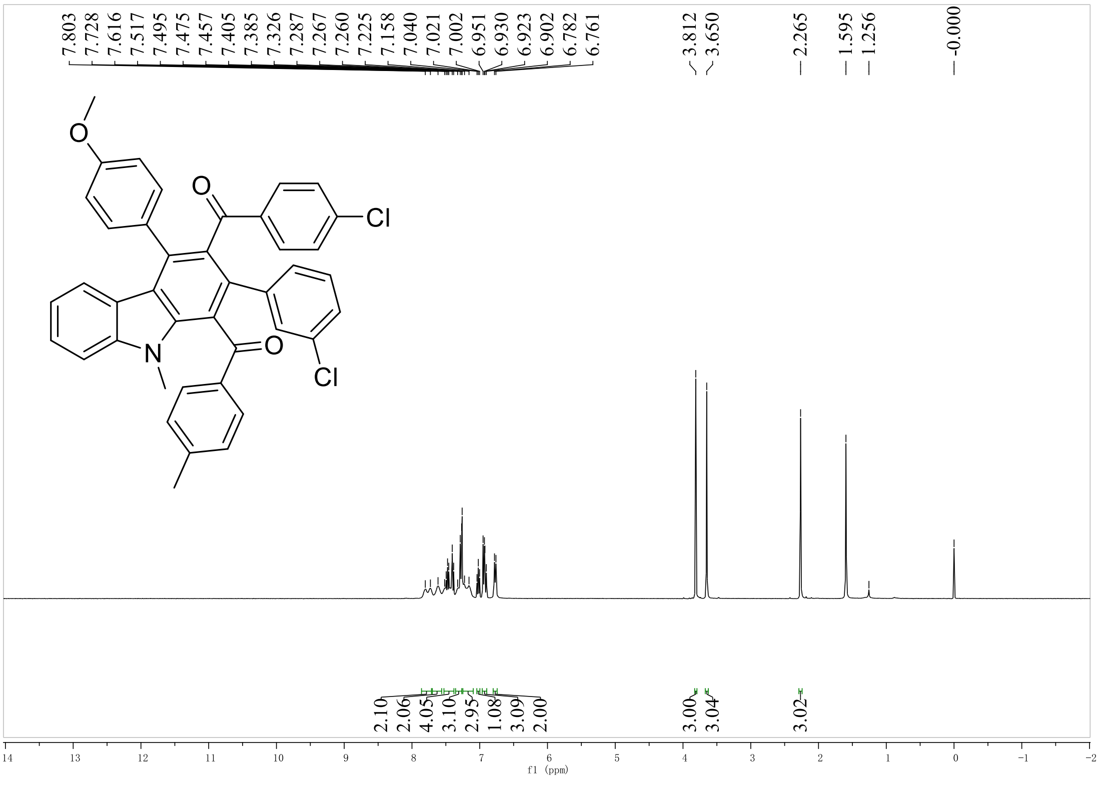

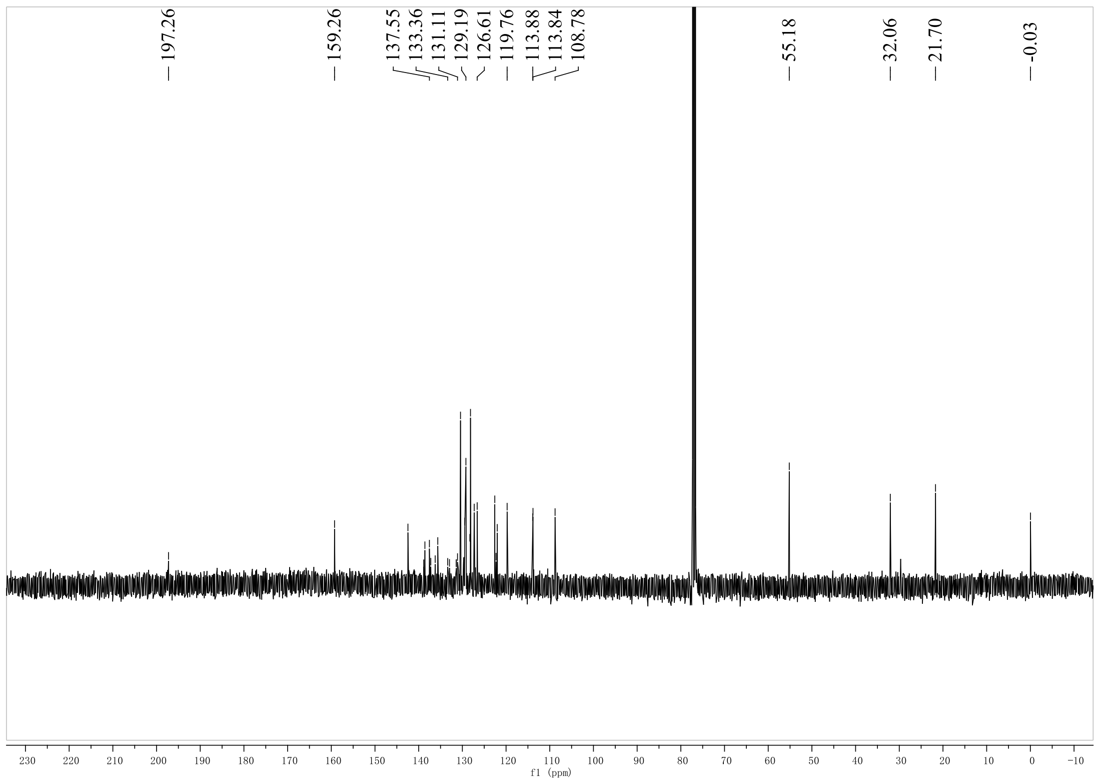
197.26
159.26
137.55
133.36
131.11
129.19
126.61
119.76
113.88
113.84
108.78
55.18
32.06
21.70
-0.03
f1 (ppm)

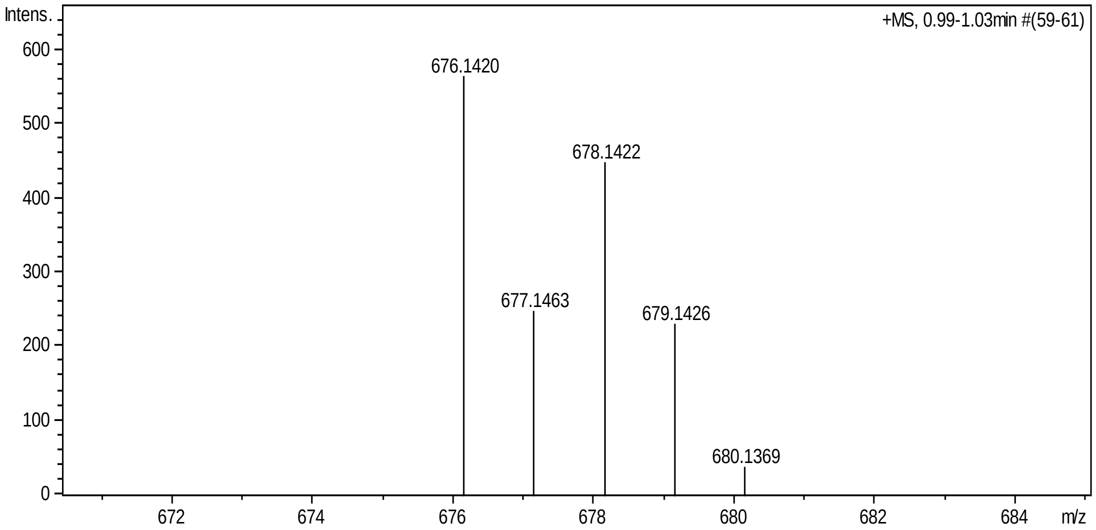
Intens.
+MS, 0.99-1.03min #(59-61)
676.1420
678.1422
677.1463
679.1426
680.1369
m/z

(2,4-Bis(4-methoxyphenyl)-9-methyl-9*H*-carbazole-1,3-diyl)bis((4-chlorophenyl)methanone) (6l):

White solid, 207 mg, 62%, m.p. 165-166 °C; ^{1}H NMR (400 MHz, $CDCl_3$) δ: 7.58 (d, *J* = 8.4 Hz, 2H, ArH), 7.44 (t, *J* = 7.6 Hz, 2H, ArH), 7.37 (d, *J* = 8.4 Hz, 1H, ArH), 7.35 (d, *J* = 8.0 Hz, 3H, ArH), 7.24 (d, *J* = 8.8 Hz, 2H, ArH), 7.09 (d, *J* = 8.8 Hz, 2H, ArH), 6.99 (t, *J* = 7.6 Hz, 2H, ArH), 6.95-6.90 (m, 4H, ArH), 6.43 (d, *J* = 8.0 Hz, 2H, ArH), 3.83 (s, 3H, OCH_3), 3.62 (s, 3H, CH_3), 3.60 (s, 3H, OCH_3); ^{13}C NMR (400 MHz, $CDCl_3$) δ: 159.2, 158.6, 142.3, 139.8, 138.6, 137.6, 135.8, 134.7, 131.6, 131.1, 130.8, 130.4, 129.5, 128.7, 128.6, 128.2, 128.0, 126.4, 125.1, 122.9, 122.5, 122.0, 122.0, 119.7, 113.8, 112.7, 109.9, 108.7, 55.1, 55.0, 32.1; IR(KBr) υ: 3053, 2935, 2361, 1664, 1430, 1320, 1262, 1135, 1010, 937, 802, 728, 644, 477 cm^{-1}; MS (*m*/*z*): HRMS (ESI) Calcd. for $C_{41}H_{29}Cl_2NO_4$ ($[M+Na]^+$): 692.1366, found: 692.1359.

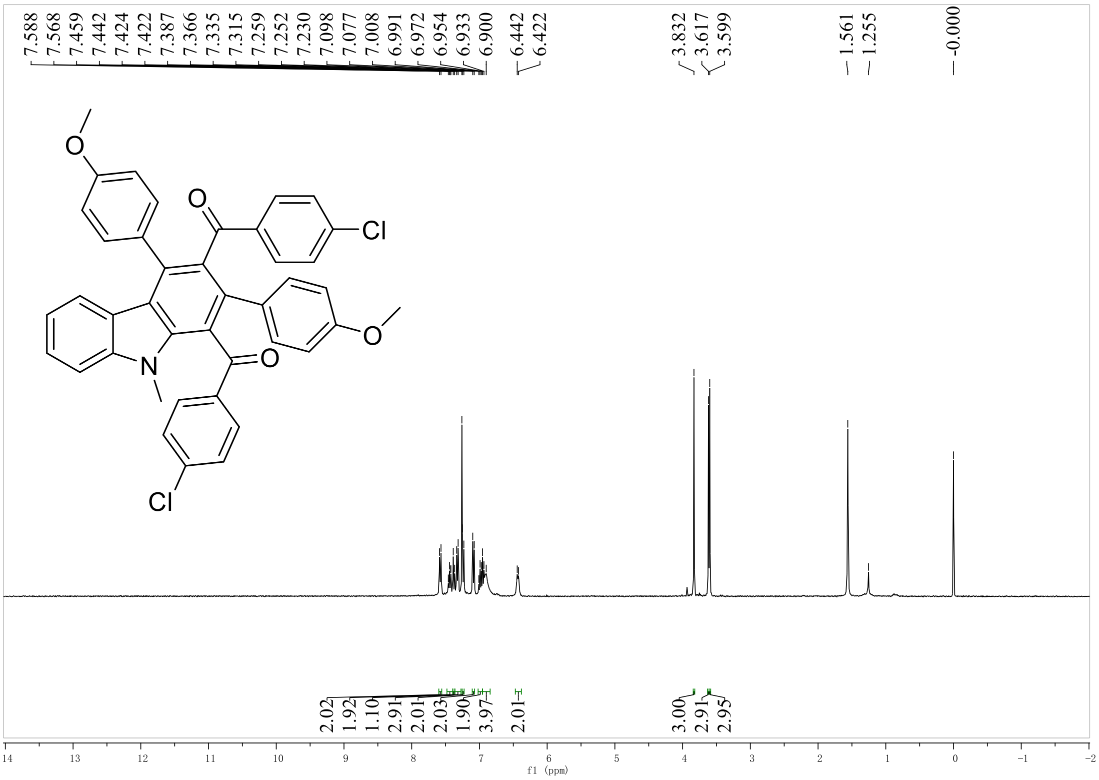

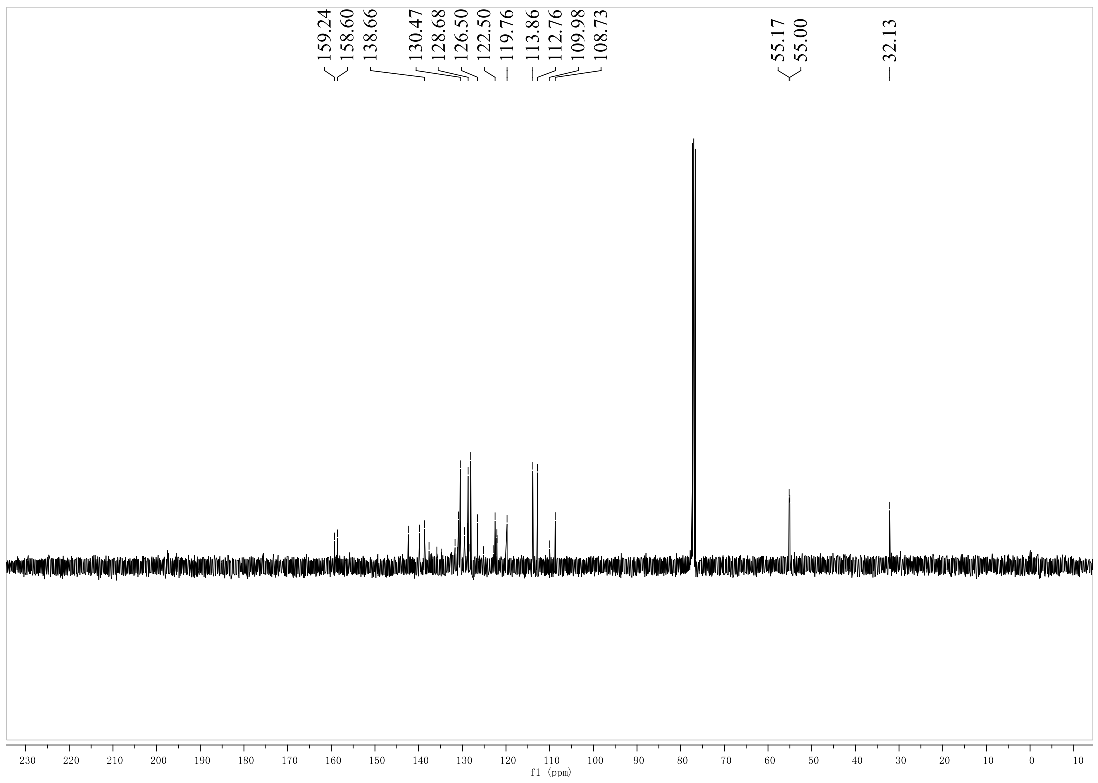
159.24
158.60
138.66
130.47
128.68
126.50
122.50
119.76
113.86
112.76
109.98
108.73
55.17
55.00
32.13
f1 (ppm)

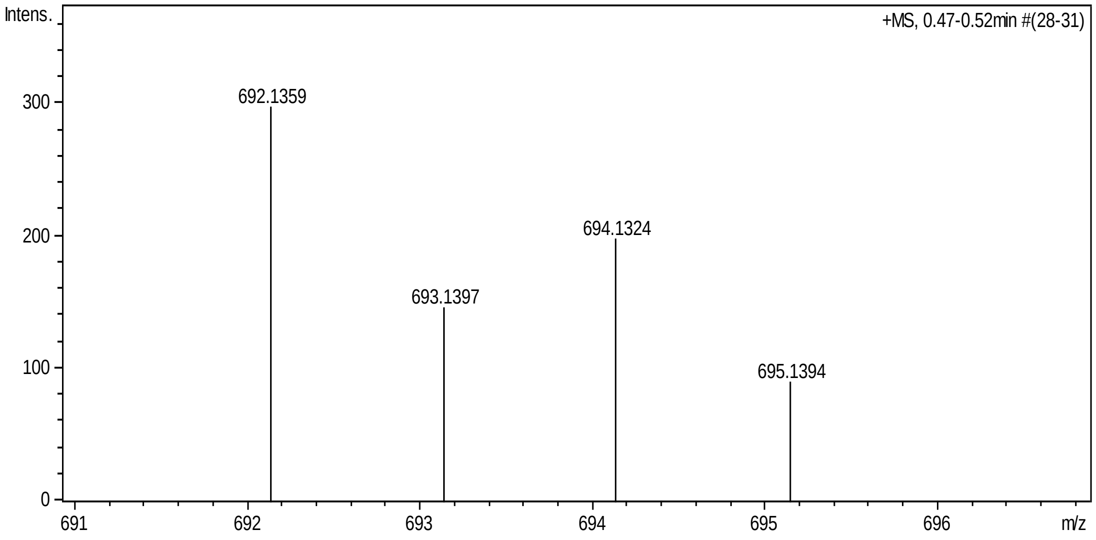
Intens.
+MS, 0.47-0.52min #(28-31)
692.1359
693.1397
694.1324
695.1394
m/z

(1-(3-Benzoyl-9-methyl-2,4-diphenyl-9*H*-carbazol-1-yl)ethan-1-one) (6m):

White solid, 74 mg, 31%, m.p. 154-155 °C; ^{1}H NMR (400 MHz, $CDCl_3$) δ: 7.46-7.41 (m, 3H, ArH), 7.36 (d, *J* = 7.2 Hz, 3H, ArH), 7.32-7.26 (m, 4H, ArH), 7.25-7.15 (m, 5H, ArH), 7.01 (t, *J* = 7.6 Hz, 2H, ArH), 6.96-6.92 (m, 1H, ArH), 6.77 (d, *J* = 8.0 Hz, 1H, ArH), 3.78 (s, 3H, CH_3), 2.12 (s, 3H, CH_3); ^{13}C NMR (400 MHz, $CDCl_3$) δ: 206.2, 198.4, 142.5, 138.9, 137.4, 137.3, 136.1, 135.7, 133.7, 132.2, 131.5, 131.1, 129.1, 128.2, 128.2, 127.8, 127.7, 127.7, 127.6, 126.4, 124.9, 122.3, 122.1, 121.9, 119.6, 108.7, 33.8, 32.3; IR(KBr) υ: 3052, 2923, 2343, 1666, 1653, 1421, 1332, 1266, 1134, 1009, 937, 802, 728, 641, 433 cm^{-1}; MS (*m/z*): HRMS (ESI) Calcd. for $C_{34}H_{25}NO_2$ ($[M+Na]^+$): 502.1778,found: 502.1780.

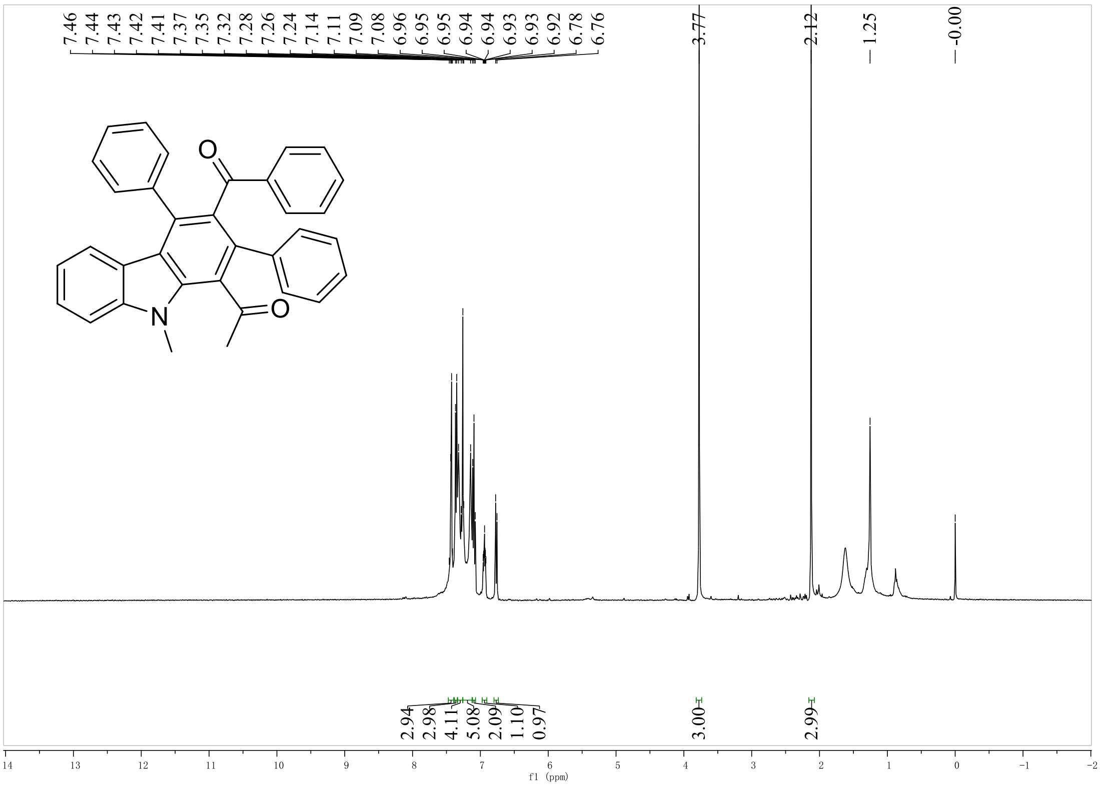

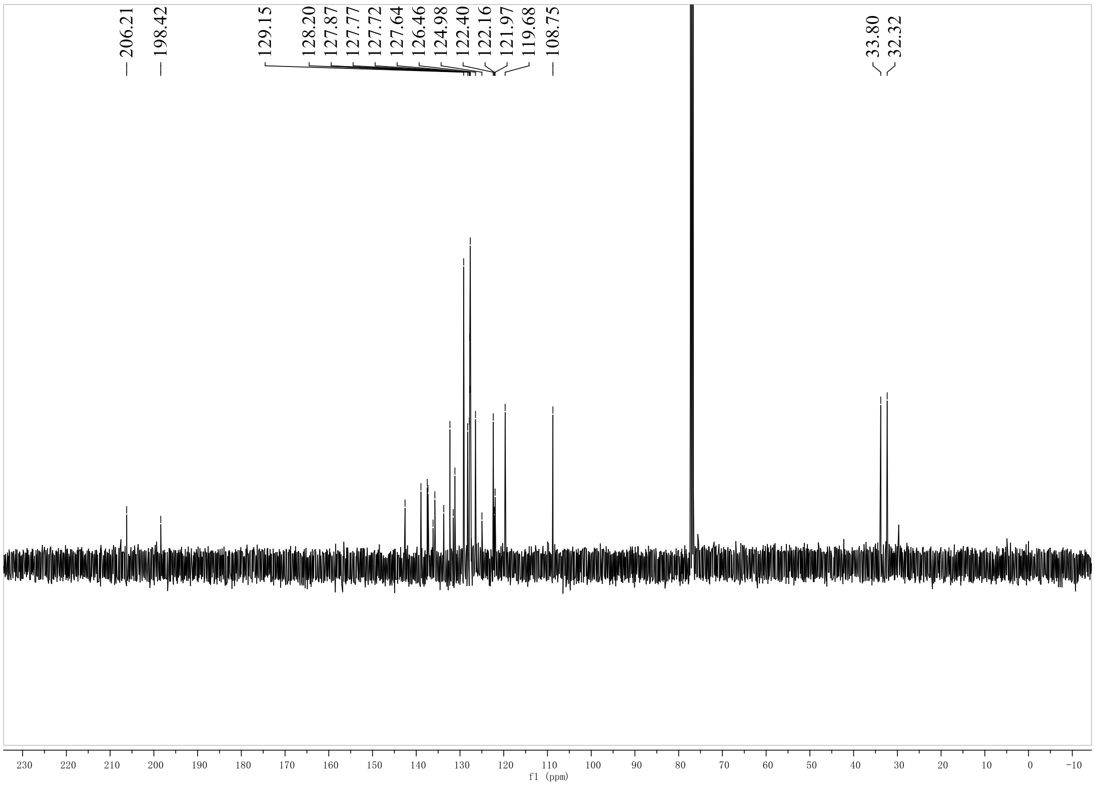

206.21
198.42
129.15
128.20
127.87
127.77
127.72
127.64
126.46
124.98
122.40
122.16
121.97
119.68
108.75
33.80
32.32
230 220 210 200 190 180 170 160 150 140 130 120 110 100 90 80 70 60 50 40 30 20 10 0 -10
f1 (ppm)

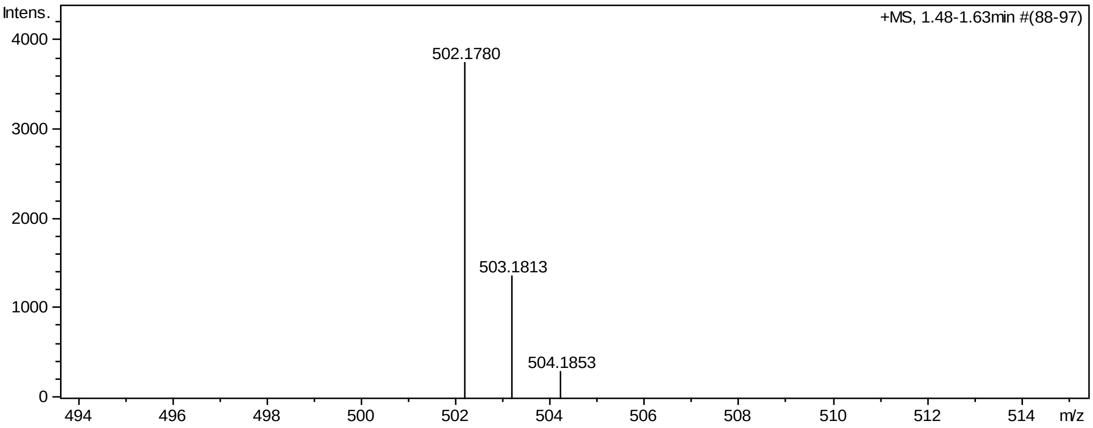

Intens.
+MS, 1.48-1.63min #(88-97)
502.1780
503.1813
504.1853
4000
3000
2000
1000
0
494 496 498 500 502 504 506 508 510 512 514 m/z

(1-(4-(4-Chlorophenyl)-9-methyl-3-(4-methylbenzoyl)-2-phenyl-9*H*-carbazol-1-yl)ethan-1-one) (6n):

White solid, 84 mg, 32%, m.p. 162-163 °C; ^{1}H NMR (400 MHz, $CDCl_3$) δ: 7.48-7.42 (m, 3H, ArH), 7.40-7.30 (m, 3H, ArH), 7.27-7.26 (m, 2H, ArH), 7.25 (s, 1H, ArH), 7.21-7.15 (m, 4H, ArH), 7.01-6.97 (m, 1H, ArH), 6.91 (d, *J* = 8.0 Hz, 2H, ArH), 6.82 (d, *J* = 8.0 Hz, 1H, ArH), 3.77 (s, 3H, CH_3), 2.25 (s, 3H, CH_3) , 2.11 (s, 3H, CH_3); ^{13}C NMR (400 MHz, $CDCl_3$) δ: 206.1, 197.7, 143.3, 142.5, 137.1, 136.2, 136.0, 136.0, 134.1, 133.9, 133.6, 131.7, 131.1, 129.3, 128.5, 128.5, 128.5, 127.7, 127.7, 126.6, 125.2, 122.2, 121.9, 121.7, 119.7, 108.8, 33.7, 32.3, 21.5; IR(KBr) υ: 3051, 2923, 2341, 1667, 1653, 1421, 1331, 1266, 1132, 1009, 937, 802, 728, 643, 435 cm^{-1}; MS (*m*/*z*): HRMS (ESI) Calcd. for $C_{35}H_{26}ClNO_2$ ([M+Na]$^+$): 550.1544, found: 550.1540.

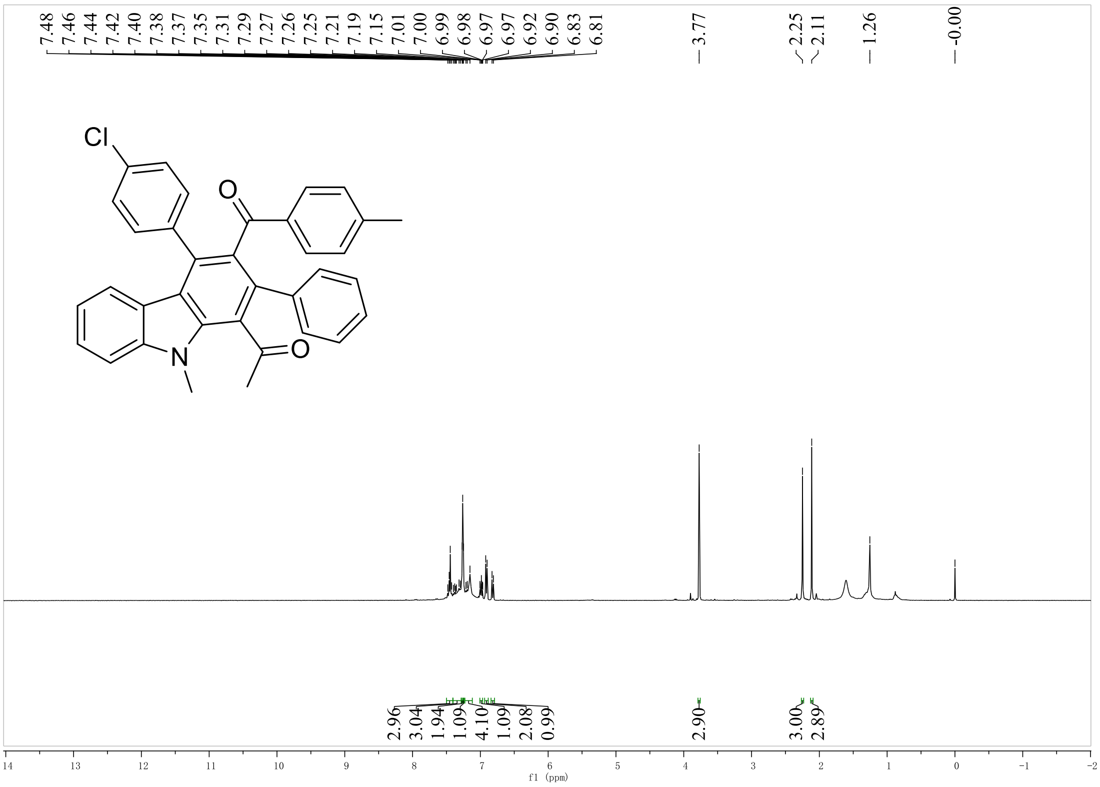

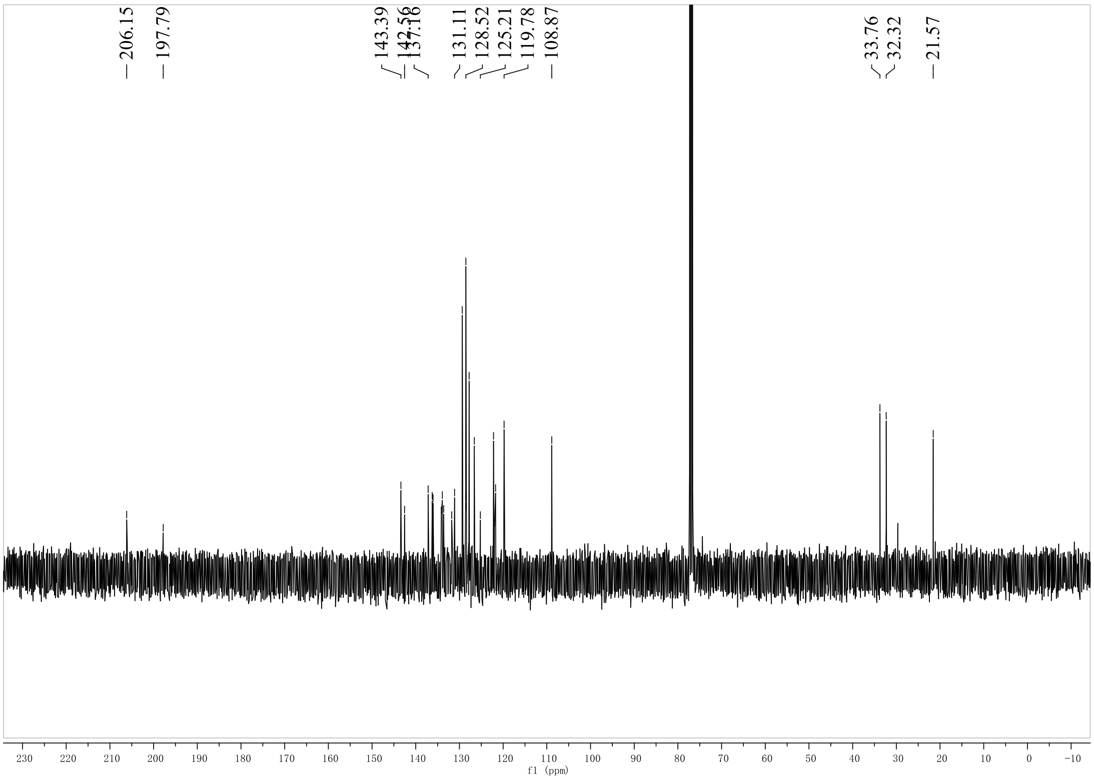
206.15
197.79
143.39
142.56
137.16
131.11
128.52
125.21
119.78
108.87
33.76
32.32
21.57
230 220 210 200 190 180 170 160 150 140 130 120 110 100 90 80 70 60 50 40 30 20 10 0 −10
f1 (ppm)

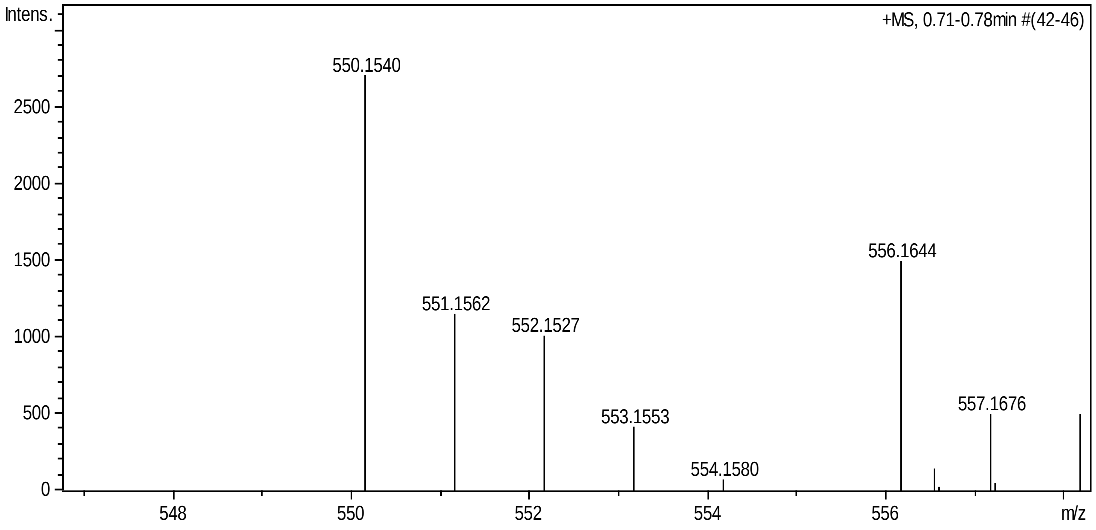
Intens.
+MS, 0.71-0.78min #(42-46)
550.1540
551.1562
552.1527
553.1553
554.1580
556.1644
557.1676
0
500
1000
1500
2000
2500
548
550
552
554
556
m/z
